# Supplementary material for: Pan-cancer whole-genome comparison of primary and metastatic solid tumours
Source: Nature. 2023 May 10;618(7964):333–41. doi: 10.1038/s41586-023-06054-z (PMC10247378; doi:10.1038/s41586-023-06054-z)
Supplement: Supplementary file 1 — This file contains Supplementary Notes 1-4 [file 41586_2023_6054_MOESM1_ESM.pdf]

nature portfolio
https://doi.org/10.1038/s41586-023-06054-z

Supplementary information

Pan-cancer whole-genome comparison of primary and metastatic solid tumours

In the format provided by the authors and unedited

Outline of the Supplementary Information

Supplementary Note 1 **1**
Cohort gathering and processing 2
Datasets 2
Variant calling 2
Sample inclusion criteria 3
Impact of sequencing depth coverage and analysis pipeline on somatic variant calling sensitivity 7
Downsampling 7
The impact of clonality on variant calling 15
The impact of pipeline differences on variant calling 18

Supplementary Note 2 **22**
Introduction 23
Primary and metastatic comparison of genomic features by breast, colon and uterus carcinoma subtypes 24
Dataset annotation 24
Tumor clonality 25
Karyotype and genomic instability indicators 26
Tumor mutation burden and mutational signatures 29
Structural variant burden 31
Drivers 33
Conclusion 35
Primary and metastatic comparison of genomic features by primary clinical progression status 36
Dataset annotation 36
Tumor clonality 36
Karyotype and genomic instability indicators 37
Tumor mutation burden and mutational signatures 39
Structural variant burden 43
Drivers 45
Conclusion 48
Primary and metastatic comparison of genomic features by metastatic biopsy location 48
Dataset annotation 48
Tumor clonality 49
Karyotype and genomic instability indicators 49
Tumor mutation burden and mutational signatures 54
Structural variant burden 59
Drivers 63

Conclusion 70

Supplementary Note 3 **72**

Introduction 73

Ploidy correction 73

Clonality of mutations 74

Breast carcinoma subtypes 75

Differences across different metastatic biopsy location 76

SBS5/SBS40 mutational processes comparison 77

SBS1 mutation burden in metastatic prostate samples from the PCAWG cohort 77

Clonality and timing of SBS1 mutations 78

Clonality and timing of SBS5/SBS40 mutations 81

Independent datasets description 83

Independent validation in using paired biopsies 84

Breast cancer dataset (Lucy R Yates et al.) 84

Kidney renal clear cell carcinoma dataset (TRACERx renal dataset) 87

Independent validation using external primary and metastatic datasets 90

Breast carcinoma 90

Prostate carcinoma 91

Kidney renal clear cell carcinoma 94

Thyroid carcinoma 95

Colorectal and ovarian serous carcinomas 96

Conclusions 98

Appendix 1: Exome-only mutations in Hartwig dataset 98

Supplementary Note 4 **99**

Overview 100

TEDs rationale and limitations 100

Prognostic and resistance TEDs 100

TEDs and primary targets of targeted therapies 100

References **104**

Supplementary Note 1

Cohort gathering and processing 2
Datasets 2
Variant calling 2
Sample inclusion criteria 3
Impact of sequencing depth coverage and analysis pipeline on somatic variant calling sensitivity 7
Downsampling 7
The impact of clonality on variant calling 15
The impact of pipeline differences on variant calling 18

Cohort gathering and processing

Datasets

We have matched tumor-normal paired whole genome sequencing data from cancer patients from two cohorts: the Hartwig Medical Foundation (Hartwig) and the Pan-Cancer Analysis of Whole Genomes (PCAWG) cohort.

The Hartwig cohort contained patient data for which re-use for cancer research was consented by the patients and was provided under data transfer agreement (DR-247) by Hartwig Medical Foundation. The Hartwig cohort included 4,776 metastatic tumor samples from 4,460 patients. This national initiative consists of nearly 50 oncology centers inThe Netherlands and aims to improve personalized cancer treatment. To this end, Hartwig Medical Foundation sequences and characterizes the genomic landscape for a large number of patients both in clinical research projects as well as routine diagnostics (including consenting for data re-use). Furthermore, genomics data is integrated with clinical data which consists of primary tumor type, biopsy location, gender, pretreatment type before biopsy, and treatment type after biopsy. For patients with multiple biopsies taken at different timepoints, patient IDs were suffixed by a letter for the different biopsies (e.g. HMF001423A, HMF001423B). Normal samples (blood) had a mean read coverage of ~38x while tumor samples had a mean coverage of ~109x [1]. A detailed description of the consortium and the whole patient cohort has been described in detail in Priestley et al.[1]

The PCAWG cohort consisted of 2,835 patient tumors, and access for raw sequencing data for the PCAWG-US was approved by National Institutes of Health (NIH) for the dataset General Research Use in The Cancer Genome Atlas (TCGA) on 25 February 2021 under application number 100344-3 and downloaded via dbGAP download portal. Raw sequencing access to the non-US PCAWG samples was granted via the Data Access Compliance Office (DACO) Application Number DACO-1050905 on 6 October 2017 and downloaded via https://console.cancercollaboratory.org on 4 December 2017. The most recent clinical data was downloaded from the PCAWG release page (https://dcc.icgc.org/releases/PCAWG/) on Aug 2021. Normal samples (blood, adjacent tumors or distant tumors) had a mean read coverage of 39x, while tumor samples had a bimodal coverage distribution with modes at 38x and 60x. A detailed description of the consortium and the whole patient cohort has been described in detail in the PCAWG flagship paper [2].

Variant calling

Somatic mutation data of the Hartwig cohort were provided on 6 February 2020 and were updated on 20 October 2021. The bam files from PCAWG cohort were reformatted to FASTQ files (SamToFastq PICARD v2.1.0) which were realigned to reference genome GRCH37 lacking the GL0000XX contigs (BWA-mem v0.7.17[3]). These FASTQ files were used as input for the standard Hartwig analysis workflow to exclude technical noise from PCAWG and Hartwig somatic calling workflows. The Hartwig somatic calling pipeline (https://github.com/hartwigmedical/pipeline5) is

hosted on a Google Cloud platform using platinum (https://github.com/hartwigmedical/platinum) that enables to run the complete Hartwig pipeline at once. A full pipeline description is explained in refs[1,4], and details and settings of all the tools can be found on their Github page (https://github.com/hartwigmedical/hmftools). Briefly, reads were mapped to GRCh37 using BWA (v0.7.17). GATK (v3.8.0) Haplotype Caller was used for calling germline variants in the reference sample. SAGE (v2.2) was used to call somatic single and multi nucleotide variants as well as indels. GRIDSS (v2.9.3) [5] was used to call simple and complex structural variants. PURPLE combines B-allele frequency (BAF) from AMBER (v3.3), read depth ratios from COBALT (v1.7), and structural variants from GRIDSS to estimate copy number profiles, variant allele frequency (VAF) and variant clonality. LINX (v1.16) [6] interprets structural variants (to identify simple and complex structural events) from PURPLE (v2.53), and also detects gene fusions, viral DNA integrations, and homozygously disrupted genes. Importantly, we ensured that mutation (simple and complex) filtering and annotation tools were run with the same versioning for PCAWG and HMF cohort.

Sample inclusion criteria

A selection of samples for all analyses was made based on several criteria.

To exclude duplicate samples from the same patient for the Hartwig cohort, we selected the tumor sample with the most recent biopsy date, and if this information did not exist we selected the sample with the highest tumor purity. However, some patients had biopsies from different primary tumor locations (likely independent or secondary tumors). In these cases, we kept at least one sample from each primary tumor location, and when there were multiple samples from the same primary tumor location, we applied the aforementioned biopsy date and tumor purity filtering criteria. For the PCAWG cohort, we processed one tumor sample per donor and tumor sample IDs are included in Supp. Table 1 of the manuscript. As with Hartwig QC filter criteria, samples with a tumor purity lower than 20% were excluded as somatic variant calling was less reliable for these samples. PCAWG samples that were gray- or blacklisted by the PCAWG consortium were also removed (see https://dcc.icgc.org/releases/PCAWG/donors_and_biospecimens). For both cohorts, we only kept samples with >=50 SNVs/indels (likely no tumor cells present in the sample), and removed an additional set of samples for several reasons including failed variant calling, insufficient informed consent for use of the WGS data (33 Hartwig samples were excluded during revision process), unnatural SV landscape, and one duplicate PCAWG patient (DO217844) that was also included in the Hartwig cohort. After strict QC filtering, the PCAWG whitelisted cohort includes 2,376 samples and this dataset has been made available for the cancer research community via the PCAWG resource page: https://dcc.icgc.org/releases/PCAWG/Hartwig.

Besides the exclusion criteria explained above, we excluded an additional set of 386 samples from the Hartwig cohort during the revision process to mitigate cancer cohort heterogeneity with the PCAWG dataset. For this, we performed an extensive harmonization of the clinical information of all patients and relied as much as possible on the clinical cancer type definition previously used

in the TCGA [7] and PCAWG [2] pan-cancer studies. Following these cancer subtype annotations, we excluded samples from the Hartwig dataset that could not be attributed to one of these 23 cancer type categories. In detail, we followed the next criterias:

- Lung adenocarcinoma (LUAD) and Lung squamous cell carcinoma (LUSC) are two independent cancer types.
- To prevent cancer subtype intrinsic differences across renal cell carcinomas we focussed our comparison to the most prevalent subtype, renal clear cell carcinoma. Metastatic renal cancer patients from other renal cell carcinomas subtypes (e.g., papillary) were not included in the comparison both in the primary and metastatic cohorts.
- Similarly, we narrowed the definition of ovarian cancer to ovarian serous carcinomas. Consequently, patients with metastatic ovarian cancer lacking this cancer type annotation were omitted.
- Pancreatic neuroendocrine patients were restrained to well-differentiated pancreatic neuroendocrine tumors.
- For consistency with the PCAWG cohort, uterus carcinoma was limited to uterus endometrial carcinomas.
- For breast carcinoma we have annotated the breast carcinoma subtypes for primary and metastatic tumors (i.e., ER-/HER2-, ER-/HER2+, ER+/HER2+ and triple negative breast cancer). The comparison by breast cancer subtype is part of the Supplementary Note 2.
- For colorectal and uterus carcinomas, we have annotated the MSI/POLE status. The comparison splitting by MSS and MSI/POLE groups is part of the Supplementary Note 2.
- For the rest of cancer types, we have discarded metastatic tumor samples with neuroendocrine or sarcomatoid subtypes. This included samples with any recorded pathological annotations of “neuroendocrine” (except for pancreas neuroendocrine and gastrointestinal neuroendocrine) and “sarcoma” (except for liposarcoma, leiomyosarcoma, and osteosarcoma).

Hartwig cancer cohort	Samples excluded for clinical annotation	Hartwig cohort Size
Bladder urothelial carcinoma	47	128
Breast carcinoma	11	776
Cervical carcinoma	2	38
Colorectal carcinoma	7	621
Hepatocellular carcinoma	1	52
Kidney renal clear cell carcinoma	19	110
Leiomyosarcoma	1	47
Non Small lung cancer	222	
Lung adenocarcinoma		255
Lung squamous cell carcinoma		34
Ovarian serous adenocarcinoma	64	104
Pancreas carcinoma	4	99
Pancreas neuroendocrine	6	34
Upper respiratory tract carcinoma	1	38
Uterus carcinoma	1	32
**Grand Total**	**386**	**2368**

Lastly, for this study, we only selected samples from cancer types representing at least 15 samples in both PCAWG and Hartwig cohort. This resulted in a final dataset consisting of 3,451 Hartwig samples and 1,914 PCAWG samples.

In summary, we established and shared a uniformly processed pan-cancer dataset of 7,108 tumor WGS samples. Of those, 5,365 samples were used in the current study (1,914 primary and 3,451 metastatic tumors) to compare primary to metastatic tumors. The clinical metadata as well as the clinical, technical and ethical exclusion criteria of each sample, including those selected for analyses, is detailed in Supplementary Table 1 of the manuscript.

Impact of sequencing depth coverage and analysis pipeline on somatic variant calling sensitivity

Genomic comparisons of different WGS cancer cohorts must be conducted with caution because mutation datasets are established by distinct sequencing approaches (e.g. sequencing read depths and platforms) and somatic mutation calling workflows. The PCAWG cohort has been sequenced with an overall lower sequencing coverage (i.e., 38X, 60X and 90X) than the Hartwig cohort (109X) which results in lower number of mutations with default Hartwig somatic mutation pipeline settings optimized for 109X. To assess and mitigate the impact of these confoundings (for instance by applying less stringent mutation calling parameters for 38X and 60X sequenced samples), we first downsampled 25 Hartwig samples to 38X and 60X to assess the loss in mutation calling sensitivity at 38X and 60X sequence depth. Subsequently, we adjusted the SAGE mapping threshold levels to obtain highly similar mutation callsets for each sequence coverage bucket. Finally, we applied these mapping threshold levels settings from the downsampling analysis, to each PCAWG sample analyzed with the Hartwig somatic mutation pipeline depending on the sample sequence depth. Lastly, we also compared the somatic calls obtained with Hartwig pipeline to the PCAWG consensus callset from the PCAWG resource page (https://dcc.icgc.org/releases/PCAWG/).

Downsampling

To validate the effect of sequencing depth on variant calling sensitivity, we selected the following 25 tumor BAM samples at random from the Hartwig cohort (109x) which we downsampled to the bimodal mean coverage depth (38x and 60x) of the PCAWG cohort using sambamba 0.6.8 [8]. [HMF000054, HMF000612, HMF001536, HMF001729, HMF002822, HMF003446, HMF003796, HMF001904, HMF000318, HMF000644, HMF000920, HMF000500, HMF000774, HMF002635, HMF000164, HMF002983, HMF003913, HMF004359, HMF000458, HMF001506, HMF000097, HMF001218, HMF002379, HMF002506, HMF003755]. The matching germline samples were not downsampled because both cohorts represent similar sequencing depths for the control samples (i.e. mean depth of 39X and 38x for PCAWG and Hartwig cohort respectively). We then re-ran the identical somatic variant calling pipeline on those samples and examined variant calls of all somatic mutation types.

We first assessed the global genomic features including the ploidy level, LOH fraction and whole-genome duplication status from PURPLE. This purity ploidy estimator tool combines B-allele frequencies (BAF), read depth ratios, somatic variants and structural variants to estimate the purity and copy number profile of a tumor sample, and provides allele-specific mutation annotations (e.g. purity and ploidy corrected variant allele frequencies (VAFs)). All downsampled samples showed no difference for any of the aforementioned features which demonstrates that the calling of global mutation characteristics is independent of sequencing coverage with a depth of at least 38x (Suppl. Fig. 1).

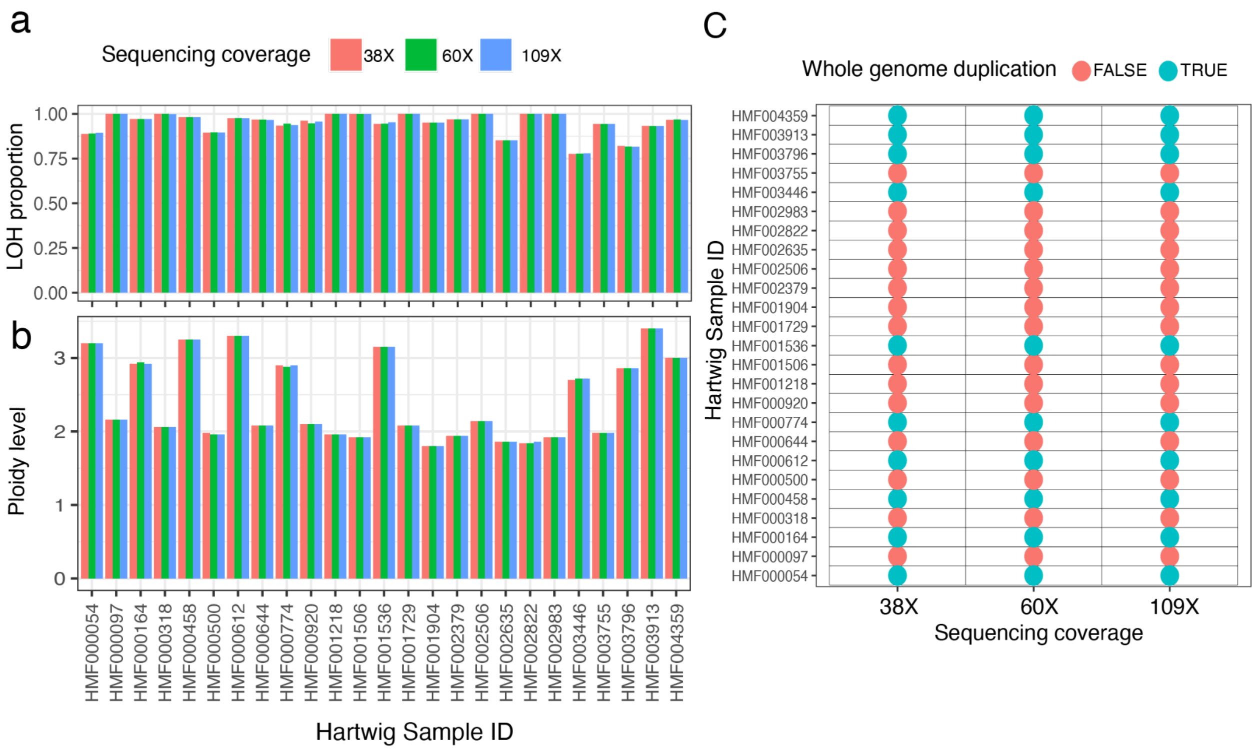

Supplementary Figure 1: Effect of sequencing depth on global genomic features. Comparison of 25 randomly selected 109x sequenced Hartwig samples that were downsampled to 60x and 38x, the modes of the bimodal coverage distribution of the PCAWG project. The loss-of-heterozygosity (LOH) fraction **(a)**, ploidy estimates **(b)** and whole genome duplication scores **(c)** are not impacted by sequencing coverage.

We next evaluated the simple mutations of SAGE, which is the somatic mutation calling algorithm of the Hartwig pipeline. Using SAGE default settings from the Hartwig somatic pipeline we observed a drop in sensitivity with a fold change of 0.26x-0.04x (i.e, 26%-4%), 1.46x-0.56x and 0.35x-0.1x for SBS, DBS and Indels in 38x-60x mode, respectively. This is likely explained by the hardcoded ~5 supporting read threshold within SAGE, because SAGE was developed and optimized for samples being sequenced with at least 100X. To obtain similar mutation calls for samples sequenced at 60X and 38X, we increased the sensitivity for the “min_tumor_qual” flagged mutations which is the QC score for the number of high-quality reads. As such, the HOTSPOT/PANEL/HighConfidence/LowConfidence SAGE filtering settings were set to 70/70/70/90 and 70/100/100/150 for the 38x and 60x-mode respectively for “min_tumor_qual” flagged mutations. We found that these settings yielded highly similar results for the SBS, DBS and indel mutation burden for 38x and 60x (mean fold change increase of x0.00 ±0.03std.) compared to the output of the original runs with 109x depth at default settings (Suppl. Fig. 2a,b).

Next, we calculated the proportion of mutations originally called in the 109x samples that are recapitulated in the downsampled samples, stratified by their original VAF scores. For this, we assessed the mutation recall rate across the three tested sequence depths by calculating the fraction of somatic mutation overlap between 38X and 109X, and 60X and 109X in the 25

downsampled Hartwig samples. Subsequently, we binned the mutation based on its purity adjusted VAF score of the original 109X sample (Suppl. Fig. 2 below).

Overall, we found consistent results. Mutations with VAF scores higher than 0.3 (4th bin onwards) displayed a minimal recall rate of 96% for 38X sequencing depth and 98% for the 60X sequence mode (Suppl. Fig. 2c). This percentage dropped marginally for the DBS (recall rate of at least 96% and 96% at 38X and 60X respectively), and indel (recall rate of at least 89% and 94% at 38X and 60X respectively) mutation types, however these mutation types were not included in any of the the clonality ratio measurements. Mutations with a VAF lower than 0.3 (1st to 3rd bins) showed SNV recall rates of 93% (0.2<VAF<0.3) and 84% (0.1<VAF<0.2) at 38X sequence depth, and 97% (0.2<VAF<0.3) and 91% (0.1<VAF<0.20) at 60X sequence depth. However, low VAF mutations marginally contribute to the TMB for SNV, DBS and INDELs as depicted by the barplots on top.

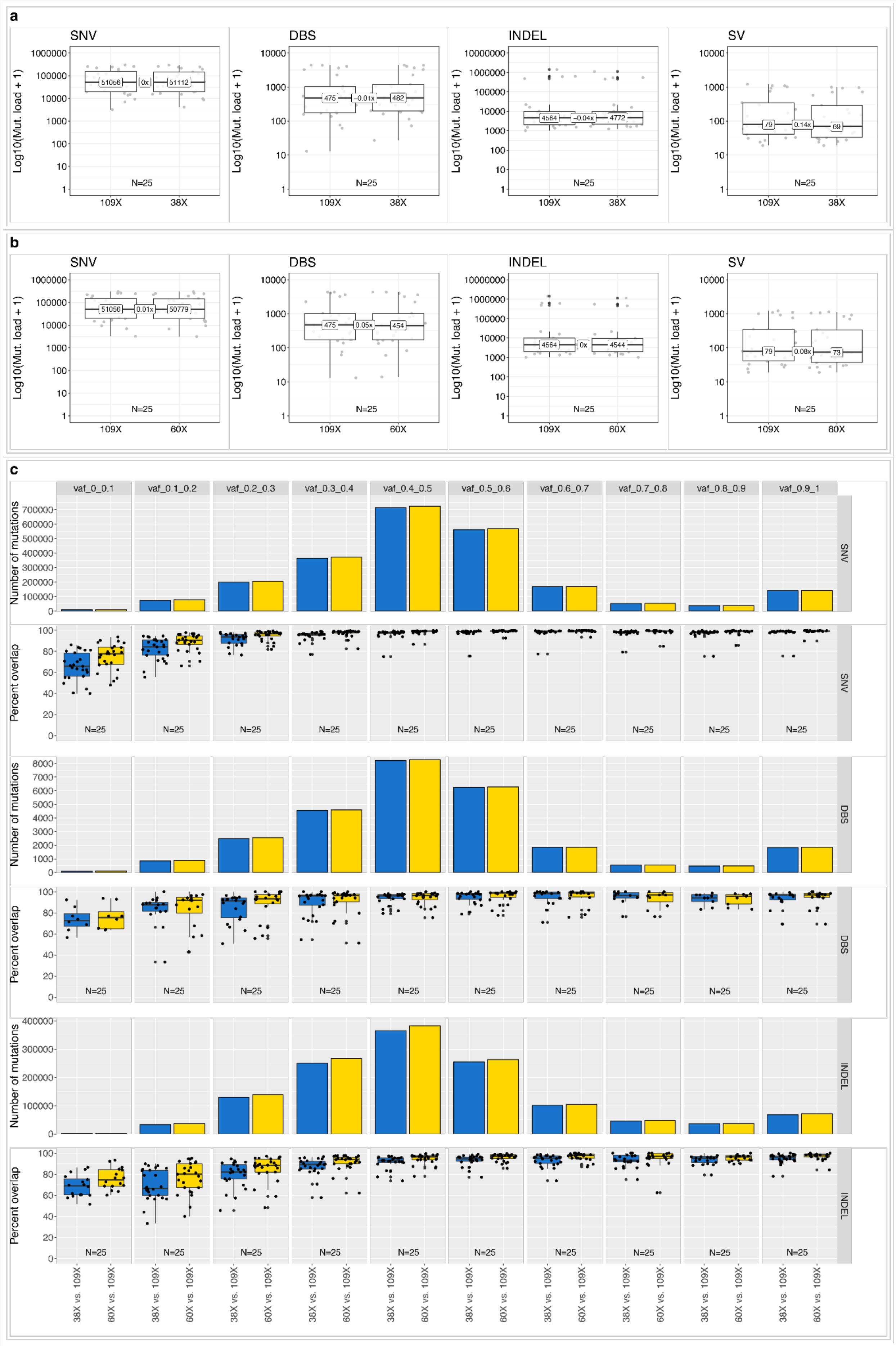
a
SNV
DBS
INDEL
SV
Log10(Mut. load + 1)
51056
0x
51112
475
-0.01x
482
4564
-0.04x
4772
79
0.14x
69
N=25
109X
38X
b
SNV
DBS
INDEL
SV
Log10(Mut. load + 1)
51056
0.01x
50779
475
0.05x
454
4564
0x
4544
79
0.08x
73
N=25
109X
60X
c
vaf_0_0.1
vaf_0.1_0.2
vaf_0.2_0.3
vaf_0.3_0.4
vaf_0.4_0.5
vaf_0.5_0.6
vaf_0.6_0.7
vaf_0.7_0.8
vaf_0.8_0.9
vaf_0.9_1
Number of mutations
Percent overlap
SNV
DBS
INDEL
N=25
38X vs. 109X
60X vs. 109X

Supplementary Figure 2: Effect of sequencing depth on somatic mutations. Tumor mutation burden comparison of 25 randomly selected 109x sequenced Hartwig samples that were downsampled to 38x **(a)** and 60x **(b)** for SNV, DBS, INDEL and SV mutation types. The boxplots show the median mutation burden for each sequencing depth mode as well as the median fold change sensitivity loss for every mutation type. **(c)** Barplots and boxplots showing the mutation recall rate across the three tested sequence depths per mutation type per VAF bin. The boxplots show the median mutation recall rate for each sequencing depth mode and the barplots show the number of mutation per VAF bin. Box-plots: center line, median; box limits, first and third quartiles; whiskers, lowest/highest data points at first quartile minus/plus 1.5× IQR. N, number of randomly selected samples from the PCAWG project.

We also observed a minimal effect on the driver landscape. For the 172 reported drivers in the 25 Hartwig samples sequenced at 109x, 95% and 94% of the drivers were recalled in the 38x and 60x-mode respectively. Given the consistent results in passenger and driver mutation detection over the tested sequencing depth modes, we have applied these two SAGE filter modes to all PCAWG samples depending on their respective sequencing depth bucket to normalize the sensitivity impact on mutation calling between the PCAWG and Hartwig cohort.

Next to simple somatic mutations, we also validated structural variants from LINX [6]. This tool integrates copy number profiles and the SV calls from PURPLE [1] and GRIDSS [5] that enables the clustering and chaining of genomic rearrangements. To evaluate the impact of sequencing depth on SV calling, we analyzed the 60x and 38x downsampled samples and observed a decrease in sensitivity with a fold change of 0.08X and 0.14X (i.e., 8%-14%), respectively (Suppl. Fig. 2a,b). Given the complexity of the integrated SV calling workflow, we have not adjusted threshold settings for the SVs. As a result, particularly samples sequenced with ~38x have a decreased sensitivity to call SVs. However, only a few PCAWG cancer type cohorts consist of a high proportion of 38x samples (Suppl. Fig. 3a). To estimate the effect of sequencing depth on cancer type level for the PCAWG cohort, we have assessed the sensitivity loss at varying proportions of 38x and 60x sequenced samples per cancer type for all mutation types.

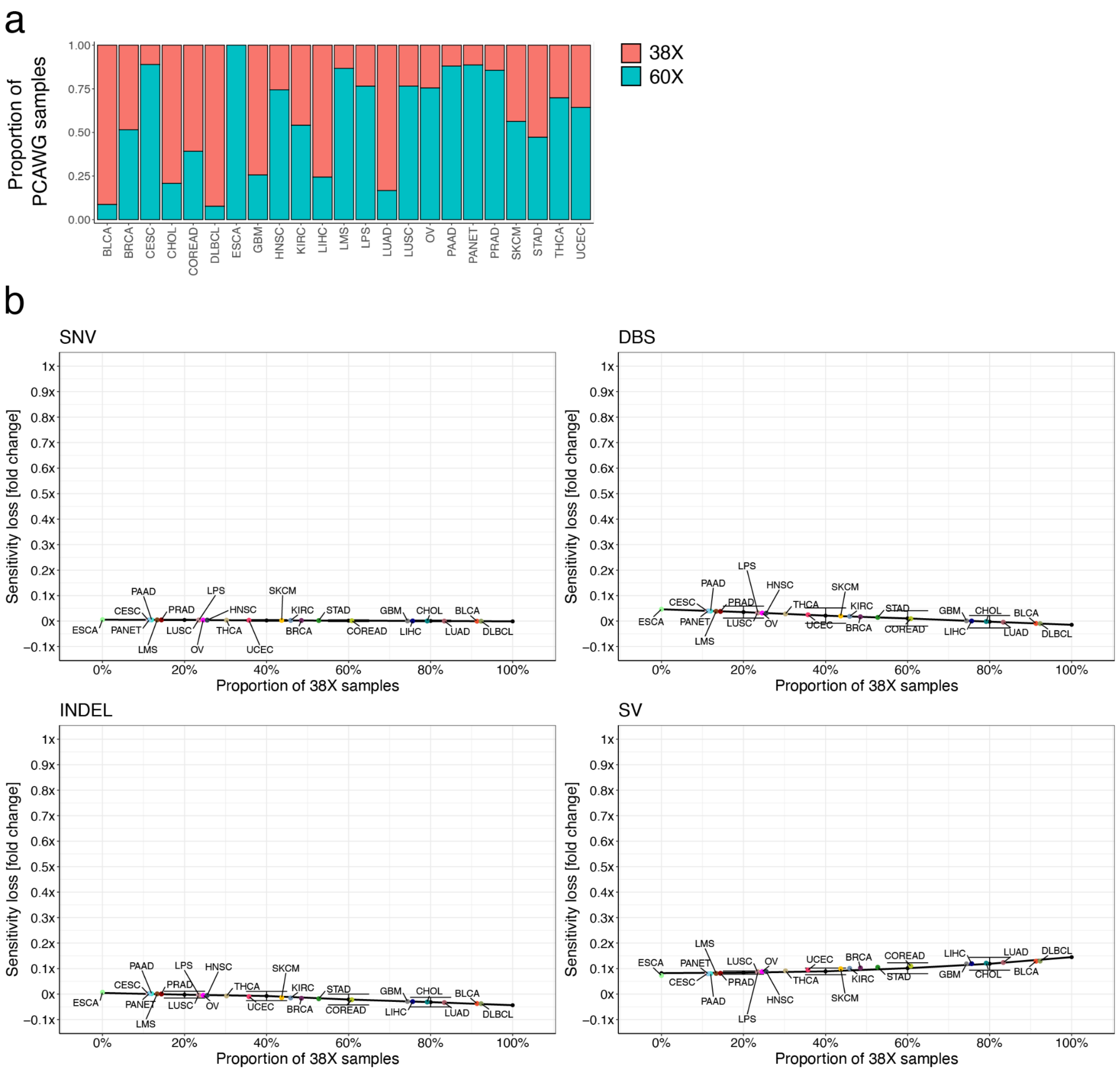

Supplementary Figure 3: Effect of sequencing depth on PCAWG cancer type. (a) Every PCAWG cancer type cohort consists of a different proportion of 38x and 60x sequenced samples. **(b)** To reveal the sensitivity loss across all cancer types for the SNV, DBS, INDEL and SV counts, we performed a bootstrap approach. Here, we randomly assigned 38x and 60x samples in line with each proportion bin and computed the median fold change difference. We performed this procedure 100 times and calculated the average (±StDev) of the fold changes across all bootstraps. Subsequently, the obtained fold change scores were fitted using a linear regression model that allowed us to predict the sensitivity loss for every 38x/60x proportion. The sensitivity loss for every cancer type of every mutation type is depicted by coloured dots.

As expected, the fold change is negligible for SBS (ranging from -0.007X to 0.005X), DBS (ranging from -0.01X to 0.04X) and indels (ranging from -0.04X to 0.007X) for diffuse b-cell lymphoma (DLBCL, 92% of samples sequenced at 38x) and esophageal cancer (ESCA, fully consisting of 60x sequenced samples), respectively (Suppl. Fig. 3b). The SVs comprise the largest impact on sequencing depth with a fold change ranging between 0.13X for DLBCL and 0.07X for ESCA.

However, these lower SV detection sensitivities only marginally explain the observed increases in SV burden in the metastatic Hartwig tumors as compared to the primary PCAWG tumors. In fact, the SV burden statistics remained unchanged after applying the tumor type-specific correction factors (see Suppl. Fig. 4b). Among the 13 significant cancer types with increased SV burden, only esophageal and pancreas carcinomas and diffuse large B-cell lymphoma lost significance after applying this correction factor. In fact, in DLBCL the fold change almost did not change after correction (fold change of x2.2 raw versus x1.9 adjusted) and the loss of significance is more likely related to the high variation in SV burden between samples in the primary and metastatic cancer cohort as well as by limited sample size.
Finally, the mutation burden for SNV, DBS and INDEL are almost identical between the raw and the adjusted burden (Suppl. Fig. 4a). Consequently, the observed fold changes between primary and metastatic are highly consistent between raw and adjusted mutation counts. Overall, this downsampling analysis demonstrates that, after minimal adjustments, the lower sequencing coverage of PCAWG samples marginally impacts the mutation calling sensitivity for all mutation types.

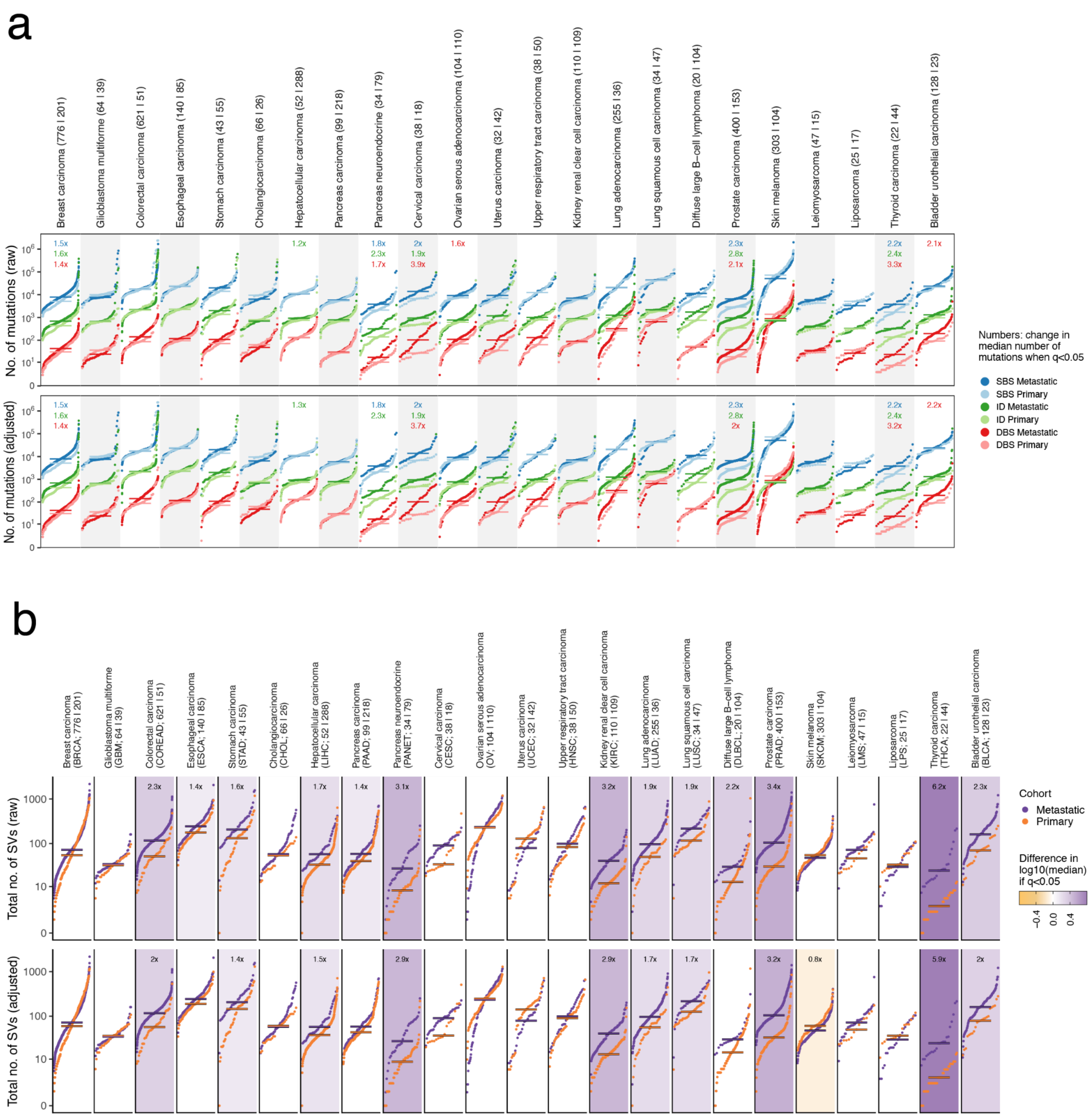

Supplementary Figure 4: Effect of sequencing depth on mutation burden. Cumulative distribution function plot (samples were ranked independently for each variant type) of tumor mutation burden for each cancer type for SMNVs **(a)** and SVs **(b)**. The SMNV plot **(a)** shows on top the cumulative distribution function plot for each tumor type for SBS (blue), INDELs (green) and DBS (red). Below, the cumulative mutation distributions adjusted for the estimated sensitivity loss per cancer type caused by differences in 38x/60x sequencing depth proportion. Similarly, on top of the SV plot **(b)** the cumulative distribution function plot for each tumor type for the SV mutation burden and below the cumulative mutation distributions adjusted for the estimated sensitivity loss per cancer type caused by differences in 38x/60x sequencing depth proportion. Horizontal lines represent median values. Fold change labels are included only when two-sided Mann-Whittney adjusted p-value < 0.01.

The impact of clonality on variant calling

We found that metastatic lesions generally present lower intra-tumor heterogeneity compared to primary tumors as a result of the severe evolutionary constraints imposed by anti-cancer therapies and/or the metastatic seeding event. The clonal outgrowth of subclonal mutations prior to the evolutionary process will become clonal (i.e., “clonality illusion”) and more easily detectable by bulk WGS, which may in part explain the increased TMB and SV burden in metastatic samples of some cancer types. To assess the impact of clonality on SNMV and SV mutation burden, we compared primary and metastatic samples independent of clonality. For this, we computed the average proportion of clonal mutations by dividing the number of clonal mutations by the total mutation burden (i.e. we relied on the same clonality score that we used in main Figure 1 of the manuscript, see methods). We selected samples with high global clonality (defined as those with a fraction of >85% clonal mutations) across primary and metastatic tumors, and created sub-cohorts by cancer type populated by these samples. The cohort size of each primary and metastatic cancer type is depicted at the top of suppl. Figure 5. This approach resulted in a sufficient number of representatives to compare both stages of tumor development independent of clonality.

As depicted in Supplementary Figure 5 (see below), the fold change differences for SBS, DBS and indel mutation burdens of clonal primary and metastatic samples (Suppl. Fig. 5) were very much in line with the mutation burdens not filtered by clonality (Fig. 2). This was most obvious for breast, cervix and prostate carcinoma where we noticed highly similar fold change rates for all mutation types. Thyroid and pancreas neuroendocrine cancers lost their significant increase, but this effect can be attributed to the reduction of the sample size. Indeed, the SBS, DBS and indel fold change metastatic increase was 1.2x, 1.2x and 1.1x, respectively, for clonal pancreas carcinoma which is in good agreement with the cohort not filtered on clonality (i.e., 1.8x, 2.3x and 1.7x respectively - Fig. 2). Similarly, clonal thyroid cancers showed a fold change increase of 1.9x, 2.2x and 2.6x, respectively, whereas we found a very similar increase of 2.2x, 2.4x and 3.3x for the unfiltered thyroid cohort (main Figure 2). Performing mutational signature analysis on the clonal mutations revealed a contribution landscape that showed a strong resemblance to the mutations contributions of the unfiltered samples (Suppl. Fig. 5, bottom panels).

Similar conclusions can be drawn from the SV comparison. After subsetting for clonal samples, we found that 6 metastatic cancer types remained their significant impact on SV burden increase (Suppl. Fig. 6). As with SNMVs, the reduced sample size explained the loss of significance in the 7 cancer types which encompassed a significant metastatic SV burden increase in the unfiltered cohort. This is shown by the highly similar fold change differences between clonal and unfiltered cancer cohorts: esophageal carcinoma (1.2x- clonal versus 1.4x - unfiltered), pancreas carcinoma (1.3x vs 1.4x), lung adenocarcinoma (1.4x vs 1.9) carcinoma. Only clonal metastatic pancreas neuroendocrine and thyroid carcinoma cancers showed a slightly lower SV burden compared to their metastatic counterpart samples not filtered by clonality: pancreas neuroendocrine (2.1x- clonal versus 3.1x - unfiltered) and thyroid carcinoma (3.9x vs 6.2x).

In conclusion, this analysis demonstrates that cancer samples with a comparable fraction of clonal mutations show highly similar SNMV and SV mutation profiles to those cancer samples not filtered on clonality. Therefore, the clonal selection/expansion imposed by the evolutionary bottlenecks seems to only marginally contribute to the increased SNMV and SV mutation burdens in metastatic cancers as described in Figure 2 and Figure 3.

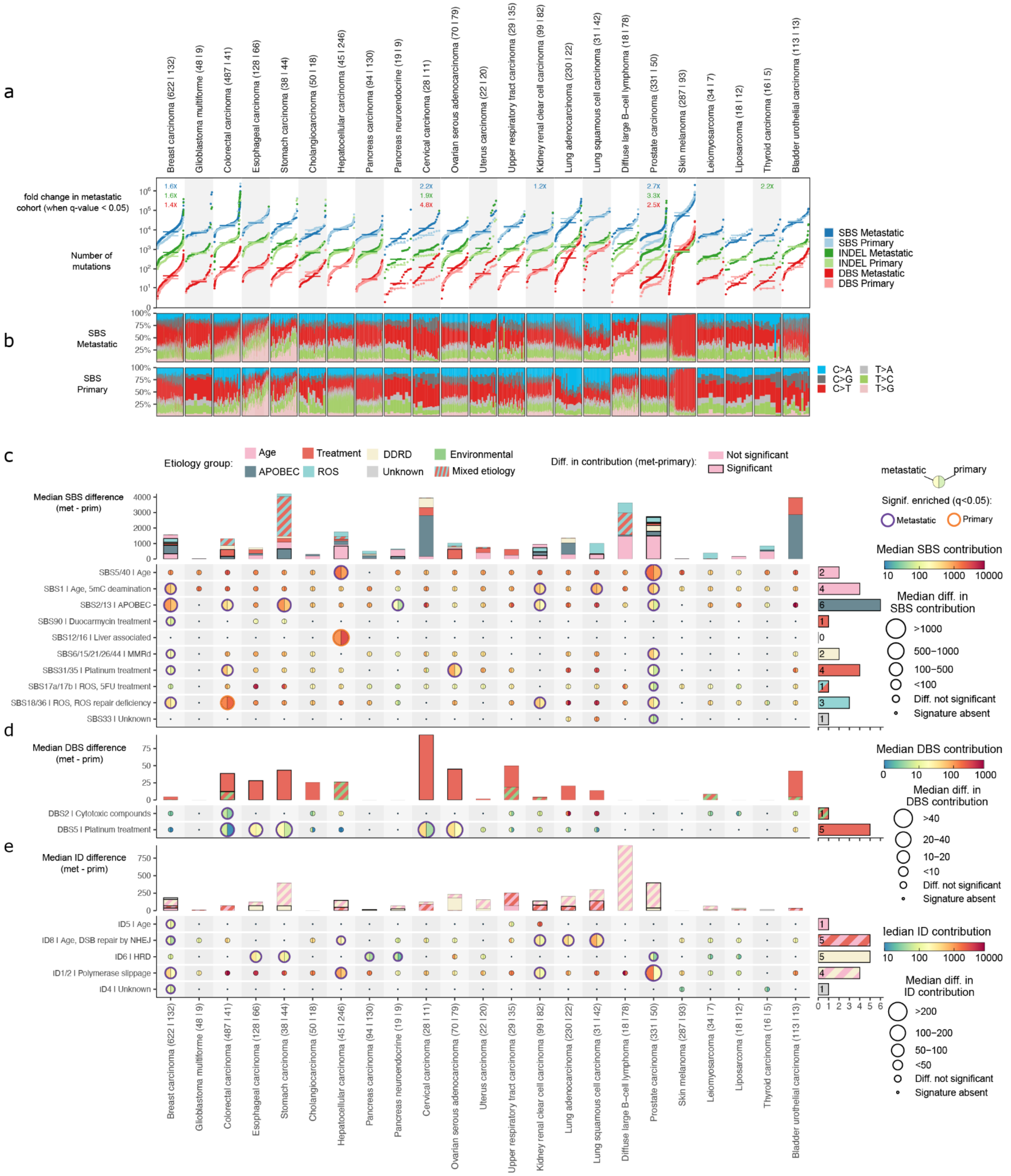

Supplementary Figure 5: Effect of clonality on passenger SMNV mutation landscape. Each cancer type cohort was subsetted with samples having a clonality of at least 0.85. The SMNV plot shows on top **a)** the cumulative distribution function plot for each tumor type for SBS (blue), INDELs (green) and DBS (red). Panel **b)** represents the SBS mutational spectra of metastatic (top) and primary (bottom) patients. Below, the mutational signature contribution landscape for samples with a clonality estimate of at least 0.85 for **c)** SBS, **d)** DBS and **e)** indel mutations. Horizontal lines represent median values. Fold change labels are included only when two-sided Mann-Whittney adjusted p-value < 0.05.

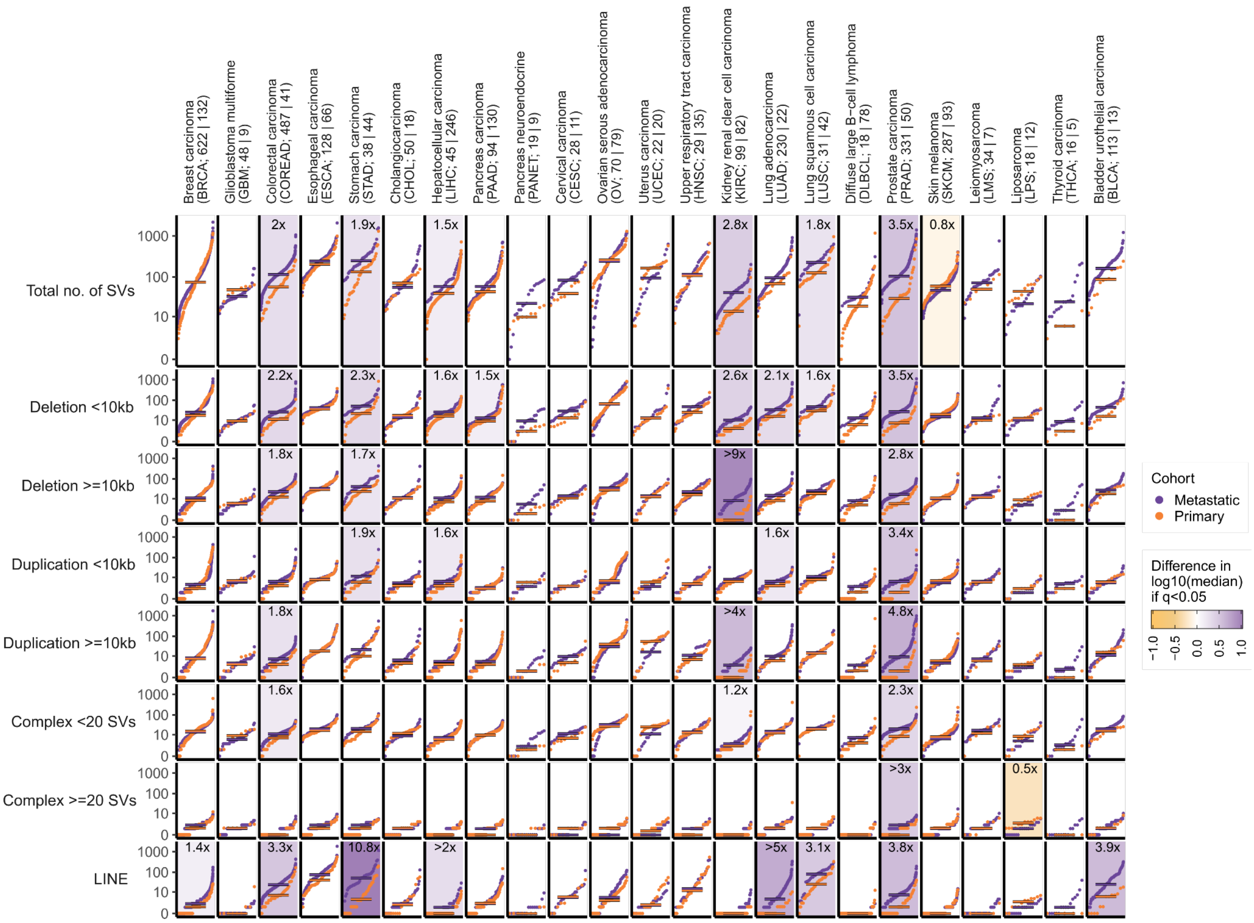

Supplementary Figure 6: Effect of clonality on passenger structural variant landscape. Each cancer type cohort was subsetted with samples having a clonality of at least 0.85. The cumulative distribution function plot for each tumor type for the SV mutation burden for samples with a clonality of at least 0.85. The cumulative distribution plots are stratified by SV type. Horizontal lines represent median values. Fold change labels are included only when two-sided Mann-Whittney adjusted p-value < 0.05.

The impact of pipeline differences on variant calling

Next, we compared the genomic features obtained with the Hartwig pipeline to the consensus somatic calls that were generated by the PCAWG Working Groups available on the PCAWG resource page. Many mutational features are closely connected with correct sample purity determination and ploidy level of the sample. We therefore assessed the WGS-based purity estimates between PCAWG consensus calls and the ones obtained from the Hartwig pipeline. We found that for 87% of the whitelisted samples of the ICGC part (n=1,916) the purity estimates were within the 10% range difference between the two pipelines (Suppl. Fig. 6). Only 12 samples (0.6% of the samples) showed a remarkable difference where Hartwig estimated the purity close to 100% whereas PCAWG reports a purity level lower than 50%. Although improvements on purity estimations have been made with the latest version of purple, the low number of discrepant

samples can be explained by the low levels of aneuploidy for these samples. For the ploidy levels, we observed that 90.0% of the samples showed a similar ploidy estimate (i.e. within a 10% range difference of ploidy level) between the two pipelines (Suppl. Fig. 7). The remaining samples show either a doubling (4.2%) or a halving (3.4%) of the ploidy scores. Finally, 93.4% of the samples shared the same whole-genome duplication status.

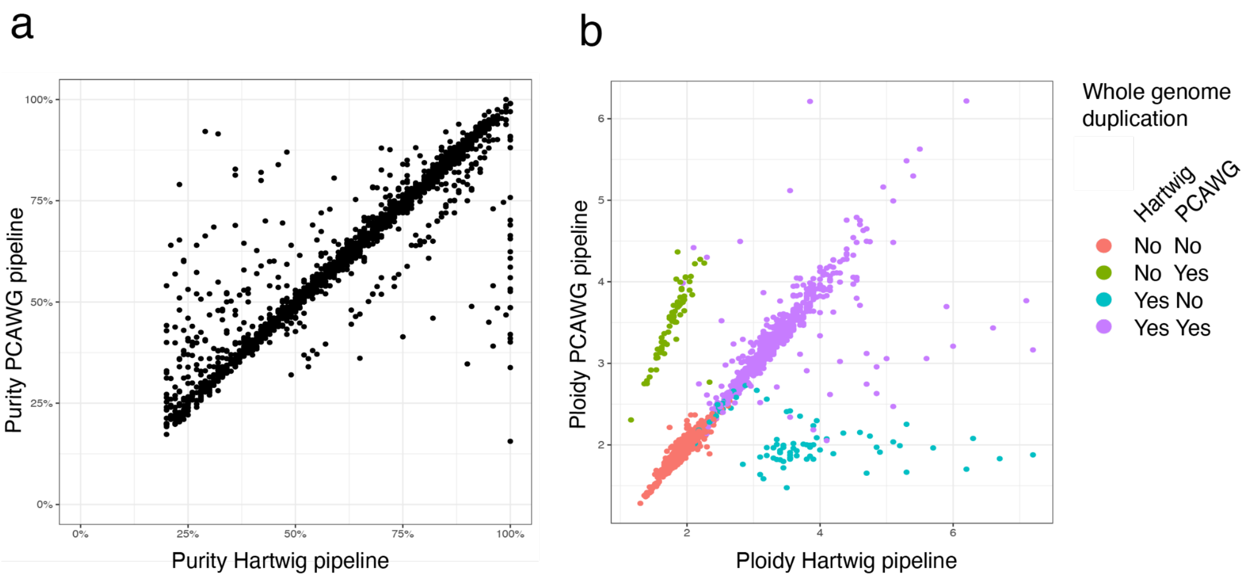

Supplementary Figure 7: Effect of mutation calling pipeline differences for purity and ploidy scores. Comparison of all whitelisted ICGC-PCAWG samples for purity **(a)** and ploidy **(b)** obtained by the Hartwig pipeline to the scores provided by PCAWG resource page. The purity estimates were within the 10% range difference between the two pipelines for 87% of the samples. 90.0% of the samples showed a ploidy estimate within the 10% range difference and most of the remaining samples show either a doubling (4.2%) or a halving (3.4%) of the ploidy scores and explain most of the whole genome duplication estimates.

To compare the mutational burden for each mutation type between PCAWG consensus callset and mutations obtained with Hartwig pipeline, we excluded the trinucleotide MNVs as well as SV calls with less than 500 base pairs in length because these are not called by the PCAWG pipeline. The figure below shows that both pipelines share similar calling sensitivity for SBS as shown by the median fold change of 0.08x (Suppl. Fig. 8). The DBS (fold change of 0.36x) and indels (fold change of 0.34x) are more sensitively called by the Hartwig pipeline. SAGE includes the local phasing information for DBS and indel mutations (co-existence on the same read) and integrates a context-specific scoring system for indels that is particularly applied to indels in repeat contact. These SAGE features may explain the higher calling sensitivity for DBS and indel mutation types. We also observed that the mutation spectra were overall in good agreement (i.e. cosine similarity > 0.90 for 96%, 80% and 94% for SNV, DBS and indel respectively) between both pipelines (Suppl. Fig. 8). As expected, the largest discrepancies were observed for samples with low mutation burden.

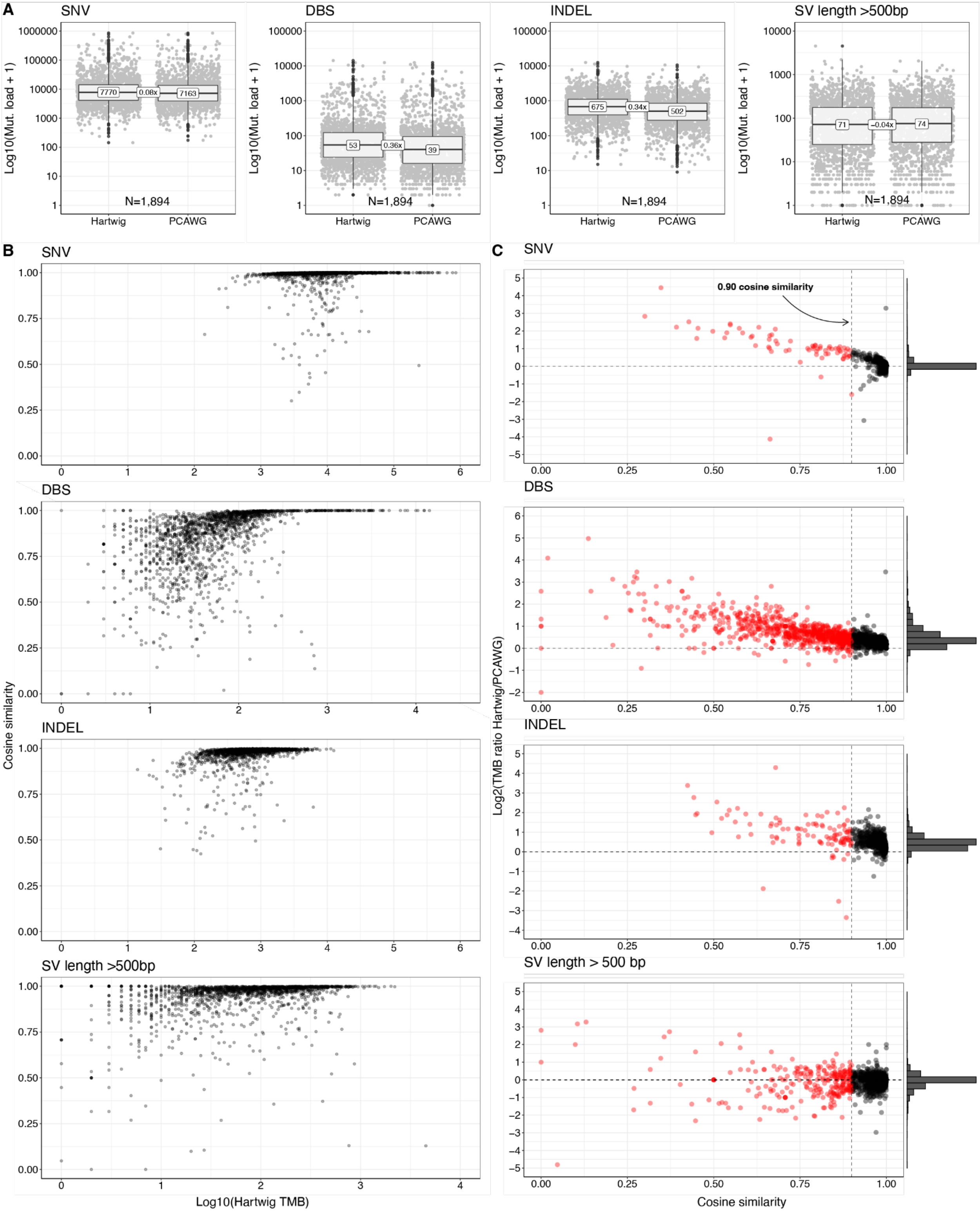

Supplementary Figure 8: Effect of mutation calling pipeline differences for somatic mutations. (a) Comparison of all whitelisted MSS PCAWG samples (N=1,894) for SNV, DBS, INDEL and SV counts obtained by the Hartwig pipeline to the mutation counts provided by PCAWG resource page. The PCAWG consensus calls show a small difference in sensitivity for DBSs, indels, and nearly the same sensitivity for

SNVs and SVs. The fold changes of all mutation types for the two sequencing depth modes are depicted in table 1. Box-plots: center line, median; box limits, first and third quartiles; whiskers, lowest/highest data points at first quartile minus/plus 1.5× IQR. **(b)** Most of the samples harbor a highly similar mutation context (cos sim > 0.90). **(c)** Only samples with a very low mutation burden show a different mutation context. N=number of whitelisted PCAWG samples.

A comparison in driver landscape between Hartwig and PCAWG pipeline is more challenging because the Hartwig and PCAWG driver analysis rely on different driver detection tools and driver reference databases. Nevertheless, we observed that 90% of the samples showed a similar number of driver hits per sample between PCAWG driver consensus callset (only ICGC part; n=1613) and drivers obtained with the Hartwig pipeline. 32% of the samples showed the same number of driver genes per sample, while 41% and 17% of the samples showed a difference of respectively 1 and 2 in driver hits per sample. On gene basis per sample, we found that ~50% of the 4738 driver genes reported by either the Hartwig or PCAWG pipeline were found by both pipelines, while ~34% were Hartwig-unique and ~16% PCAWG unique. This may be explained by the inclusion of cancer driver databases in the Hartwig driver analysis, while the PCAWG driver catalog was established with a compendium of *de novo* driver discovery tools. Indeed, a higher similarity in gene drivers was found for the top-20 mutated genes with 68% overlap in gene driver landscape between PCAWG and Hartwig pipeline and ~25% Hartwig-unique and 7% PCAWG-unique. Taken together, the PCAWG mutation callset generated by Hartwig analytical pipeline is in line with the PCAWG consensus mutation callset. The ICGC part of the Hartwig processed PCAWG dataset is made available as a WGS cancer genomic resource at the PCAWG release page (https://dcc.icgc.org/releases/PCAWG/Hartwig).

Supplementary Note 2

Introduction 23

Primary and metastatic comparison of genomic features by breast, colon and uterus carcinoma subtypes 24

- Dataset annotation 24
- Tumor clonality 25
- Karyotype and genomic instability indicators 26
- Tumor mutation burden and mutational signatures 29
- Structural variant burden 31
- Drivers 33
- Conclusion 35

Primary and metastatic comparison of genomic features by primary clinical progression status 36

- Dataset annotation 36
- Tumor clonality 36
- Karyotype and genomic instability indicators 37
- Tumor mutation burden and mutational signatures 39
- Structural variant burden 43
- Drivers 45
- Conclusion 48

Primary and metastatic comparison of genomic features by metastatic biopsy location 48

- Dataset annotation 48
- Tumor clonality 49
- Karyotype and genomic instability indicators 49
- Tumor mutation burden and mutational signatures 54
- Structural variant burden 59
- Drivers 63
- Conclusion 70

Introduction

The original goal of our study was to identify and describe pan-cancer genomic differences between primary and metastatic tumors. In this supplementary note we aim to rule out -or explain- potential biases introduced by the virtue of cohorts heterogeneity. Specifically, we will assess the impact of cancer subtypes, differences in primary patient prognosis and metastatic biopsy location.

Thus, it is not our main goal to perform an exhaustive *de-novo* analysis across cancer subtypes, metastatic locations or according to primary clinical progression status. We believe dedicated analyses are better suited to understand all these facets of tumor evolution.

Primary and metastatic comparison of genomic features by breast, colon and uterus carcinoma subtypes

Dataset annotation

To rule out the possibility that certain observed discrepancies between primary and metastatic are driven by a heterogeneous representation of cancer subtypes across the two cohorts, we aimed to classify patients according to their clinical subtype. Unfortunately, a large fraction of the patients included in our primary and metastatic do not have a full clinical annotation that would be required to extend this analysis to all cancer types.

Among the cancer types included in our study, we were able to provide cancer subtype annotation for three cancer types: breast cancer, colorectal and uterus carcinomas. We thus only included in this analysis patients with clinically annotated cancer subtype.

Breast cancer

From the Hartwig dataset we used internal clinical data to annotate the molecular subtype: ER+/HER2-, ER-/HER2+, ER+/HER2+ and triple negative breast cancer (TNBC). All Hartwig breast samples except for 41/776 were properly classified into these groups (see Supp. Table 1). For the PCAWG dataset, as it is formed by multiple cohorts sequenced by different institutions, we gathered the clinical annotation from their respective source:

- BRCA-EU cohort (https://dcc.icgc.org/projects/BRCA-EU). All patients were annotated as ER+/HER2-.
- BRCA-UK (https://dcc.icgc.org/projects/BRCA-UK). All patients were annotated as triple negative.
- BRCA-US. This cohort includes patients with different molecular subtypes. Annotation of the specific subtype was performed using clinical data from TCGA (downloaded from cbioportal and from https://gdc.cancer.gov/about-data/publications/pancanatlas).

After annotation the four aforementioned breast cancer subtypes included sufficient number of patients (N>=5) in both primary and metastatic tumors to perform a tumor subtype comparison (see Supp. Table 1 and the summary image below).

Colorectal and uterus cancers

We divided colorectal and uterus cancer patients into microsatellite stable (MSS) and microsatellite insatiable and/or POLE mutants (MSI/POLE). MSI annotation was gathered using our metadata (Supp. Table 1) and POLE status was annotated using the sample specific driver alterations (i.e., a tumor was annotated as POLE mutant if it harbored a non-synonymous driver mutation in POLE).

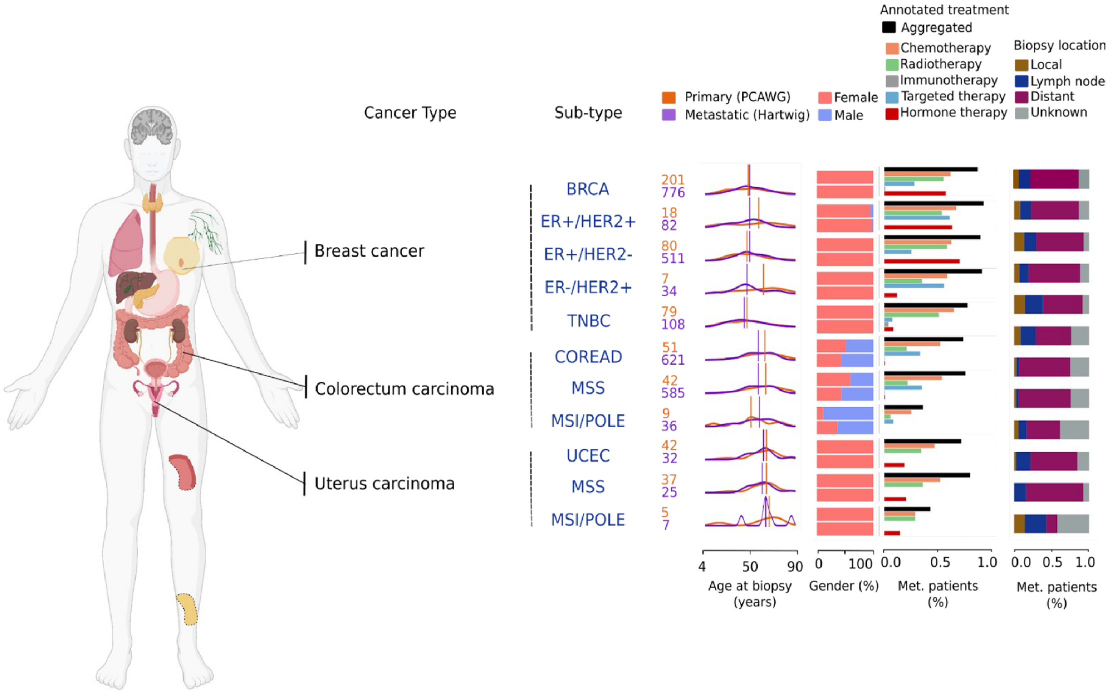

Supplementary Figure 9: Depiction of number of primary (PCAWG) and metastatic (Hartwig) samples, age at biopsy, type of treatment (in metastatic cohort) and metastatic biopsy location per cancer subtype.

Tumor clonality

We first evaluated cancer subtype differences in tumor clonality. For the majority of cancer subtypes, we observed an increase in tumor clonality in metastatic tumors compared to primaries, which is in agreement with the results based on the aggregated cancer types (also included in the figure, see below Suppl. Fig. 10 and Fig. 1b). However, our comparison also revealed different patterns across cancer subtypes that are worth discussing. First, breast carcinoma subtypes showed a remarkable similarity in tumor clonality in both primary and metastatic tumors with a modest increase in tumor clonality (i.e., lower intra tumor heterogeneity) in metastatic tumors. Second, both uterus and colorectal MSI/POLE primary tumors showed higher intra tumor heterogeneity (i.e., lower tumor clonality) compared to metastatic uterus and colorectal MSI/POLE patients, respectively. However, the tumor clonality of MSS colorectal and uterus were relatively more similar between the two stages.

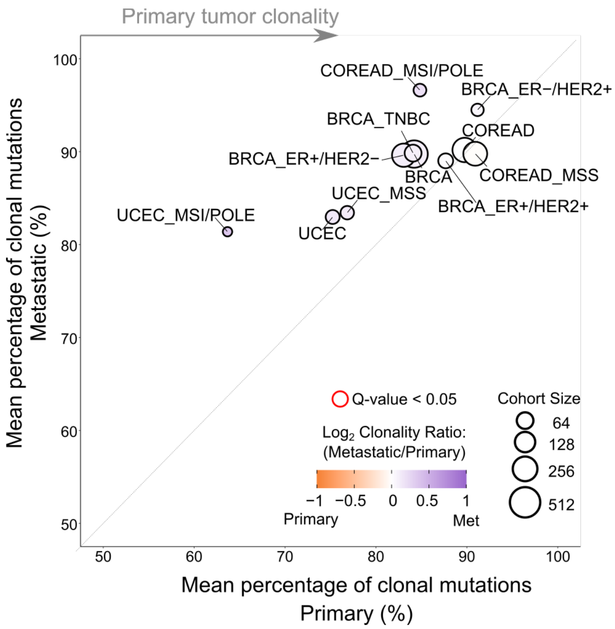

Supplementary Figure 10: Mean percentage of clonal mutations in primary (x-axis) and metastatic (y-axis) tumors across cancer subtypes. Dots are coloured according to Log_2 of the clonality ratio (metastatic divided by primary). Size of dots are proportional to the total number of samples (primary and metastatic). Red edge lines represent a two-sided Mann-Whitney adjusted p-value < 0.05.

Karyotype and genomic instability indicators

We first analyzed whether cancer subtypes may have an influence in the karyotype compared to grouping together all patients by the general cancer type.

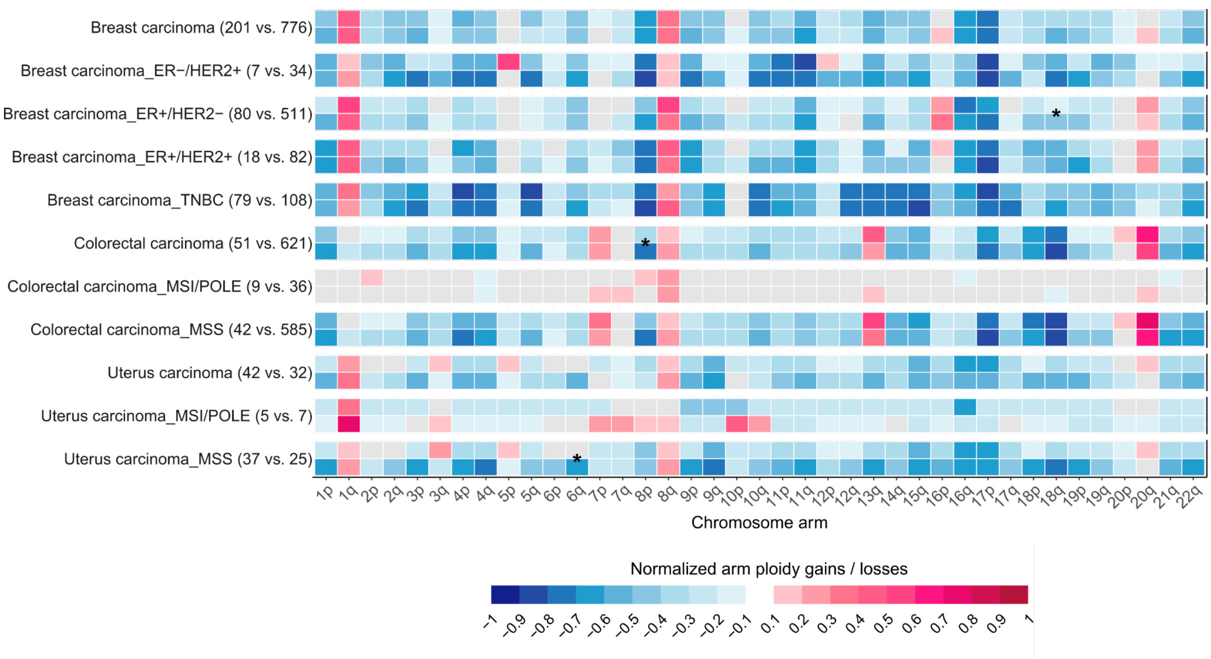

Supplementary Figure 11: Tumor karyotype. Heatmap representing the normalized mean chromosome arm ploidy gains and losses relative to the expected 2n ploidy status in primary (top) and metastatic (bottom) tumors from cancer subtypes. "*", significantly different mean distributions between primary and metastatic tumors (two-sided Mann-Whitney adjusted p-value < 0.01).

Breast cancer subtypes showed a relatively conserved karyotype between primary and metastatic tumors, which was also the general trend observed in the aggregated cancer type (Suppl Fig. 11 top row). Colorectal and uterus carcinomas MSS tumors also showed a relatively conserved karyotype between primary and metastatic tumors. The karyotype landscape of MSI/POLE colorectal and uterus carcinomas tumors was essentially different compared to their MSS counterparts. Finally, no significant differences were observed between primary and metastatic MSI/POLE subgroups, although the sample size for these subtypes was very low.

We next compared the tumor genomic instability indicators:

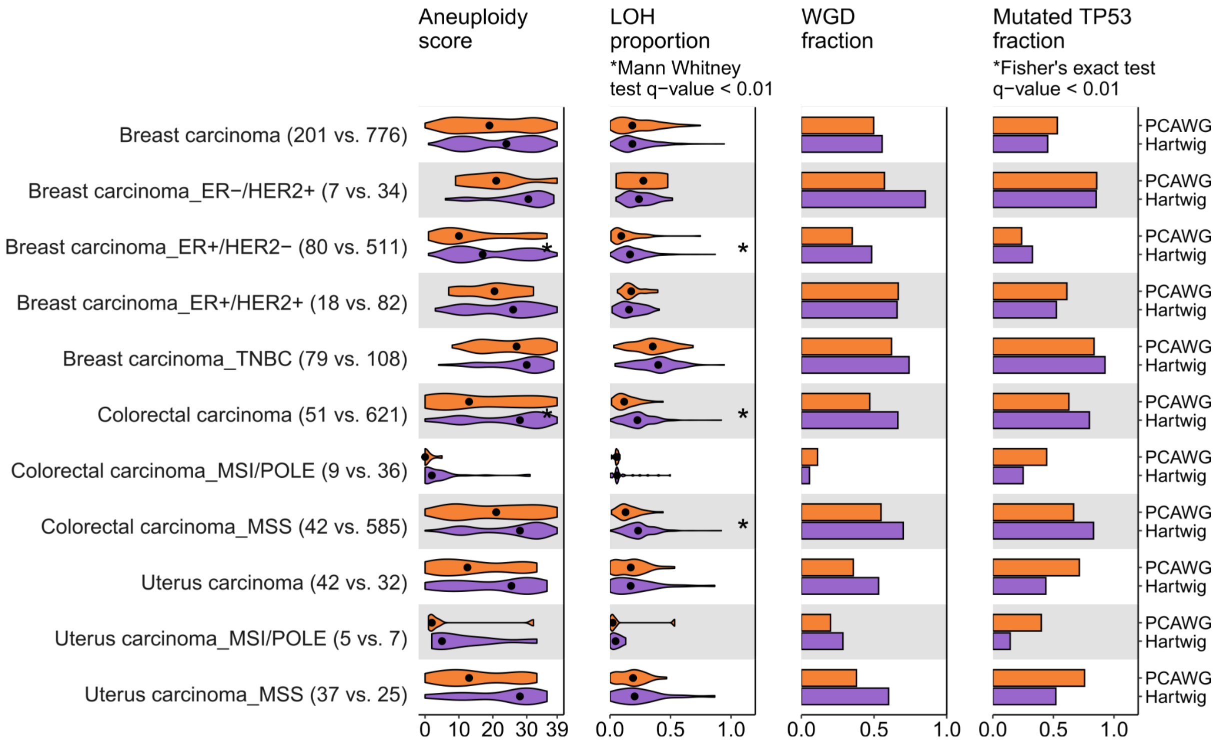

Supplementary Figure 12: Comparison of four genomic indicators between primary (top) and metastatic (bottom) tumors across cancer subtypes. From left to right, aneuploidy score from ref[9], proportion of genome undergoing LOH, fraction of samples bearing whole genome duplication (WGD) and *TP53* alterations. Black dots represent the median values. "*", two-sided Fisher’s exact test adjusted p-value < 0.01 for discrete features (WGD and *TP53*) and two-sided Mann-Whitney adjusted p-value < 0.01 for the continuous features.

The aggregated breast carcinoma displayed a modest and non-significant aneuploidy score increase in metastatic tumors (Suppl. Fig. 12). This pattern was mostly consistent across the four screened subtypes, although ER+/HER2- patients, which globally showed the lowest aneuploidy score across all breast cancer subtypes, displayed a significant increase in metastatic tumors. Of note, higher aneuploidy scores in this subtype (ER+/HER2-) may be in turn associated with higher WGD metastatic rates, as reported for lung and colorectal adenocarcinomas in the main text (see Fig.1e).

The reported increase in genomic instability indicators in metastatic colorectal carcinoma tumors (i.e., aneuploidy score, LOH proportion, WGD rates) was primarily associated with the MSS subtype, whereas the MSI/POLE subtype comparison did not reveal any obvious difference between the two stages. Similarly, the pattern of genomic instability indicators in the aggregated uterus carcinoma subtypes were generally associated with the MSS subgroup, given the dominant representation of this subtype in both primary and metastatic stages.

All things considered, our analysis revealed that grouping by cancer subtypes generally rendered consistent results that using the aggregated cancer types.

Tumor mutation burden and mutational signatures

We next compared the tumor mutation burden (i.e., SNVs, IDs and DBSs mutation burden) between metastatic and primary tumors subtypes (see Suppl. Fig. 13). Overall, the cancer subtypes showed a consistent tendency compared with the main cancer types (see Fig. 2a). However, there are some important differences that are worth commenting on.

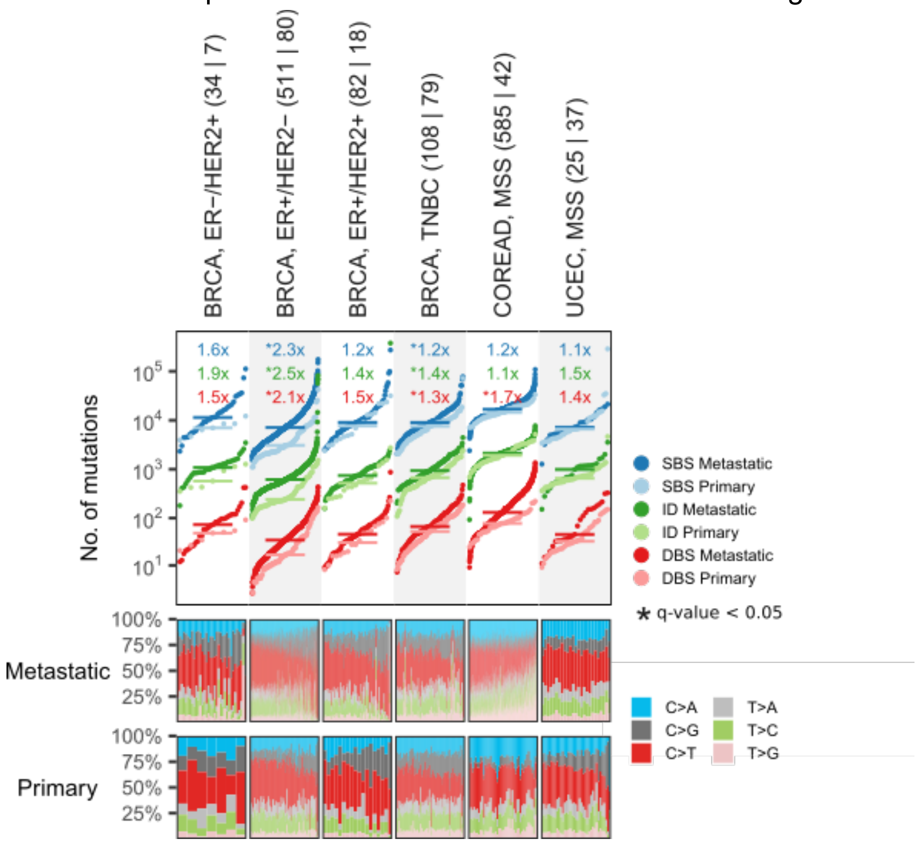

Supplementary Figure 13: Top panel, cumulative distribution function plot (samples were ranked independently for each variant type) of tumor mutation burden for each cancer subtype for SBS (blue), IDs (green) and DBS (red). Horizontal lines represent median values. “*”, two-tailed Mann-Whitney q-value < 0.05. Bottom panel, SBS mutational spectra of metastatic (top) and primary (bottom) tumor patients. Patients are ordered according to their TMB burden.

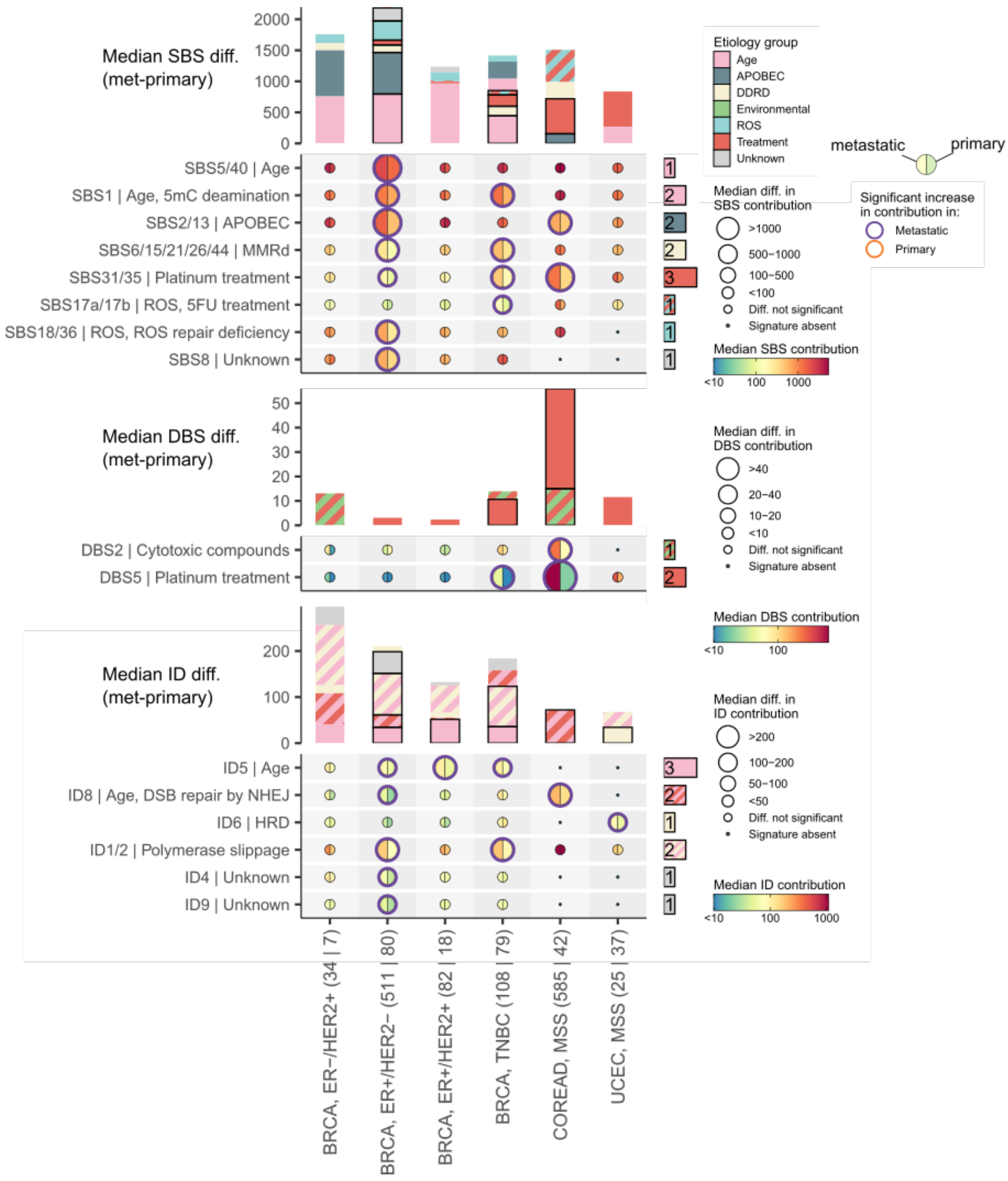

Supplementary Figure 14: Top panel, moon plot representing the mutational burden differences attributed to each mutational signature in metastatic (left) and primary (right) tumors from cancer subtypes. Edge thickness and colors represent significant differences (two-sided Mann-Whitney q<0.05, ±1.4x fold change) and the direction of the enrichment, respectively. The size of circles are proportionate to the mutation burden difference. Right bars, number of metastatic cancer types with a mutational signature significant enrichment. Top stacked bars represent the cumulative signature exposure difference. Thicker bar edge lines represent significance. Bars are coloured according to the annotated etiology. Only mutational signatures with known etiology or with at least one cancer subtype with significant metastatic enrichment

are included. Mid and bottom panels equivalent for DBS and IDs, respectively. Diff., difference. Muts. mutations. Sig., mutational signature. Mut. mutational. Susp., suspected. Def., deficiency.

First, in spite of the fact that in all breast cancer subtypes there was an increase in tumor mutation in metastatic tumors, the magnitude of this increase was substantially different. ER+/HER2- showed a strong TMB increase (x2.3 SNVs, x2.5 IDs and x2.1 DBs; q-value < 0.05) whereas ER+/HER2+ and TNBC showed a moderate metastatic increase (see above). Interestingly, consistent with our observations across the main cancer types, the fold change of IDs and DBs was generally higher across subtypes than the SNV fold change (probably because of the higher contribution of treatment-associated alterations for these types of mutations see below). In fact, when looking at mutational signature exposure differences, we observed an overall shared etiology of the mutational processes showing an increased exposure at the metastatic setting (top bars of the mutational signature panel, Suppl. Fig. 6 above). The main exception was the lack of APOBEC enrichment in ER+/HER2+ metastatic tumors and the very modest enrichment of platinum treatment mutations in both HER2+ metastatic tumors compared to ER+/HER2- and TNBC, which are likely associated with the treatment regimes that these patients underwent. Moreover, in agreement with the TMB increase, ER+/HER2- showest an extensive metastatic increase across most of the operative mutational processes, including clock-like mutational processes (SBS1 and SBS5/SBS40), APOBEC (SBS2/13) and treatment associated (e.g., SBS31/SBS35), among others. Finally, TNBC also showed a strong increase in SBS1 and mutational processes associated with platinum treatment (SBS31/35 and DBS5). In summary, the increased TMB observed in metastatic breast cancer tumors was to some extent consistent across subtypes. However, our data also showed that the magnitude of differences are subtype specific and that ER+/HER2- show the strongest discrepancies between primary and metastatic tumors, whereas ER+/HER2+ tumors were the most relatively similar among all the analyzed subtypes.

MSS colorectal and uterus carcinomas showed comparable SNVs and IDs burden, which is consistent with the grouped cancer type patterns (see Fig. 2a, Suppl. Fig. 13 and 14). However, colorectal and uterus MSS metastatic tumors also showed a moderate increase in DBS burden (x1.7 in colorectal and x1.4 in uterus). These DBSs were primarily attributed to exposure to platinum cherapies, which led to a substantial DBS burden increase in (+50 median DBS per sample) in metastatic tumors compared to primary patients.

Structural variant burden

We then compare the structural variant (SV) burden, which encompasses structural alterations different from point mutations and indels such as deletions (short <10kb and long >=10kb), duplications (short <10kb and long >=10kb), complex events and LINE insertions. For every cancer subtype, we then compared the differences in SV burden between primary and metastatic tumor patients, as we did in the analyses displayed in Figure 3a of the main text (see Suppl. Fig. 15).

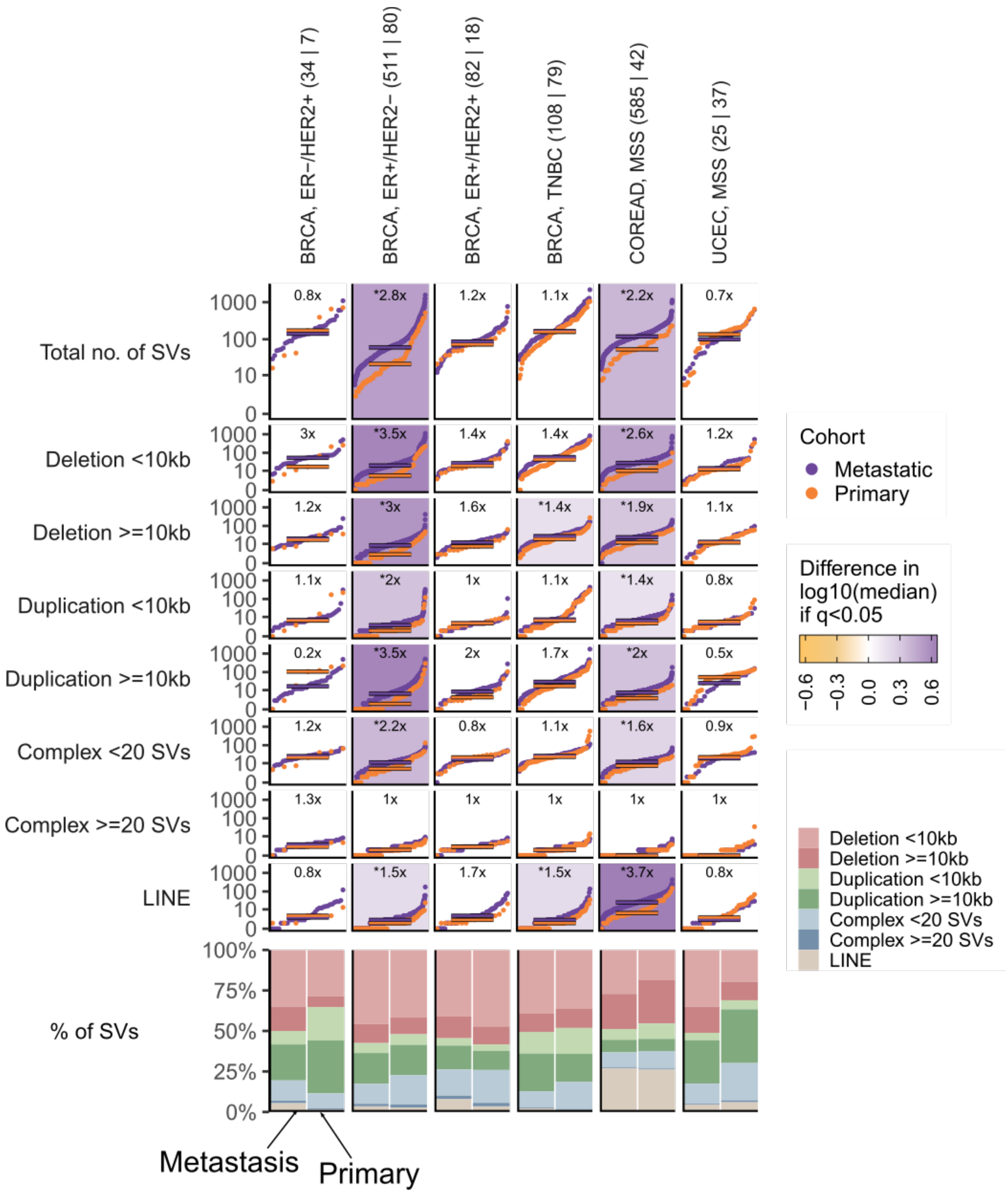

Supplementary Figure 15: S-plots, cumulative distribution function plot (samples ranked independently for each SV type) of tumor mutation burden for each cancer subtype for (from top to the bottom) the aggregated structural variant (SV) burden, small deletions (<10kb), large deletions (>=10kb), small duplications (<10kb), large duplications (>=10kb), complex events (<20 breakpoints), complex events (>=20 breakpoints) and LINEs insertions. Horizontal lines represent median values. Backgrounds are coloured according to the relative enrichment, defined as: $\log_{10}$(median SV type burden in metastatic tumors + 1) − $\log_{10}$(median SV type burden in primary tumors + 1). Coloured backgrounds and “+” are displayed

when two-sided Mann-Whitney comparison renders a significant q-value < 0.05. Fold change labels are displayed with '>' when the SV burden for primary tumors is 0 (see methods for more details). For each cancer subtype, bottom bar plots represent the relative fraction of each SV type in the metastatic (left) and primary (right) datasets.

First, our analysis revealed that the breast cancer ER+/HER2- group showed a substantial global SV burden increase in metastatic tumor compared to primary (x2.8 fold change, q-value <0.05). This increase was much higher than in other breast cancer subtypes that did not show a significant SV burden increase when aggregating all types of SVs. However, all breast cancer subtypes displayed an increase in short deletions and long deletions, and except for ER-/HER2+, in long duplications and LINE insertions too. Altogether, our results indicated that while the metastatic increase in deletions, long duplications and LINE insertions are generally subtype-independent, the aggregated global SV burden are mainly attributed to the ER+/HER2- subtype, which showest the highest global discrepancy on their structural variant landscape between primary and metastatic tumors.

MSS colorectal and uterine tumors showed a highly concordant SV burden pattern compared to the main cancer type groups from Fig. 3a. Specifically, MSS colorectal cancer showed a strong enrichment in almost all types of structural variants (except for complex events with more than 20 breakpoints). This result perfectly matches the differences from the global colorectal cancer cohort. Similarly, MSS uterus carcinoma metastatic tumors did not show any enrichment across all evaluated SV types, which is also consistent with the results from the cancer type level analysis (see Fig. 3a).

Drivers

We next explored differences in the number of driver alterations per sample across primary and metastatic tumors based on the cancer subtypes groups (Suppl. Fig. 16). Our data revealed that the metastatic increased observed in the global breast cancer type is, to some extent, conserved across all breast cancer subtypes (i.e., +1.18 in the combined breast cancer group, +1.06 in ER-/HER2+, +1.74 in ER+/HER-, +1.03 in ER+/HER2+ and +0.85 in TNBC). Next, the highly consistent driver landscape observed in the grouped colorectal cancer was driven by the comparable number of driver events per sample in primary and metastatic MSS colorectal cancer patients. Conversely, MSI/POLE colorectal cancer primary patients displayed a higher average number of drivers per sample compared to metastatic MSI/POLE metastatic tumor patients. Such increase may be reflective of higher intra-tumor heterogeneity in the primary tumors (see above tumor clonality), although results should be taken with caution due to the low sample size and the lack of statistical significance. Uterus carcinoma displayed a non-significant and comparable distribution of number of driver events per sample in both the MSI/POLE and the MSS groups.

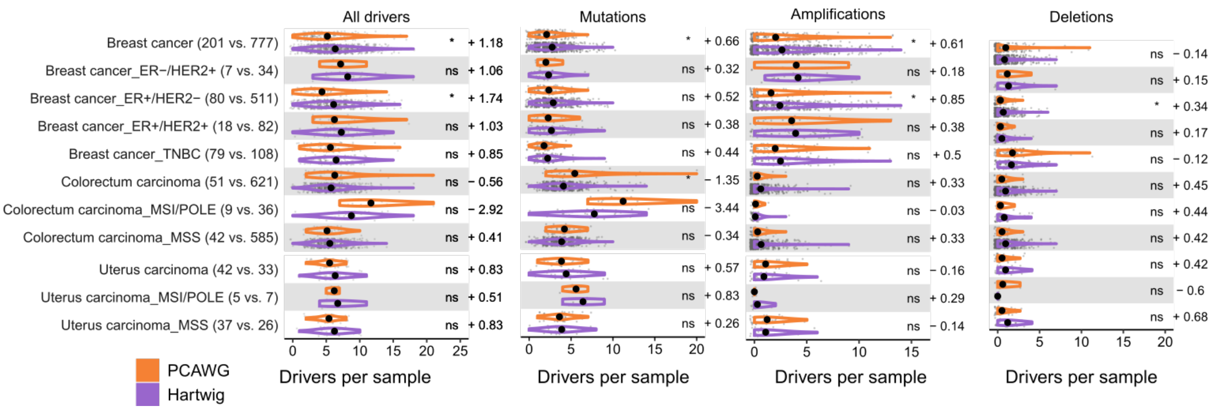

Supplementary Figure 16: Cancer subtype specific distribution of number of driver alterations, amplifications, deletions and mutations per patient in primary (top) and metastatic (bottom). Black dots represent the mean values. Labels display mean differences (metastatic - primary) in cancer subtypes with a significant difference. Ns, non-significant p-value. “*”, two-sided Mann-Whitney adjusted p-value < 0.01.

Then, for each cancer type specific driver gene that showed an enrichment in either tumor stage we analyzed the mutation prevalence discrepancies across cancer subtypes (Suppl. Fig. 17).

Primary (mutated / total samples)

Metastatic (mutated / total samples)

Cancer type	Gene	Cramer's V	P-value	P-value (FDR adjusted)
MUTATION				
Breast carcinoma (1/201 vs. 127/776)	ESR1	0.19	< 0.01	< 0.01
Breast carcinoma: ER-/HER2+ (0/7 vs. 0/34)	ESR1	0.00	n.s.	Not calc (min. freq < 5)
Breast carcinoma: ER+/HER2- (0/80 vs. 108/511)	ESR1	0.19	< 0.01	< 0.01
Breast carcinoma: ER+/HER2+ (0/18 vs. 8/82)	ESR1	0.14	n.s.	n.s.
Breast carcinoma: TNBC (1/79 vs. 1/108)	ESR1	-0.02	n.s.	Not calc (min. freq < 5)

Supplementary Figure 17: Cancer subtype specific comparison of driver alteration frequency between primary and metastatic tumors.

ESR1 was the one gene that rendered a significantly different frequency after correcting by FDR. Metastatic enrichment in ESR1 mutations were exclusively observed for ER+ subtypes (ER+/HER2- and ER+/HER2+). This is expected given that ESR1 mutations is a well-known mechanism of resistance to estrogen deprivation therapy in ER+ tumors. Finally, a slightly higher ESR1 mutation frequency was observed in metastatic ER+/HER2- (108/511, 21%) than in metastatic ER+/HER2+ (8/82, 9%).

Conclusion

In general terms, all differences reported in the main cancer types were consistently observed in at least one cancer subtype.

Moreover, we observed certain subtype specificities that are worth discussing. In breast carcinomas, the strongest genomic differences between primary and metastatic tumors were observed for ER+/HER2- patients followed by the TNBC subtype. Conversely, HER2+ tumors were more genomically similar between primary and metastatic tumors. Moreover, as expected, ER+ metastatic tumors were the subtypes showing an enrichment in ESR1 mutations. Finally, most of the differences observed in the aggregated colorectal and uterus cancers were attributed to the MSS subtype.

Primary and metastatic comparison of genomic features by primary clinical progression status

Dataset annotation

We aimed to annotate the patient's clinical progression status after the last follow up for primary tumors from five cancer types (i.e., breast, prostate, kidney renal clear cell, thyroid and pancreatic neuroendocrine). To do so, we gathered PCAWG donor’s clinical data from the ICGC data portal (https://dcc.icgc.org/, 22/08/2022). Patient’s progression status was partially available depending on the cancer type and the specific cohort. We grouped patients into two categories according to their clinical progression: progression/relapse (if their annotation included “progression” or “relapse”) and stable/remission (when their annotation included “stable” or “complete remission”). After the annotation, only prostate and pancreatic neuroendocrine tumors included a sufficient number of patients in the two categories (i.e., minimum number of 5 samples). Therefore the subsequent analyses were only performed for these two cancer types.

The following analyses will be performed by comparing each primary progression subgroup with all the metastatic tumors in a cancer type specific manner.

Tumor clonality

We first compared in a cancer type specific manner the mean fraction of clonal mutations of the two primary groups (i.e., progression/relapse and stable/remission) compared to metastatic tumors (Suppl. Fig. 18). Consistently with our observations in the main text (see Fig. 1b) we observed a significant increased clonality in metastatic tumors compared to both groups of primary across PRAD and PANET tumors. We thus do not observe any strong difference between the primary tumors with different progression status.

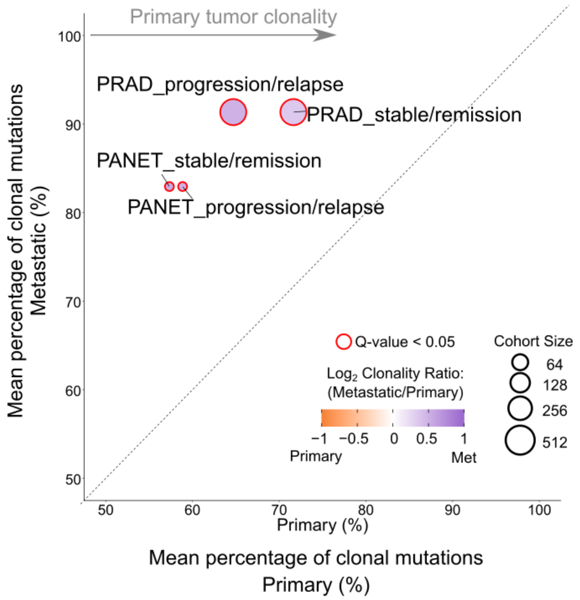

Supplementary Figure 18: Mean percentage of clonal mutations in primary (x-axis) and metastatic (y-axis) tumors. Primary tumors are split according to the patient's clinical progression status. Dots are coloured according to Log_2 of the clonality ratio (metastatic divided by primary). Size of dots are proportional to the total number of samples (primary and metastatic). Red edge lines represent a two-sided Mann-Whitney adjusted p-value < 0.05.

Karyotype and genomic instability indicators

We first compared the tumor karyotype splitting by the primary progression group (Suppl. Fig. 19). Overall, the observed differences between grouped primaries compared to metastatic tumors (first row of each cancer type) were maintained in the primary-progression subgroups in both cancer types. In pancreatic neuroendocrine tumors (PANET) both primary groups showed recurrent patterns of chromosome losses (RPCL), which were generally in agreement with the metastatic frequencies. In prostate cancer (PRAD), our analysis revealed that, in general, metastatic tumors showed higher prevalence of chromosome losses and chromosome 8q arm gains. Importantly, the differences in statistical significance of the comparison were likely the result of the differences in sample sizes across the two primary subgroups (12 patients compared to 33), because the direction and the magnitude of the observed karyotypic changes were generally in agreement (see Supp. Table 1 and Supp. Table 2).

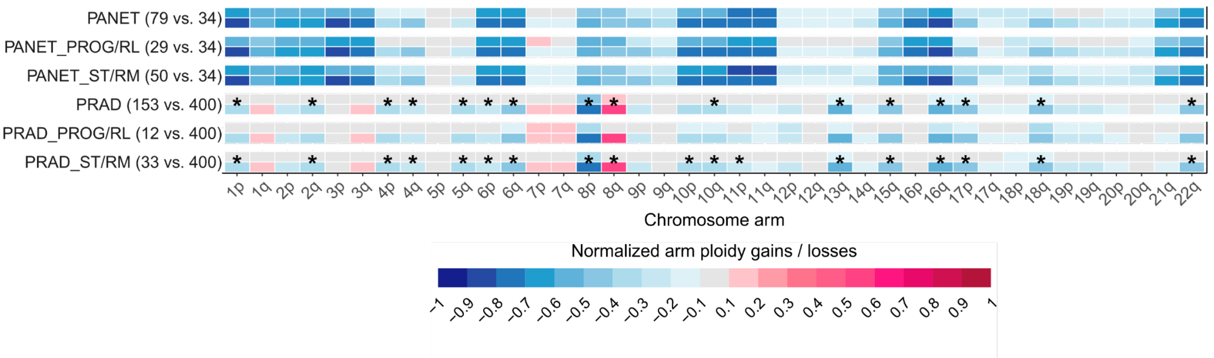

Supplementary Figure 19: Tumor karyotype. Heatmap representing the normalized mean chromosome arm ploidy gains and losses relative to the expected 2n ploidy status in primary (top) and metastatic (bottom) tumors from cancer types. Primary tumors are split according to the patient’s clinical progression status. "*", significantly different mean distributions between primary and metastatic tumors (two-sided Mann-Whitney adjusted p-value < 0.01).

Next, we evaluated the differences in genomic instability indicators:

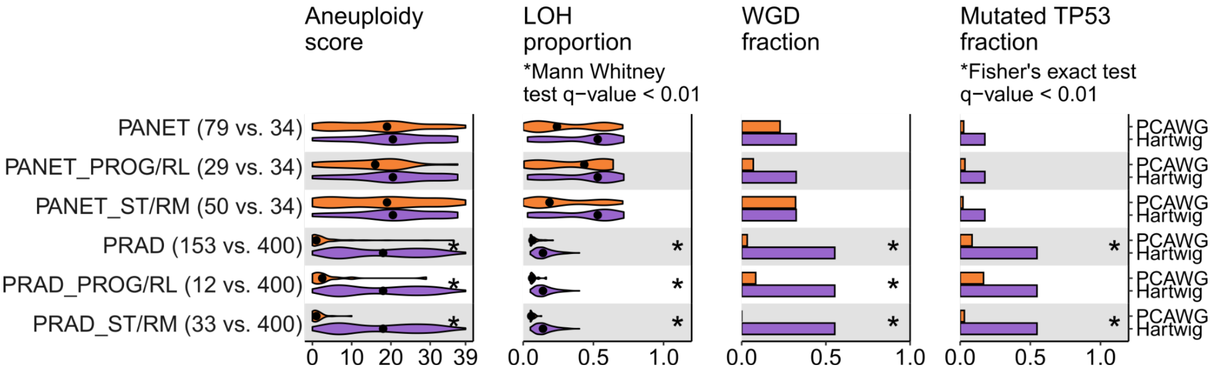

Supplementary Figure 20: Comparison of four genomic indicators between primary (top) and metastatic (bottom) tumors across cancer types. Primary tumors are split according to the patient’s clinical progression status. From left to right, aneuploidy score from ref[9], proportion of genome undergoing LOH, fraction of samples bearing whole genome duplication (WGD) and *TP53* alterations. Black dots represent the median values. "*", two-sided Fisher’s exact test adjusted p-value < 0.01 for discrete features (WGD and *TP53*) and two-sided Mann-Whitney adjusted p-value < 0.01 for the continuous features.

Metastatic PRAD tumors had much higher genomic instability rates (i.e., genome aneuploidy, LOH rates, WGD duplication and *TP53* mutations) compared to both primary groups (Suppl. Fig. 20). In PANET, metastatic tumors also showed a general trend towards higher genomic instability indicators (i.e., *TP53* mutations, higher aneuploidy score, higher LOH rates and higher WGD rates). However, we observed that primary PANET patients that progressed/relapsed (PROG/RL group) displayed higher rates of LOH compared to those with better clinical progression. Conversely, WGD rates were higher in good prognosis PANET patients (ST/RM). This is consistent with the fact that WGD and RPCL are independent events and that WGD is a generally

late event in RCPL+ patients [10]. Further analysis, based on paired biopsies are necessary to further understand the interplay of RCPL and WGD in PANET evolution.

Tumor mutation burden and mutational signatures

We then compared the tumor mutation burden (i.e., SNVs, IDs and DBSs mutation burden) between metastatic and primary PANET and PRAD tumors according to the clinical progression annotation (Suppl. Fig. 21). Our data revealed that both in PANET and PRAD there was a significant increase of the three types of mutations in metastatic tumors compared to both groups of primaries. However, in both cancer types, the differences were substantially higher when compared to the ST/RM group (more relatively indolent primaries) than with the ST/RM group (tumors that progressed) (see below).

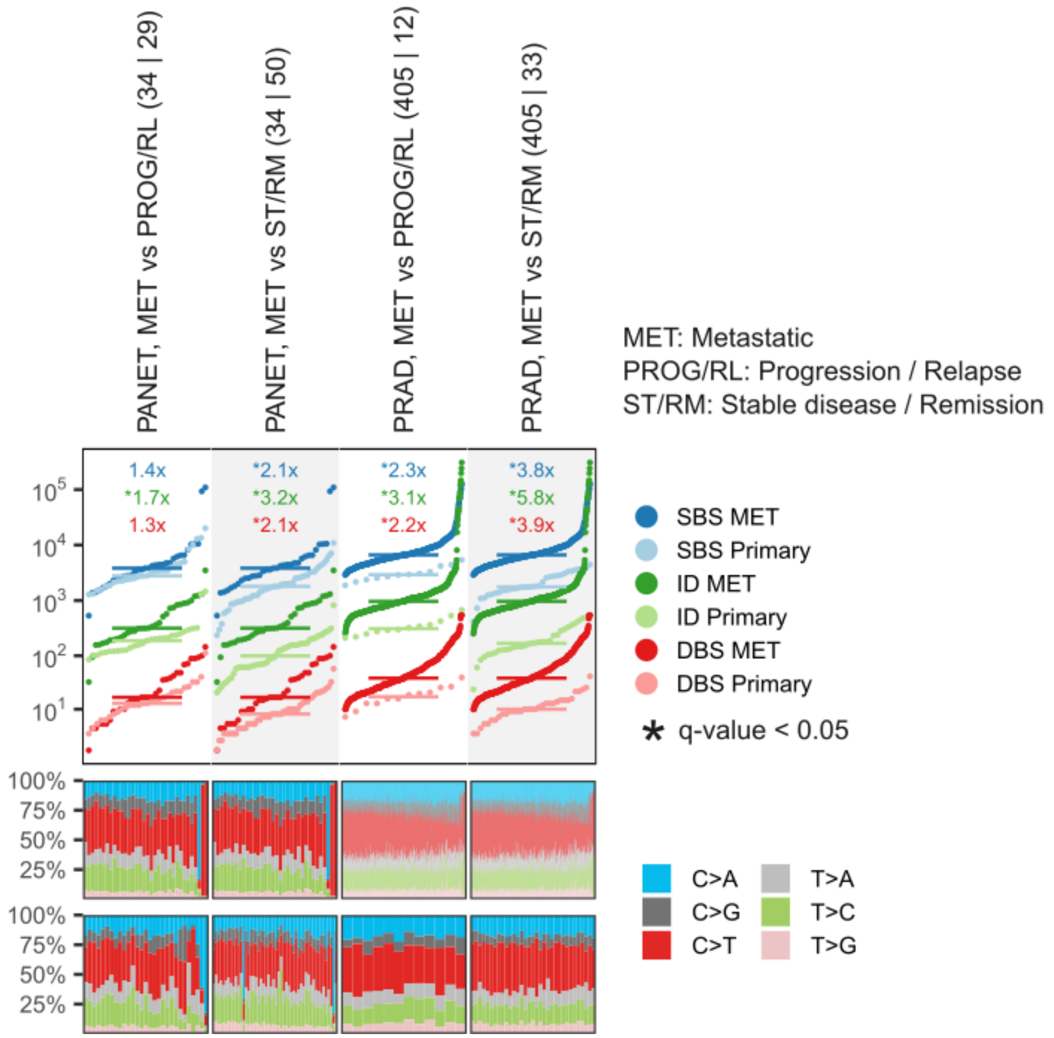

Supplementary Figure 21: Top panel, cumulative distribution function plot (samples were ranked independently for each variant type) of tumor mutation burden for each cancer type for SBS (blue), IDs (green) and DBS (red). Primary tumors are split according to the patient’s clinical progression status. Horizontal lines represent median values. Bottom panel, SBS mutational spectra of metastatic (top) and primary (bottom) tumor patients. Patients are ordered according to their TMB burden. “*”, two-sided Mann-Whitney adjusted p-value < 0.05.

We next performed a comparison of the mutational signature absolute exposures (Suppl. Fig. 22). For this analysis, it is important to mention that the differences in significance relative to exposure in metastatic tumors are strongly influenced by the limited sample size of the primary subgroups (e.g., only 12 patients in the PROG/RL group of prostate). Instead, the median exposure by primary subgroup should be then better used to recognize potential differences with metastatic tumors (i.e., right side color of the dots, see Suppl. Fig. 22 below).

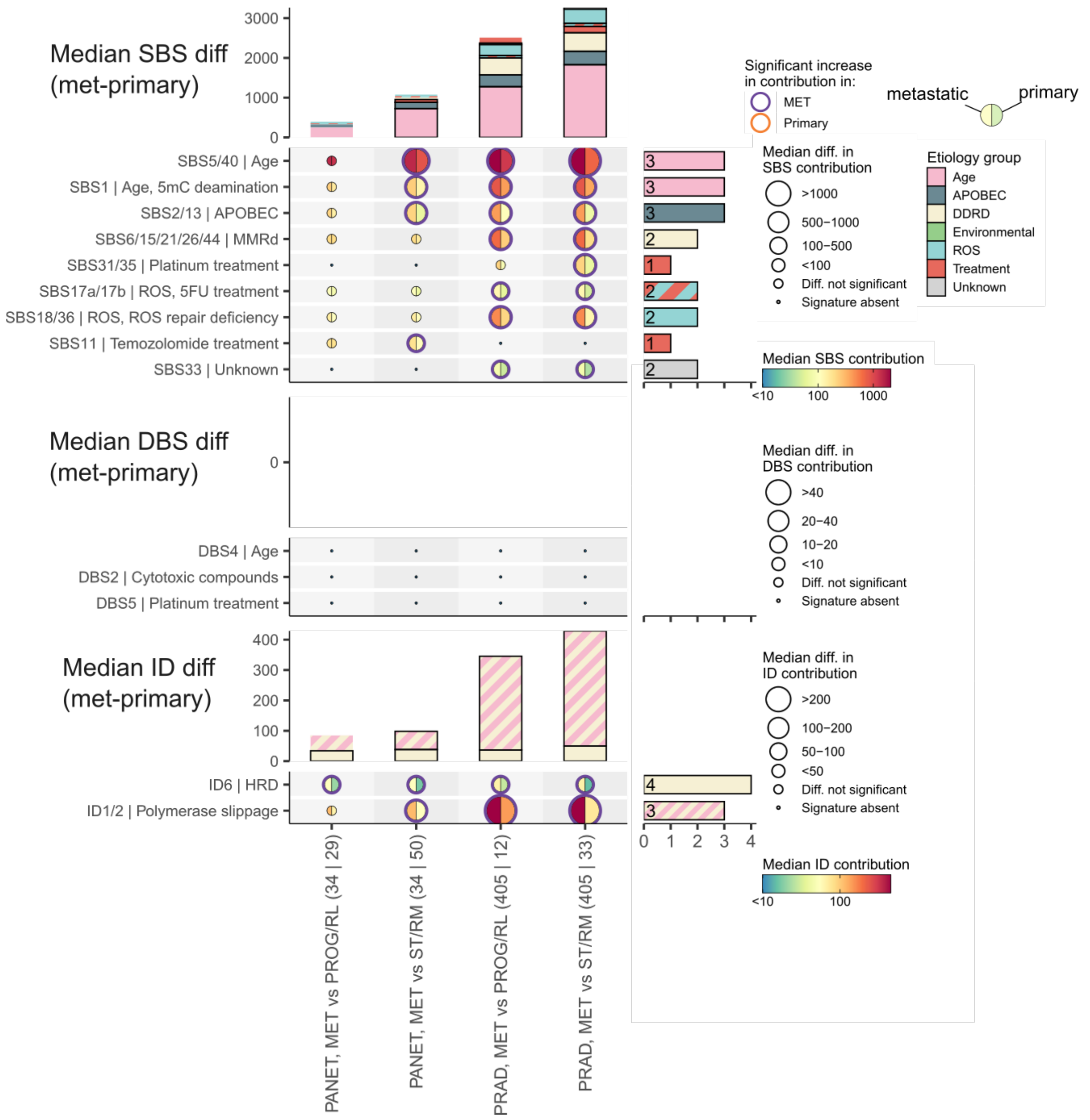

Supplementary Figure 22: Top panel, moon plot representing the mutational burden differences attributed to each mutational signature in metastatic (left) and primary (right) tumors from cancer types. Primary tumors are split according to the patient’s clinical progression status. Horizontal lines represent median values. Edge thickness and colors represent significant differences (two-sided Mann-Whitney adjusted p-value<0.05, ±1.4x fold change) and the direction of the enrichment, respectively. The size of circles are proportionate to the mutation burden difference. Right bars, number of metastatic cancer types with a mutational signature significant enrichment. Top stacked bars represent the cumulative signature exposure difference. Thicker bar edge lines represent significance. Bars are coloured according to the annotated etiology. Only mutational signatures with known etiology or with at least one cancer type with significant metastatic enrichment are included. Mid and bottom panels equivalent for DBS and IDs, respectively. Diff., difference. Muts. mutations. Sig., mutational signature. Mut. mutational. Susp., suspected. Def., deficiency.

Our results revealed that metastatic tumors showed higher mutational load contribution across most of the operative mutational processes in these tumors, which in turn relates to the substantial increased TMB in metastasis compared to both primary groups (see above). However, we observed a tendency towards increased mutational signature exposure in worse prognosis tumors (PROG/RL) compared to better prognosis tumors (ST/RM). Specifically, the data displayed a moderate enrichment in APOBEC mutational load in primary PROG/RL tumors compared to primary ST/RM tumors in both PRAD and PANET (PANET APOBEC median sample exposure PROG/RL=139, ST/RM=40, MET=215; PRAD APOBEC median exposure PROG/RL=111, ST/RM=51, MET=395 in PRAD). Further analysis (ideally relying on paired longitudinal biopsies) are required to unravel whether enhanced APOBEC activity is per-se a driver of late-stage evolution and/or is the natural consequence of richer soil to perform its mutagenic activity (e.g., higher availability of single stranded DNA). Similarly, both in PRAD and PANET we observed a tendency towards higher SBS1 mutation burden in patients that progressed/relapsed compared to the ST/RM group, which in both cases was significantly lower than the average contribution in metastatic tumors (PANET SBS1 median sample exposure PROG/RL=158, ST/RM=108, MET=200; PRAD SBS1 median exposure PROG/RL=353, ST/RM=298, MET=782 in PRAD). Of note, this trend was still observed after correcting by patient’s age at biopsy (Suppl. Fig. 23), suggesting that yearly SBS1 mutation rate increases as PRAD/PANET tumor progress. The mutational exposure for the rest of operative mutational processes generally showed consistent trends (see Supp. Table 3).

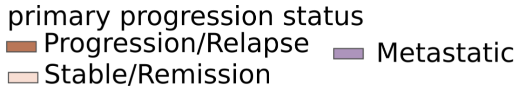

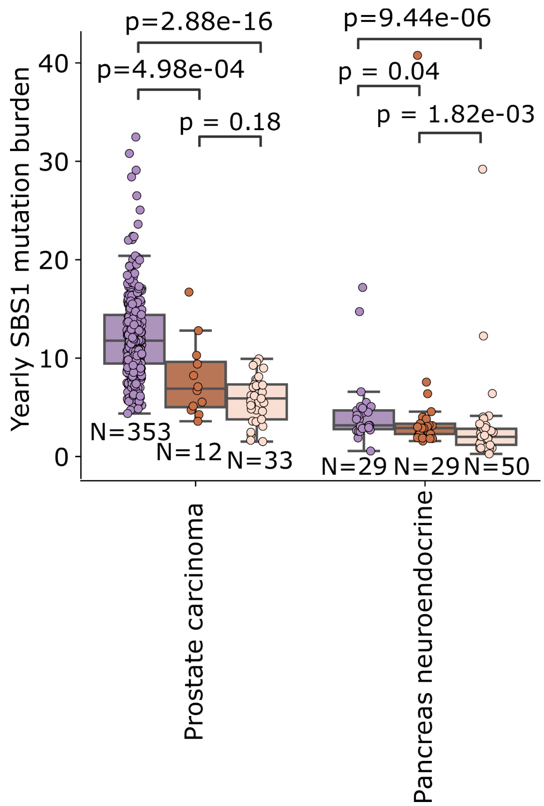

Supplementary Figure 23: Comparison of the age-adjusted SBS1 mutation burden between metastatic tumors (purple), patients with primary tumors that progressed/relapsed (brown) and patients with primary tumors that were stable or in remission (pink) in prostate (left) and pancreas neuroendocrine (right) carcinomas. p, two-sided Mann-Whitney p-value. Box-plots: center line, median; box limits, first and third quartiles; whiskers, lowest/highest data points at first quartile minus/plus 1.5× IQR. N, number of tumor samples.

Structural variant burden

We then compared the structural variant burden for each primary subgroup compared to the metastatic tumors from Hartwig (Suppl. Fig. 24). Our data showed that both in PANET and PRAD there was a significant increase of structural variants, including deletions, duplications and complex events; in metastatic tumors compared to both groups of primaries. However, consistently with the mutation mutation burden patterns, in both cancer types the differences were substantially higher when compared to the ST/RM group (more relatively indolent primaries) than with the ST/RM group (tumors that progressed) (i.e., x2.5 increase respective to PROG/RL group and x5.6 increase compared to ST/RM group in PANET, x3.0 increase respective to PROG/RL and x7.1 compared to ST/RM group in PRAD, see below).

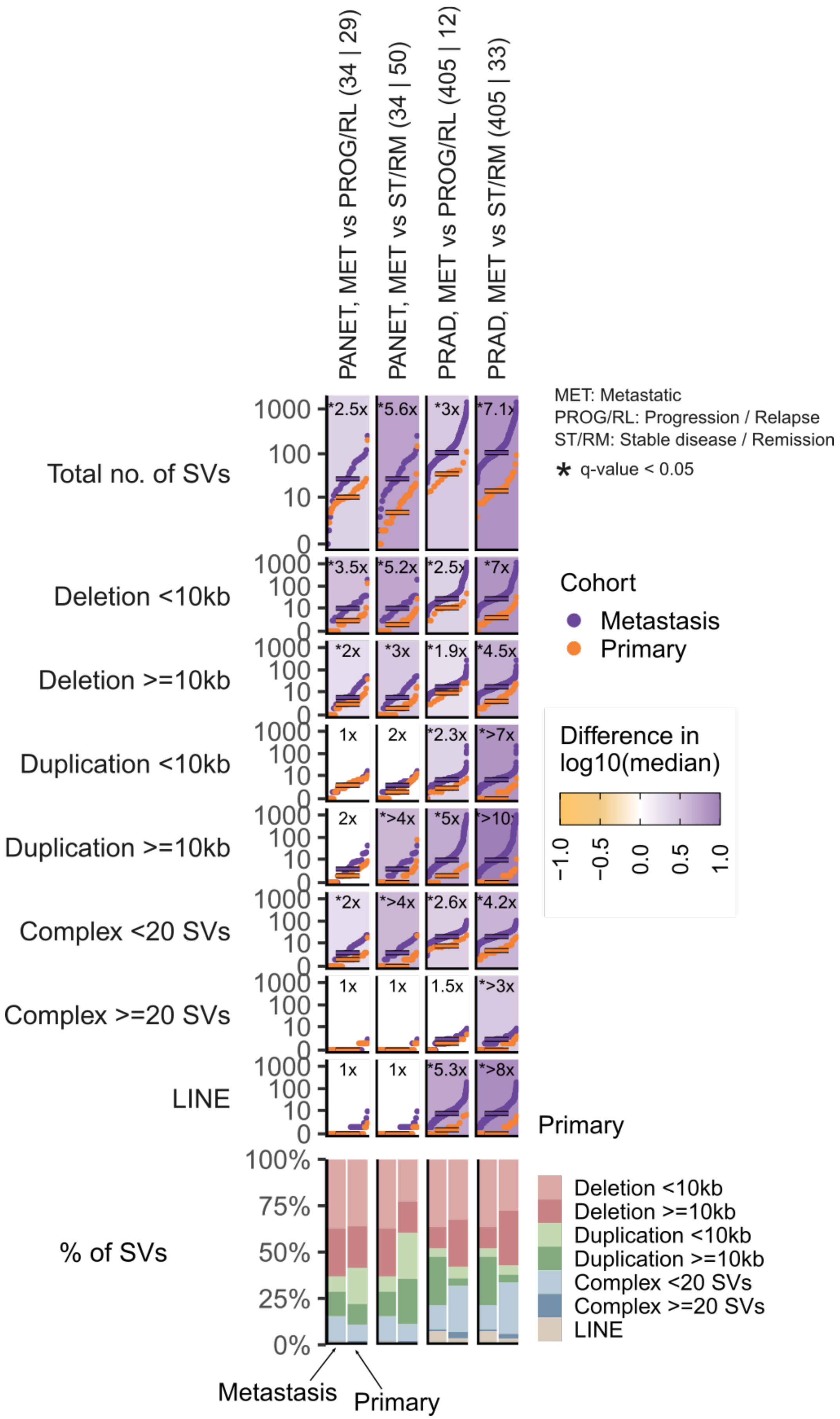
PANET, MET vs PROG/RL (34 | 29)
PANET, MET vs ST/RM (34 | 50)
PRAD, MET vs PROG/RL (405 | 12)
PRAD, MET vs ST/RM (405 | 33)
MET: Metastatic
PROG/RL: Progression / Relapse
ST/RM: Stable disease / Remission
* q-value < 0.05
Total no. of SVs
*2.5x
*5.6x
*3x
*7.1x
Deletion <10kb
*3.5x
*5.2x
*2.5x
*7x
Deletion >=10kb
*2x
*3x
*1.9x
*4.5x
Duplication <10kb
1x
2x
*2.3x
*>7x
Duplication >=10kb
2x
*>4x
*5x
*>10
Complex <20 SVs
*2x
*>4x
*2.6x
*4.2x
Complex >=20 SVs
1x
1x
1.5x
*>3x
LINE
1x
1x
*5.3x
*>8x
1000
100
10
0
Cohort
Metastasis
Primary
Difference in
log10(median)
−1.0
−0.5
0.0
0.5
1.0
% of SVs
100%
75%
50%
25%
0%
Primary
Deletion <10kb
Deletion >=10kb
Duplication <10kb
Duplication >=10kb
Complex <20 SVs
Complex >=20 SVs
LINE
Metastasis
Primary

Supplementary Figure 24: S-plots, cumulative distribution function plot (samples ranked independently for each SV type) of tumor mutation burden for each cancer type for (from top to the bottom) the aggregated structural variant (SV) burden, small deletions (<10kb), large deletions (>=10kb), small duplications (<10kb), large duplications (>=10kb), complex events (<20 breakpoints), complex events (>=20 breakpoints) and LINEs insertions. Primary tumors are grouped according to the patient's clinical progression status. Horizontal lines represent median values. Backgrounds are coloured according to the relative enrichment, defined as: $\log_{10}$(median SV type burden in metastatic tumors + 1) − $\log_{10}$(median SV type burden in primary tumors + 1). Coloured backgrounds and “+” are displayed when a two-sided Mann-Whitney comparison renders a significant q-value < 0.05. Fold change labels are displayed with ‘>’ when the SV burden for primary tumors is 0 (see methods for more details). For each cancer type, bottom bar plots represent the relative fraction of each SV type in the metastatic (left) and primary (right) datasets.

Drivers

As shown in the main manuscript, comparison of the number of driver events per sample revealed that both prostate and pancreatic neuroendocrine metastatic tumors showed an increased number of mean driver events per sample compared to primary (see Suppl. Fig. 25 below and Fig. 4a).

This increase was also retained when grouping primary tumors according to the annotated clinical progression (ST/RM or PROG/RL). Nevertheless, we observed that the magnitude of the differences was considerably stronger (i.e., almost 2-fold) when comparing metastatic tumors to primary tumors with a favorable prognosis (ST/RM, increase of +2.68 in PANET and +3.93 in PRAD) compared to primary tumors that relapse/progress (PROG/RL, increase of +1.25 in PANET and +2.04 in PRAD).

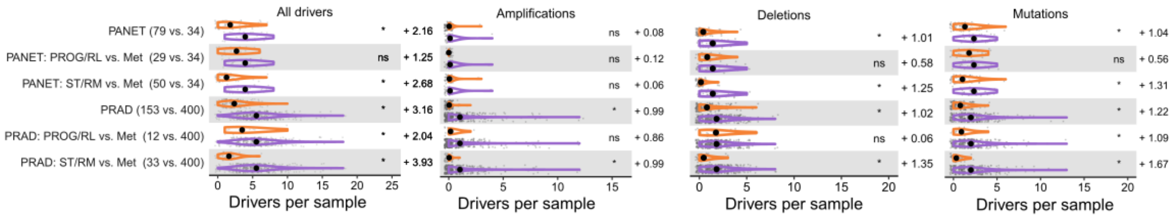

Supplementary Figure 25: Cancer type specific distribution of number of driver alterations, amplifications, deletions and mutations per patient in primary (top) and metastatic (bottom). Black dots represent the mean values. Labels display mean differences (metastatic - primary) in cancer types with a significant difference. Primary tumors are grouped according to the patient's clinical progression status. Ns, non-significant p-value. “*”, two-sided Mann-Whitney adjusted p-value < 0.01.

We next compared driver-specific frequency differences between primary progression groups compared to metastatic tumors (see Suppl. Fig. 26 below). As expected, bad prognosis associated drivers, such as *TP53* and *CDKN2A*, showed higher frequency in primary PROG/RL tumors compared to good prognosis groups (ST/RM). However, metastatic tumors still displayed an increased alteration frequency of these genes compared to primary aggressive tumors. For instance, 3/29 (10%) PANET PROG/RL patients harbored *CDK2NA* deletions, whereas 8 out 34 (24%) of the metastatic PANET patients harbored such deletions. Similarly, *TP53* mutations

showed a 16% (2/12) mutation frequency in primary PRAD tumors with bad prognosis whereas metastatic PRAD tumors showed a 40% (161/400) mutation frequency, which was in line with the findings reported in a large cohort of primary-metastatic paired prostate cancer tumors [11]. Interestingly, of all the screened driver alterations, only *PTEN* deletions showed comparable frequency between primary aggressive and metastatic PRAD tumors (5/12 (41%) in PROG/RL and 168/400 (42%) in met). Finally, driver alterations such as *AR* mutation/amplifications in PRAD and *CREBBP*/*TSC2* mutations in PANET were basically absent in the two primary cohorts, indicating that these alterations are associated with metastatic transformation and/or resistance to cancer therapies.

Primary (mutated / total samples)

Metastatic (mutated / total samples)

Cancer type	Gene	Cramer's V	P-value	P-value (FDR adjusted)
**Amplification**				
PRAD (1/153 vs. 133/400)	AR	0.34	< 0.01	< 0.01
PRAD_PROG/RL (0/12 vs. 133/400)	AR	0.12	< 0.05	n.s.
PRAD_ST/RM (0/33 vs. 133/400)	AR	0.19	< 0.01	< 0.01
**Deletion**				
PANET (3/79 vs. 8/34)	CDKN2A	0.31	< 0.01	< 0.01
PANET_PROG/RL (3/29 vs. 8/34)	CDKN2A	0.17	n.s.	n.s.
PANET_ST/RM (0/50 vs. 8/34)	CDKN2A	0.39	< 0.01	< 0.01
PRAD (30/153 vs. 168/400)	PTEN	0.21	< 0.01	< 0.01
PRAD_PROG/RL (5/12 vs. 168/400)	PTEN	0.00	n.s.	n.s.
PRAD_ST/RM (2/33 vs. 168/400)	PTEN	0.20	< 0.01	< 0.01
PRAD (3/153 vs. 61/400)	TP53	0.19	< 0.01	< 0.01
PRAD_PROG/RL (1/12 vs. 61/400)	TP53	0.03	n.s.	n.s.
PRAD_ST/RM (1/33 vs. 61/400)	TP53	0.09	n.s.	n.s.
**Mutation**				
PRAD (1/153 vs. 73/400)	AR	0.23	< 0.01	< 0.01
PRAD_PROG/RL (0/12 vs. 73/400)	AR	0.08	n.s.	n.s.
PRAD_ST/RM (0/33 vs. 73/400)	AR	0.13	< 0.01	n.s.
PANET (0/79 vs. 5/34)	CREBBP	0.33	< 0.01	< 0.01
PANET_PROG/RL (0/29 vs. 5/34)	CREBBP	0.27	n.s.	n.s.
PANET_ST/RM (0/50 vs. 5/34)	CREBBP	0.31	< 0.01	< 0.05
PRAD (11/153 vs. 161/400)	TP53	0.32	< 0.01	< 0.01
PRAD_PROG/RL (2/12 vs. 161/400)	TP53	0.08	n.s.	n.s.
PRAD_ST/RM (0/33 vs. 161/400)	TP53	0.22	< 0.01	< 0.01
PANET (2/79 vs. 11/34)	TSC2	0.43	< 0.01	< 0.01
PANET_PROG/RL (1/29 vs. 11/34)	TSC2	0.37	< 0.01	< 0.05
PANET_ST/RM (1/50 vs. 11/34)	TSC2	0.43	< 0.01	< 0.01

Supplementary Figure 26: Cancer type specific comparison of driver alteration frequency between primary tumors grouped by patient’s clinical progression status and metastatic tumors.

In conclusion, our data revealed that the driver landscape of metastatic PANET and PRAD tumors were quantitatively (i.e., higher number of drivers) and qualitatively (i.e., discrepancies in certain driver alteration frequency) different from both good and worse prognosis primary tumors. However, bad prognosis primary tumors shower less accentuated differences, which may reflect closer evolutionary distance to late-stage and treatment-resistant metastatic tumors.

Conclusion

Our analysis revealed that metastatic prostate and pancreatic neuroendocrine tumors are genomically different from their primary counterparts. However, the magnitude of the differences was higher when comparing to primary patients with a relatively good prognosis than when we compared to primary tumors with worse prognosis. Moreover, our results also highlighted some potential differences associated with worse prognosis and metastatic transformation (e.g., APOBEC activity, RCPL in pancreatic neuroendocrine, an increased SV burden in worse prognosis patients, etc). However, in our opinion, dedicated studies relying on primary-metastatic paired biopsies are better suited to unequivocally delineate the role and the relative timing of such observations in tumor evolution.

Primary and metastatic comparison of genomic features by metastatic biopsy location

Dataset annotation

We aimed to elucidate whether metastatic location may have a strong influence in the reported differences between primary and metastatic tumors. To perform this analysis we used the metastatic location annotation from Supp. Table 1, comparing all primary tumors to each metastatic group with a sufficient number of samples (at least 5 samples were required). It is important to remark that the Hartwig Medical Foundation metastatic dataset includes metastatic biopsies from patients enrolled across multiple dutch hospitals. Consequently, despite the important efforts to harmonize the clinical data, this is not a centralized study with unified criteria to define the metastatic biopsy location. For that reason we have simplified the analysis using three groups of metastatic locations: local metastasis, lymph node (LN) metastasis (including both locoregional and distant lymph nodes, because we lacked of complete annotation of exact LN location) and distant mets, encompassing metastatic lesions outside of the primary tumor tissue.

Tumor clonality

We systematically evaluated whether metastatic location influences tumor clonality. For each cancer type, we then compared the average primary tumor clonality to the metastatic tumors grouped by metastatic biopsy location. Overall, as reported in the main manuscript (see Fig. 1c and Extended Data Fig. 1b), distant and LN mets tended to have higher tumor clonality compared to local metastatic biopsies (see Suppl. Fig. 27 below). This was particularly noticeable for breast, colorectal and esophageal carcinomas, whereas for other cancer types, such as prostate or skin melanoma, we did not observe large differences in tumor clonality across metastatic locations.

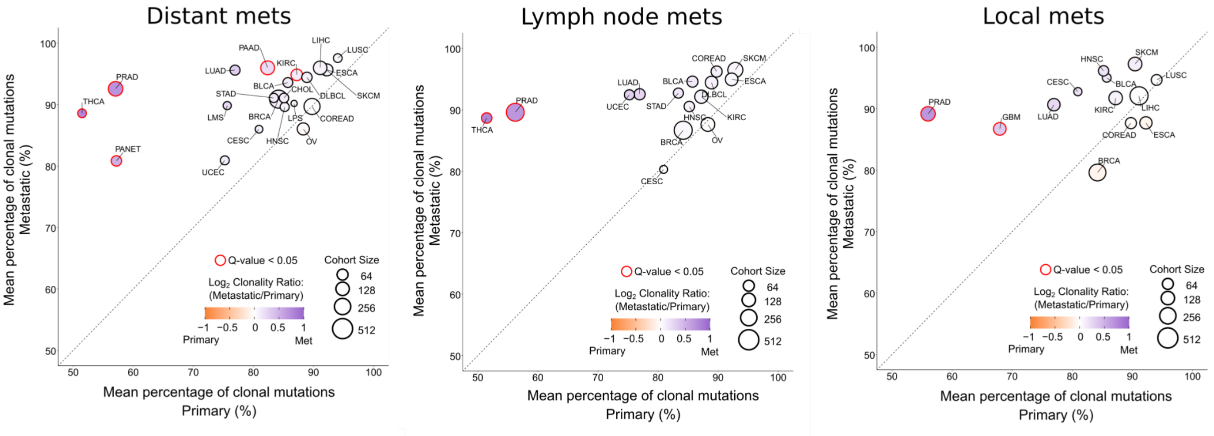

Supplementary Figure 27: Mean percentage of clonal mutations in primary (x-axis) and metastatic (y-axis) tumors across cancer types. Metastatic tumors are grouped according to biopsy location, from left to the right, distant metastasis, lymph node metastasis and local metastasis. Dots are coloured according to Log_2 of the clonality ratio (metastatic divided by primary). Size of dots are proportional to the total number of samples (primary and metastatic). Red edge lines represent a two-sided Mann-Whitney adjusted p-value < 0.05.

It is also important to mention that in the Hartwig dataset, LN biopsies, which have been reported to undergo wider evolutionary bottleneck than distant metastases[12] (i.e., higher intra-tumor heterogeneity), only displayed a modest and non-homogeneous decrease in tumor clonality compared to distant metastasis (see breast and esophageal carcinomas Fig. 1c and kidney renal clear cell, lung adeno, prostate carcinoma in Extended Data Fig. 1b). The lack of significant differences between LN and distant biopsies could be explained by an enrichment of distant LN biopsies (i.e., non locoregional), but this is currently impossible to be formally assessed given the lack of harmonized clinical annotation for this information in the Hartwig dataset.

Karyotype and genomic instability indicators

We analyzed whether metastatic location may have an influence in the reported karyotype.

The tumor karyotype was highly cancer type specific and generally conserved across different metastatic locations (Suppl. Fig. 28). Few statistical significance differences were observed,

although they were likely linked to different sample sizes representation across metastatic location groups (e.g., prostate carcinoma).

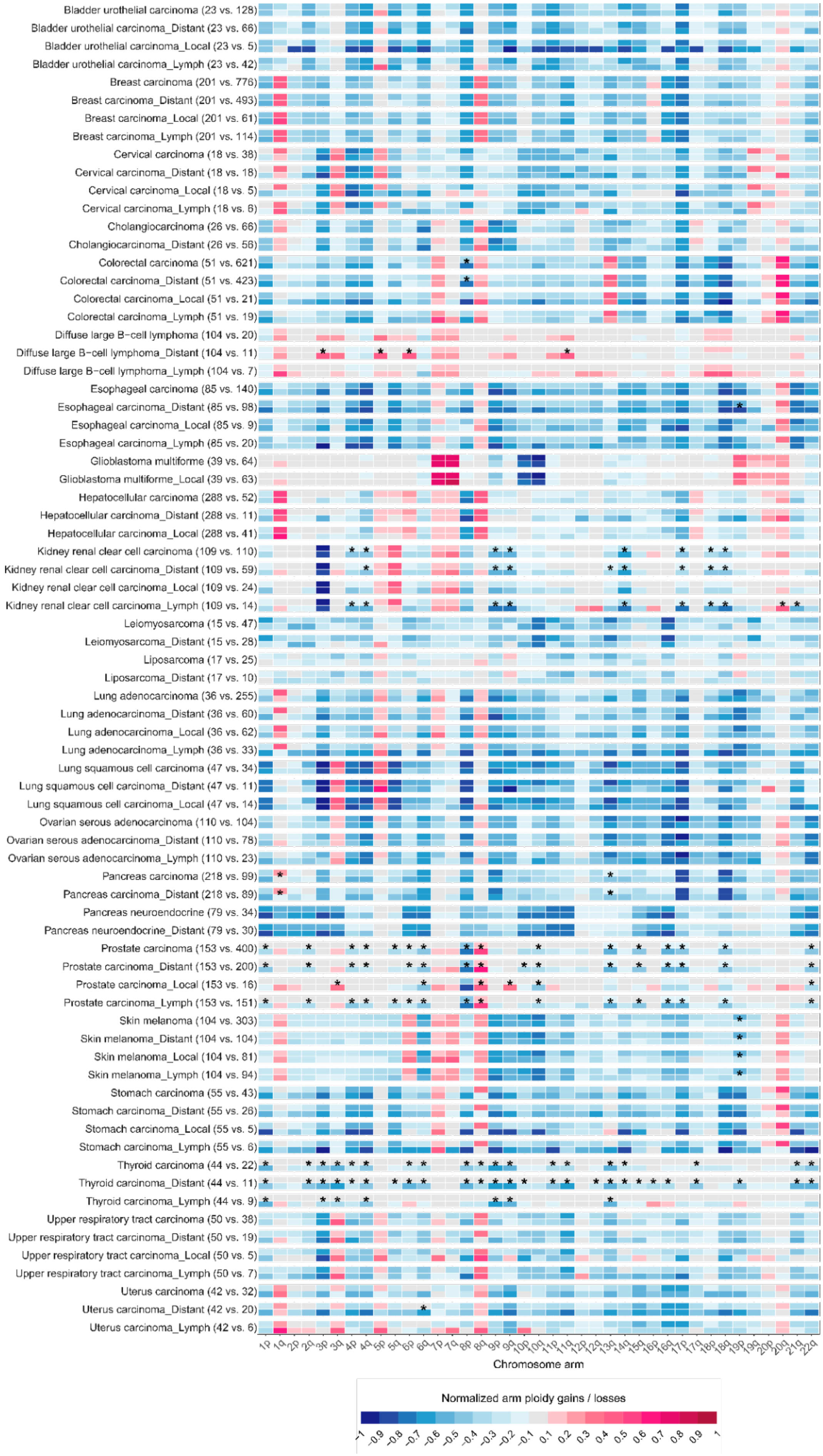
Bladder urothelial carcinoma (23 vs. 128)
Bladder urothelial carcinoma_Distant (23 vs. 66)
Bladder urothelial carcinoma_Local (23 vs. 5)
Bladder urothelial carcinoma_Lymph (23 vs. 42)
Breast carcinoma (201 vs. 776)
Breast carcinoma_Distant (201 vs. 493)
Breast carcinoma_Local (201 vs. 61)
Breast carcinoma_Lymph (201 vs. 114)
Cervical carcinoma (18 vs. 38)
Cervical carcinoma_Distant (18 vs. 18)
Cervical carcinoma_Local (18 vs. 5)
Cervical carcinoma_Lymph (18 vs. 6)
Cholangiocarcinoma (26 vs. 66)
Cholangiocarcinoma_Distant (26 vs. 58)
Colorectal carcinoma (51 vs. 621)
Colorectal carcinoma_Distant (51 vs. 423)
Colorectal carcinoma_Local (51 vs. 21)
Colorectal carcinoma_Lymph (51 vs. 19)
Diffuse large B−cell lymphoma (104 vs. 20)
Diffuse large B−cell lymphoma_Distant (104 vs. 11)
Diffuse large B−cell lymphoma_Lymph (104 vs. 7)
Esophageal carcinoma (85 vs. 140)
Esophageal carcinoma_Distant (85 vs. 98)
Esophageal carcinoma_Local (85 vs. 9)
Esophageal carcinoma_Lymph (85 vs. 20)
Glioblastoma multiforme (39 vs. 64)
Glioblastoma multiforme_Local (39 vs. 63)
Hepatocellular carcinoma (288 vs. 52)
Hepatocellular carcinoma_Distant (288 vs. 11)
Hepatocellular carcinoma_Local (288 vs. 41)
Kidney renal clear cell carcinoma (109 vs. 110)
Kidney renal clear cell carcinoma_Distant (109 vs. 59)
Kidney renal clear cell carcinoma_Local (109 vs. 24)
Kidney renal clear cell carcinoma_Lymph (109 vs. 14)
Leiomyosarcoma (15 vs. 47)
Leiomyosarcoma_Distant (15 vs. 28)
Liposarcoma (17 vs. 25)
Liposarcoma_Distant (17 vs. 10)
Lung adenocarcinoma (36 vs. 255)
Lung adenocarcinoma_Distant (36 vs. 60)
Lung adenocarcinoma_Local (36 vs. 62)
Lung adenocarcinoma_Lymph (36 vs. 33)
Lung squamous cell carcinoma (47 vs. 34)
Lung squamous cell carcinoma_Distant (47 vs. 11)
Lung squamous cell carcinoma_Local (47 vs. 14)
Ovarian serous adenocarcinoma (110 vs. 104)
Ovarian serous adenocarcinoma_Distant (110 vs. 78)
Ovarian serous adenocarcinoma_Lymph (110 vs. 23)
Pancreas carcinoma (218 vs. 99)
Pancreas carcinoma_Distant (218 vs. 89)
Pancreas neuroendocrine (79 vs. 34)
Pancreas neuroendocrine_Distant (79 vs. 30)
Prostate carcinoma (153 vs. 400)
Prostate carcinoma_Distant (153 vs. 200)
Prostate carcinoma_Local (153 vs. 16)
Prostate carcinoma_Lymph (153 vs. 151)
Skin melanoma (104 vs. 303)
Skin melanoma_Distant (104 vs. 104)
Skin melanoma_Local (104 vs. 81)
Skin melanoma_Lymph (104 vs. 94)
Stomach carcinoma (55 vs. 43)
Stomach carcinoma_Distant (55 vs. 26)
Stomach carcinoma_Local (55 vs. 5)
Stomach carcinoma_Lymph (55 vs. 6)
Thyroid carcinoma (44 vs. 22)
Thyroid carcinoma_Distant (44 vs. 11)
Thyroid carcinoma_Lymph (44 vs. 9)
Upper respiratory tract carcinoma (50 vs. 38)
Upper respiratory tract carcinoma_Distant (50 vs. 19)
Upper respiratory tract carcinoma_Local (50 vs. 5)
Upper respiratory tract carcinoma_Lymph (50 vs. 7)
Uterus carcinoma (42 vs. 32)
Uterus carcinoma_Distant (42 vs. 20)
Uterus carcinoma_Lymph (42 vs. 6)
1p 1q 2p 2q 3p 3q 4p 4q 5p 5q 6p 6q 7p 7q 8p 8q 9p 9q 10p 10q 11p 11q 12p 12q 13q 14q 15q 16p 16q 17p 17q 18p 18q 19p 19q 20p 20q 21q 22q
Chromosome arm
Normalized arm ploidy gains / losses
-1 -0.9 -0.8 -0.7 -0.6 -0.5 -0.4 -0.3 -0.2 -0.1 0.1 0.2 0.3 0.4 0.5 0.6 0.7 0.8 0.9 1

Supplementary Figure 28: Tumor karyotype. Heatmap representing the normalized mean chromosome arm ploidy gains and losses relative to the expected 2n ploidy status in primary (top) and metastatic (bottom) tumors from cancer types. For each cancer type, metastatic tumors are grouped according to biopsy location, from top to the bottom, all grouped biopsy locations, distant metastasis, local metastasis and lymph node metastasis. "*", significantly different mean distributions between primary and metastatic tumors (two-sided Mann-Whitney adjusted p-value < 0.01).

We next investigated differences in genomic instability indicators (Suppl. Fig. 29). Globally, we did not observe a general trend towards enrichment/depletion indicators in any metastatic location. Consequently, genomic instability differences with the primary tumors were also consistent across different metastatic location groups. The most prominent exception was the LN lung adenocarcinoma metastasis, which showed higher rates of aneuploidy scores, LOH rates, WGD and *TP53* alterations compared to other metastatic locations and to the primary lung adenocarcinoma tumors.

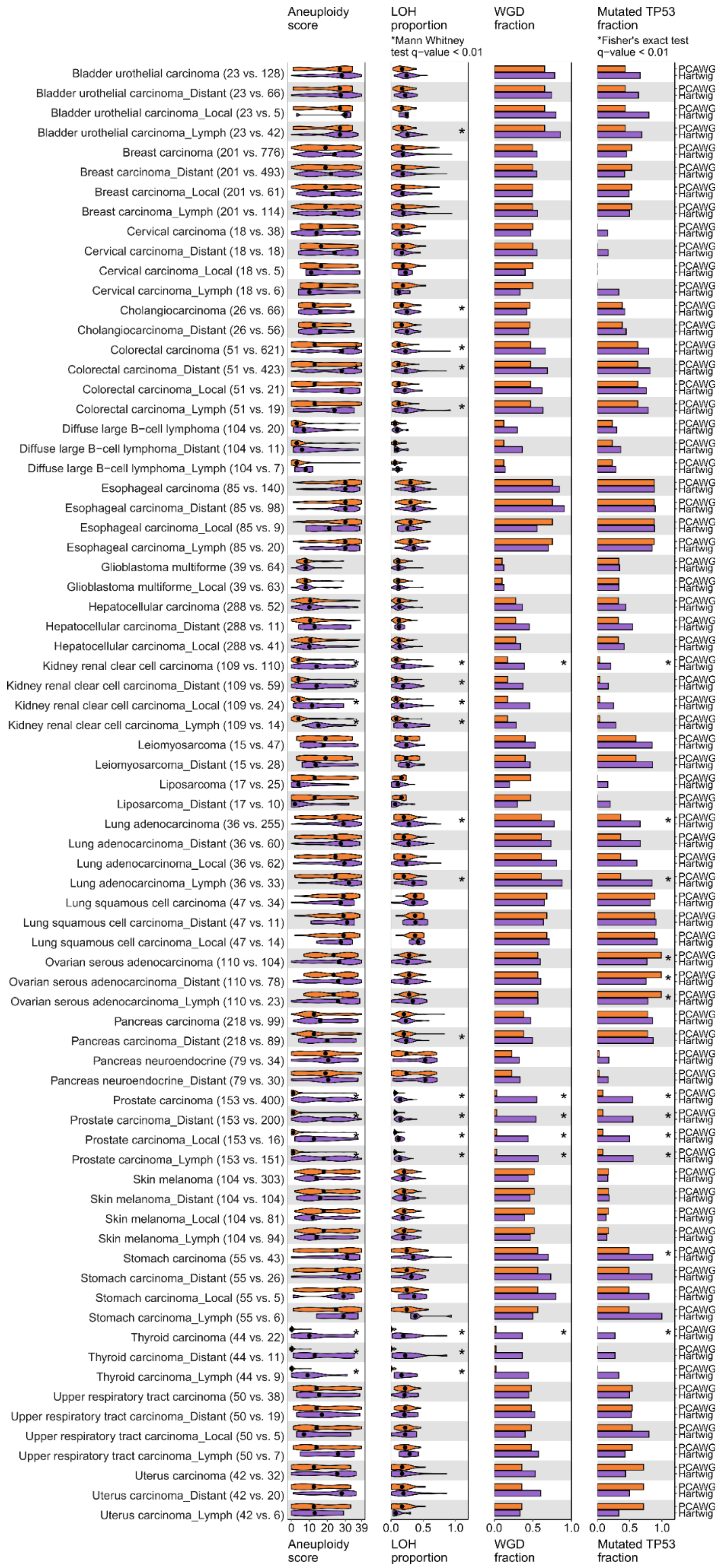
Aneuploidy score
LOH proportion
*Mann Whitney test q-value < 0.01
WGD fraction
Mutated TP53 fraction
*Fisher's exact test q-value < 0.01
Bladder urothelial carcinoma (23 vs. 128)
Bladder urothelial carcinoma_Distant (23 vs. 66)
Bladder urothelial carcinoma_Local (23 vs. 5)
Bladder urothelial carcinoma_Lymph (23 vs. 42)
Breast carcinoma (201 vs. 776)
Breast carcinoma_Distant (201 vs. 493)
Breast carcinoma_Local (201 vs. 61)
Breast carcinoma_Lymph (201 vs. 114)
Cervical carcinoma (18 vs. 38)
Cervical carcinoma_Distant (18 vs. 18)
Cervical carcinoma_Local (18 vs. 5)
Cervical carcinoma_Lymph (18 vs. 6)
Cholangiocarcinoma (26 vs. 66)
Cholangiocarcinoma_Distant (26 vs. 56)
Colorectal carcinoma (51 vs. 621)
Colorectal carcinoma_Distant (51 vs. 423)
Colorectal carcinoma_Local (51 vs. 21)
Colorectal carcinoma_Lymph (51 vs. 19)
Diffuse large B−cell lymphoma (104 vs. 20)
Diffuse large B−cell lymphoma_Distant (104 vs. 11)
Diffuse large B−cell lymphoma_Lymph (104 vs. 7)
Esophageal carcinoma (85 vs. 140)
Esophageal carcinoma_Distant (85 vs. 98)
Esophageal carcinoma_Local (85 vs. 9)
Esophageal carcinoma_Lymph (85 vs. 20)
Glioblastoma multiforme (39 vs. 64)
Glioblastoma multiforme_Local (39 vs. 63)
Hepatocellular carcinoma (288 vs. 52)
Hepatocellular carcinoma_Distant (288 vs. 11)
Hepatocellular carcinoma_Local (288 vs. 41)
Kidney renal clear cell carcinoma (109 vs. 110)
Kidney renal clear cell carcinoma_Distant (109 vs. 59)
Kidney renal clear cell carcinoma_Local (109 vs. 24)
Kidney renal clear cell carcinoma_Lymph (109 vs. 14)
Leiomyosarcoma (15 vs. 47)
Leiomyosarcoma_Distant (15 vs. 28)
Liposarcoma (17 vs. 25)
Liposarcoma_Distant (17 vs. 10)
Lung adenocarcinoma (36 vs. 255)
Lung adenocarcinoma_Distant (36 vs. 60)
Lung adenocarcinoma_Local (36 vs. 62)
Lung adenocarcinoma_Lymph (36 vs. 33)
Lung squamous cell carcinoma (47 vs. 34)
Lung squamous cell carcinoma_Distant (47 vs. 11)
Lung squamous cell carcinoma_Local (47 vs. 14)
Ovarian serous adenocarcinoma (110 vs. 104)
Ovarian serous adenocarcinoma_Distant (110 vs. 78)
Ovarian serous adenocarcinoma_Lymph (110 vs. 23)
Pancreas carcinoma (218 vs. 99)
Pancreas carcinoma_Distant (218 vs. 89)
Pancreas neuroendocrine (79 vs. 34)
Pancreas neuroendocrine_Distant (79 vs. 30)
Prostate carcinoma (153 vs. 400)
Prostate carcinoma_Distant (153 vs. 200)
Prostate carcinoma_Local (153 vs. 16)
Prostate carcinoma_Lymph (153 vs. 151)
Skin melanoma (104 vs. 303)
Skin melanoma_Distant (104 vs. 104)
Skin melanoma_Local (104 vs. 81)
Skin melanoma_Lymph (104 vs. 94)
Stomach carcinoma (55 vs. 43)
Stomach carcinoma_Distant (55 vs. 26)
Stomach carcinoma_Local (55 vs. 5)
Stomach carcinoma_Lymph (55 vs. 6)
Thyroid carcinoma (44 vs. 22)
Thyroid carcinoma_Distant (44 vs. 11)
Thyroid carcinoma_Lymph (44 vs. 9)
Upper respiratory tract carcinoma (50 vs. 38)
Upper respiratory tract carcinoma_Distant (50 vs. 19)
Upper respiratory tract carcinoma_Local (50 vs. 5)
Upper respiratory tract carcinoma_Lymph (50 vs. 7)
Uterus carcinoma (42 vs. 32)
Uterus carcinoma_Distant (42 vs. 20)
Uterus carcinoma_Lymph (42 vs. 6)
PCAWG
Hartwig
0 10 20 30 39
0.0 0.5 1.0
Aneuploidy score
LOH proportion
WGD fraction
Mutated TP53 fraction

Supplementary Figure 29: Comparison of four genomic indicators between primary (top) and metastatic (bottom) tumors across cancer types. For each cancer type, metastatic tumors are grouped according to biopsy location, from top to the bottom, all grouped metastatic locations, distant metastasis, local metastasis and lymph node metastasis. Panels, from left to right, aneuploidy score from ref[9], proportion of genome undergoing LOH, fraction of samples bearing whole genome duplication (WGD) and *TP53* alterations. Black dots represent the median values. "*", two-sided Fisher’s exact test adjusted p-value < 0.01 for discrete features (WGD and *TP53*) and two-sided Mann-Whitney adjusted p-value < 0.01 for the continuous features.

Tumor mutation burden and mutational signatures

We next evaluated whether metastatic location may also influence TMB (i.e., SNV, ID and DBS burden). Overall, we did not observe a systemic pattern of enrichment or depletion of TMB burden in any particular metastatic location compared to the primary TMB (Suppl. Fig. 30). The most remarkable exception was a substantial increase of all types of TMB in LUAD LN mets compared to other metastatic locations (see below and Supp. Table 3). Aside from this, four cancer types (i.e., breast, stomach, cervical and prostate carcinomas) showed a moderate increase in TMB in distant mets compared to local biopsies. The increase in TMB in distant metastatic biopsies in these cancer types may reflect later evolutionary stages (i.e., older molecular time), although these comparisons should be taken with caution given limited sample size of some of the metastatic location groups (e.g., esophageal and stomach local with nine and five samples respectively). Nevertheless, in the remaining cancer types we did not observe a strong TMB difference across metastatic locations.

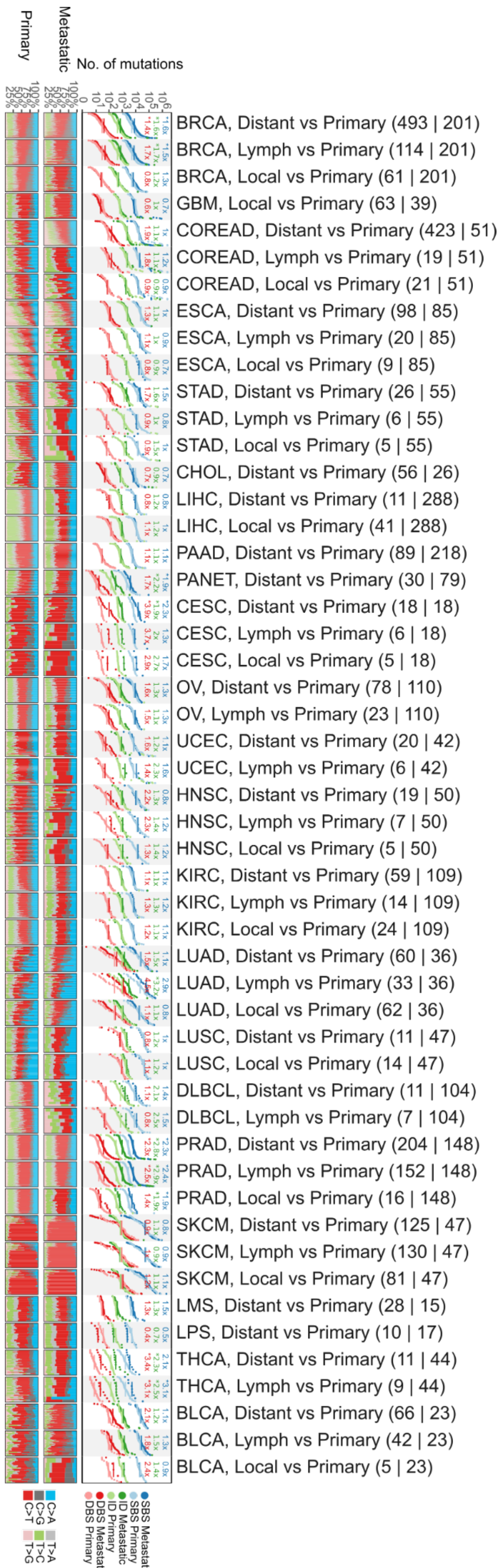
Primary
Metastatic
No. of mutations
BRCA, Distant vs Primary (493 | 201)
BRCA, Lymph vs Primary (114 | 201)
BRCA, Local vs Primary (61 | 201)
GBM, Local vs Primary (63 | 39)
COREAD, Distant vs Primary (423 | 51)
COREAD, Lymph vs Primary (19 | 51)
COREAD, Local vs Primary (21 | 51)
ESCA, Distant vs Primary (98 | 85)
ESCA, Lymph vs Primary (20 | 85)
ESCA, Local vs Primary (9 | 85)
STAD, Distant vs Primary (26 | 55)
STAD, Lymph vs Primary (6 | 55)
STAD, Local vs Primary (5 | 55)
CHOL, Distant vs Primary (56 | 26)
LIHC, Distant vs Primary (11 | 288)
LIHC, Local vs Primary (41 | 288)
PAAD, Distant vs Primary (89 | 218)
PANET, Distant vs Primary (30 | 79)
CESC, Distant vs Primary (18 | 18)
CESC, Lymph vs Primary (6 | 18)
CESC, Local vs Primary (5 | 18)
OV, Distant vs Primary (78 | 110)
OV, Lymph vs Primary (23 | 110)
UCEC, Distant vs Primary (20 | 42)
UCEC, Lymph vs Primary (6 | 42)
HNSC, Distant vs Primary (19 | 50)
HNSC, Lymph vs Primary (7 | 50)
HNSC, Local vs Primary (5 | 50)
KIRC, Distant vs Primary (59 | 109)
KIRC, Lymph vs Primary (14 | 109)
KIRC, Local vs Primary (24 | 109)
LUAD, Distant vs Primary (60 | 36)
LUAD, Lymph vs Primary (33 | 36)
LUAD, Local vs Primary (62 | 36)
LUSC, Distant vs Primary (11 | 47)
LUSC, Local vs Primary (14 | 47)
DLBCL, Distant vs Primary (11 | 104)
DLBCL, Lymph vs Primary (7 | 104)
PRAD, Distant vs Primary (204 | 148)
PRAD, Lymph vs Primary (152 | 148)
PRAD, Local vs Primary (16 | 148)
SKCM, Distant vs Primary (125 | 47)
SKCM, Lymph vs Primary (130 | 47)
SKCM, Local vs Primary (81 | 47)
LMS, Distant vs Primary (28 | 15)
LPS, Distant vs Primary (10 | 17)
THCA, Distant vs Primary (11 | 44)
THCA, Lymph vs Primary (9 | 44)
BLCA, Distant vs Primary (66 | 23)
BLCA, Lymph vs Primary (42 | 23)
BLCA, Local vs Primary (5 | 23)
C>A
C>G
C>T
T>A
T>C
T>G
SBS Metastatic
SBS Primary
ID Metastatic
ID Primary
DBS Metastatic
DBS Primary

Supplementary Figure 30: Top panel, cumulative distribution function plot (samples were ranked independently for each variant type) of tumor mutation burden for each cancer type for SBS (blue), IDs (green) and DBS (red). For each cancer type, metastatic tumors are grouped according to biopsy location, from top to the bottom, distant metastasis, lymph node metastasis and local metastasis. Horizontal lines represent median values. “*”, two-sided Mann-Whitney comparison renders a significant q-value < 0.05. Bottom panel, SBS mutational spectra of metastatic (top) and primary (bottom) tumor patients. Patients are ordered according to their TMB burden.

To provide insights into the potential mutational processes responsible for the aforementioned differences we then compared the mutational signature exposure across primary and metastatic tumors grouped by metastatic location (Suppl. Fig. 31).

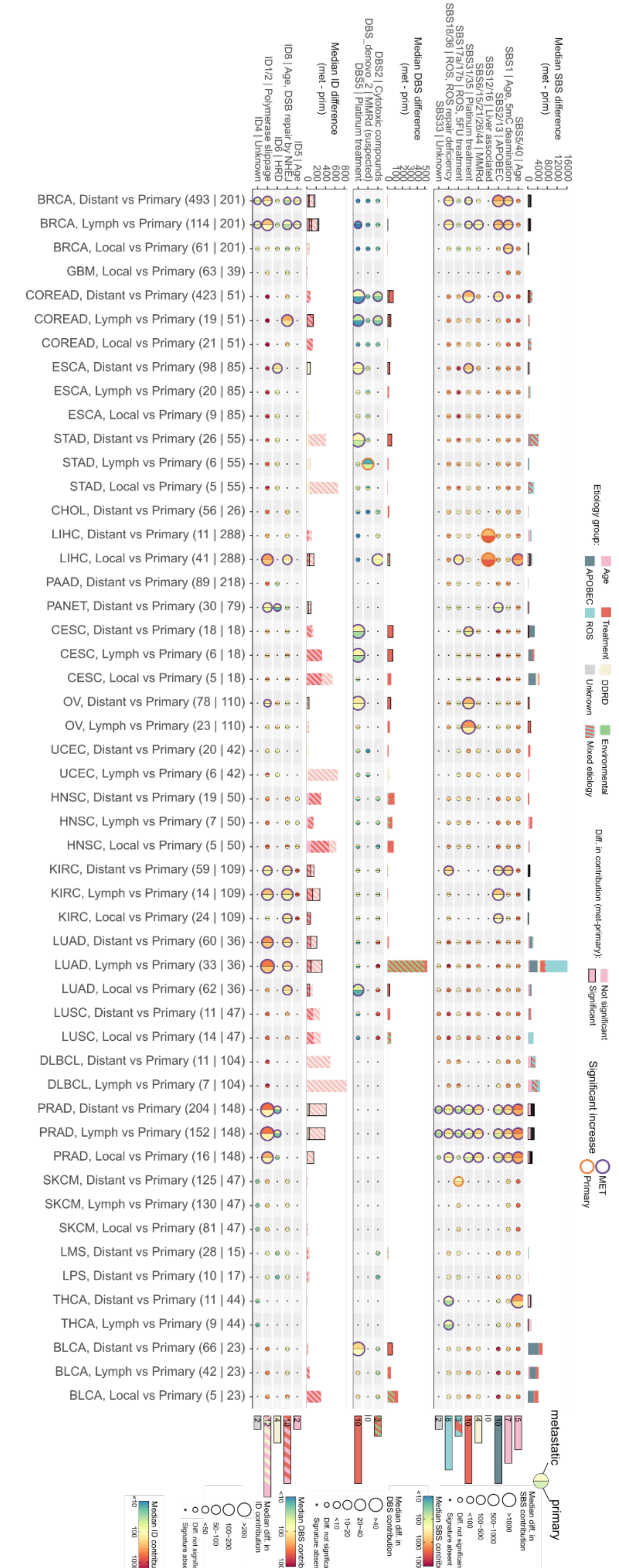

BRCA, Distant vs Primary (493 | 201)
BRCA, Lymph vs Primary (114 | 201)
BRCA, Local vs Primary (61 | 201)
GBM, Local vs Primary (63 | 39)
COREAD, Distant vs Primary (423 | 51)
COREAD, Lymph vs Primary (19 | 51)
COREAD, Local vs Primary (21 | 51)
ESCA, Distant vs Primary (98 | 85)
ESCA, Lymph vs Primary (20 | 85)
ESCA, Local vs Primary (9 | 85)
STAD, Distant vs Primary (26 | 55)
STAD, Lymph vs Primary (6 | 55)
STAD, Local vs Primary (5 | 55)
CHOL, Distant vs Primary (56 | 26)
LIHC, Distant vs Primary (11 | 288)
LIHC, Local vs Primary (41 | 288)
PAAD, Distant vs Primary (89 | 218)
PANET, Distant vs Primary (30 | 79)
CESC, Distant vs Primary (18 | 18)
CESC, Lymph vs Primary (6 | 18)
CESC, Local vs Primary (5 | 18)
OV, Distant vs Primary (78 | 110)
OV, Lymph vs Primary (23 | 110)
UCEC, Distant vs Primary (20 | 42)
UCEC, Lymph vs Primary (6 | 42)
HNSC, Distant vs Primary (19 | 50)
HNSC, Lymph vs Primary (7 | 50)
HNSC, Local vs Primary (5 | 50)
KIRC, Distant vs Primary (59 | 109)
KIRC, Lymph vs Primary (14 | 109)
KIRC, Local vs Primary (24 | 109)
LUAD, Distant vs Primary (60 | 36)
LUAD, Lymph vs Primary (33 | 36)
LUAD, Local vs Primary (62 | 36)
LUSC, Distant vs Primary (11 | 47)
LUSC, Local vs Primary (14 | 47)
DLBCL, Distant vs Primary (11 | 104)
DLBCL, Lymph vs Primary (7 | 104)
PRAD, Distant vs Primary (204 | 148)
PRAD, Lymph vs Primary (152 | 148)
PRAD, Local vs Primary (16 | 148)
SKCM, Distant vs Primary (125 | 47)
SKCM, Lymph vs Primary (130 | 47)
SKCM, Local vs Primary (81 | 47)
LMS, Distant vs Primary (28 | 15)
LPS, Distant vs Primary (10 | 17)
THCA, Distant vs Primary (11 | 44)
THCA, Lymph vs Primary (9 | 44)
BLCA, Distant vs Primary (66 | 23)
BLCA, Lymph vs Primary (42 | 23)
BLCA, Local vs Primary (5 | 23)
Median SBS difference (met - prim)
SBS5/40 | Age
SBS1 | Age, 5mC deamination
SBS2/13 | APOBEC
SBS12/16 | Liver associated
SBS6/15/21/26/44 | MMRd
SBS31/35 | Platinum treatment
SBS17a/17b | ROS, 5FU treatment
SBS18/36 | ROS, ROS repair deficiency
SBS33 | Unknown
Median DBS difference (met - prim)
DBS2 | Cytotoxic compounds
DBS_denovo_2 | MMRd (suspected)
DBS5 | Platinum treatment
Median ID difference (met - prim)
ID5 | Age
ID8 | Age, DSB repair by NHEJ
ID6 | HRD
ID1/2 | Polymerase slippage
ID4 | Unknown
Etiology group:
Age
Treatment
APOBEC
DDRD
ROS
Environmental
Unknown
Mixed etiology
Diff. in contribution (met-primary):
Not significant
Significant
Significant increase
MET
Primary
metastatic
primary
Median diff. in SBS contribution
Median diff. in DBS contribution
Median diff. in ID contribution
Median SBS contribution
Median DBS contribution
Median ID contribution

Supplementary Figure 31: Top panel, moon plot representing the mutational burden differences attributed to each mutational signature in metastatic (left) and primary (right) tumors from cancer types. For each cancer type, metastatic tumors are grouped according to biopsy location, from top to the bottom, distant metastasis, lymph node metastasis and local metastasis. Edge thickness and colors represent significant differences (two-sided Mann-Whitney adjusted p-value<0.05, ±1.4x fold change) and the direction of the enrichment, respectively. The size of circles are proportionate to the mutation burden difference. Right bars, number of metastatic cancer types with a mutational signature significant enrichment. Top stacked bars represent the cumulative signature exposure difference. Thicker bar edge lines represent significance. Bars are coloured according to the annotated etiology. Only mutational signatures with known etiology or with at least one cancer subtype with significant metastatic enrichment are included. Mid and bottom panels equivalent for DBS and IDs, respectively. Diff., difference. Muts. mutations. Sig., mutational signature. Mut. mutational. Susp., suspected. Def., deficiency.

First, the LUAD lymph node TMB increase seemed to be driven by an substantially higher exposure of tobacco induced alterations (SBS4, DBS2 and ID3), a modest contribution from platinum induced mutations (DBS5) and a strong increase in ROS induced mutations (SBS18) (see top bars). However the increase in ROS could also be caused by the high overlap of its mutational profile with SBS4 (tobacco induced mutations, cosine similarity of 0.82) (see Supp. Table 3). In fact, clinical data of patient smoking status revealed a moderate increase of smokers in LUAD LN metastases (77%=24/31), compared to other metastatic groups (Distant: 67%=39/58, Local: 66%=20/58/), which also support the notion that these differences may be driven by higher frequency of smokers among LN metastatic tumor patients. Unfortunately, patient smoking status was unavailable for primary LUAD patients. It thus seems that the TMB increase in LUAD-lymph node mets was partially explained by higher incidence of smokers within this subgroup and by a higher contribution of treatment associated mutations and indels.

According to the mutational signature attribution, the increase in SNV and DBS burden in stomach distant mets was primarily linked to an increased mutation load of SBS17a/SBS17b (linked to ROS and 5-Fluorouracil treatment) for SNVs and to increased contribution from platinum induced DBSs (DBS5), respectively. Next, the reported differences in breast cancer were primarily linked to higher APOBEC SBS2/SBS13 mutations, platinum treatment mutations (SBS31/SBS35) and higher ROS induced mutations (SBS18/SBS36) in distant mets compared to local metastatic lesions. In cervical carcinomas the most remarkable difference was higher exposure to platinum associated SNVs (SBS31/SBS35) and DBSs (DBS5) in distant mets compared to local biopsies. Finally, in prostate carcinomas the increased mutation burden in distant mets from prostate carcinomas was linked to a broad increase in multiple mutational processes exposure. Follow-up analysis, based on WGS paired biopsies, will be required to elucidate whether the reported differences in this analysis are exclusively attributed to clinical heterogeneity across metastatic tumor patients groups (i.e., different treatment regimes, higher number of chemotherapy cycles, discordant representation of smokers across metastatic locations, etc.) or due to the influence of metastatic location in damage and repair dynamics.

Structural variant burden

We then assessed whether metastatic location may also have an influence in structural variant burden across the different types of SV analyzed. Overall, we did not observe a general pattern towards enrichment/depletion of SV burden in any particular metastatic location (Suppl. Fig. 32). However, in three cancer types, namely esophageal, kidney renal clear cell and prostate carcinomas, our data revealed a moderate increase in SV burden in distant metastasis compared to local lesions, although this trend was not observed in other cancer types. Another remarkable exception was the substantial increase in SV burden in LUAD LN mets compared to other metastatic locations (i.e., distant and local). This increase may partially be explained by the exceptionally high genomic instability indicators observed in this group (see Suppl. Fig. 29 above).

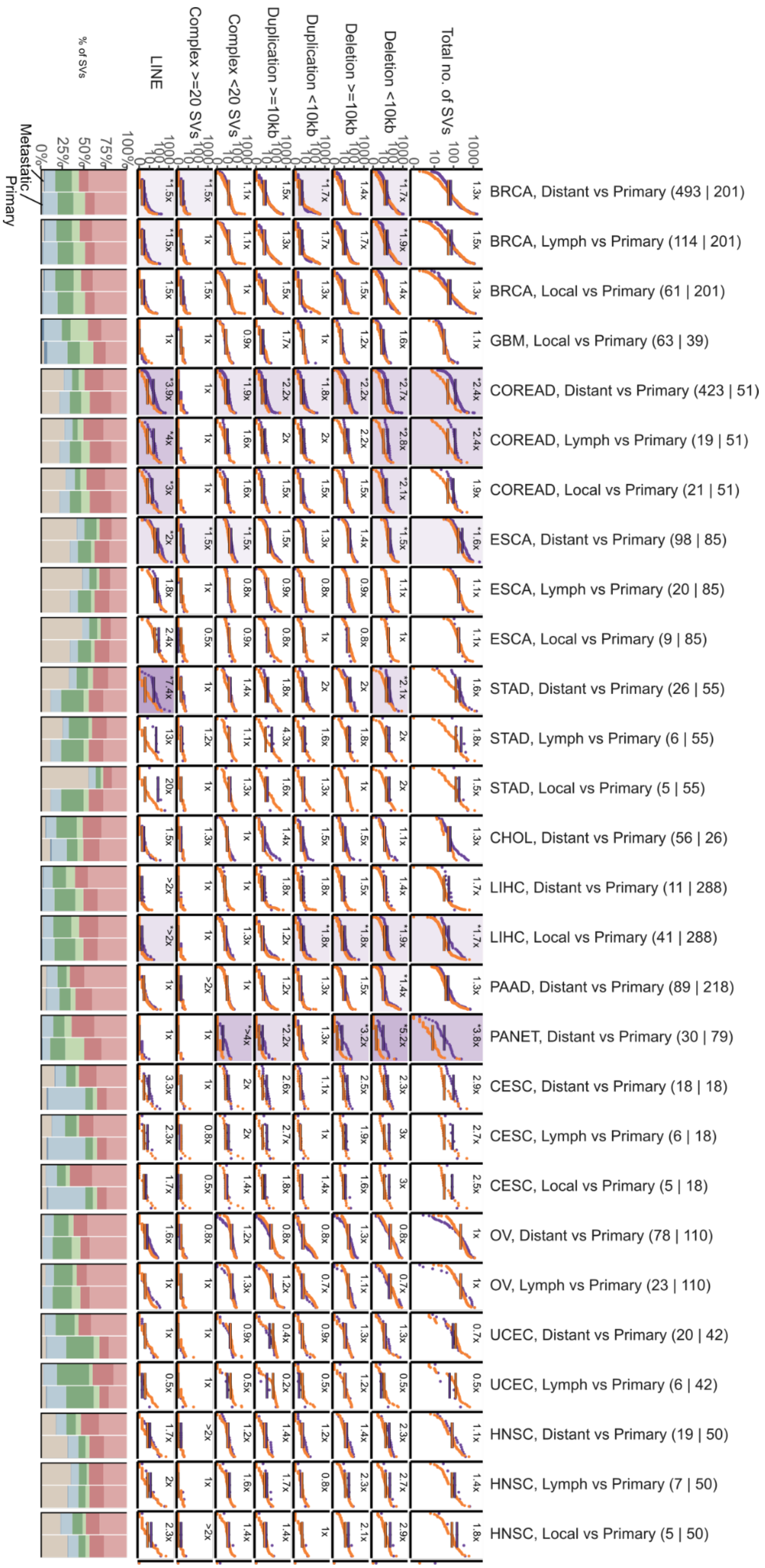
% of SVs
Metastatic
Primary
LINE
Complex >=20 SVs
Complex <20 SVs
Duplication >=10kb
Duplication <10kb
Deletion >=10kb
Deletion <10kb
Total no. of SVs
BRCA, Distant vs Primary (493 | 201)
BRCA, Lymph vs Primary (114 | 201)
BRCA, Local vs Primary (61 | 201)
GBM, Local vs Primary (63 | 39)
COREAD, Distant vs Primary (423 | 51)
COREAD, Lymph vs Primary (19 | 51)
COREAD, Local vs Primary (21 | 51)
ESCA, Distant vs Primary (98 | 85)
ESCA, Lymph vs Primary (20 | 85)
ESCA, Local vs Primary (9 | 85)
STAD, Distant vs Primary (26 | 55)
STAD, Lymph vs Primary (6 | 55)
STAD, Local vs Primary (5 | 55)
CHOL, Distant vs Primary (56 | 26)
LIHC, Distant vs Primary (11 | 288)
LIHC, Local vs Primary (41 | 288)
PAAD, Distant vs Primary (89 | 218)
PANET, Distant vs Primary (30 | 79)
CESC, Distant vs Primary (18 | 18)
CESC, Lymph vs Primary (6 | 18)
CESC, Local vs Primary (5 | 18)
OV, Distant vs Primary (78 | 110)
OV, Lymph vs Primary (23 | 110)
UCEC, Distant vs Primary (20 | 42)
UCEC, Lymph vs Primary (6 | 42)
HNSC, Distant vs Primary (19 | 50)
HNSC, Lymph vs Primary (7 | 50)
HNSC, Local vs Primary (5 | 50)

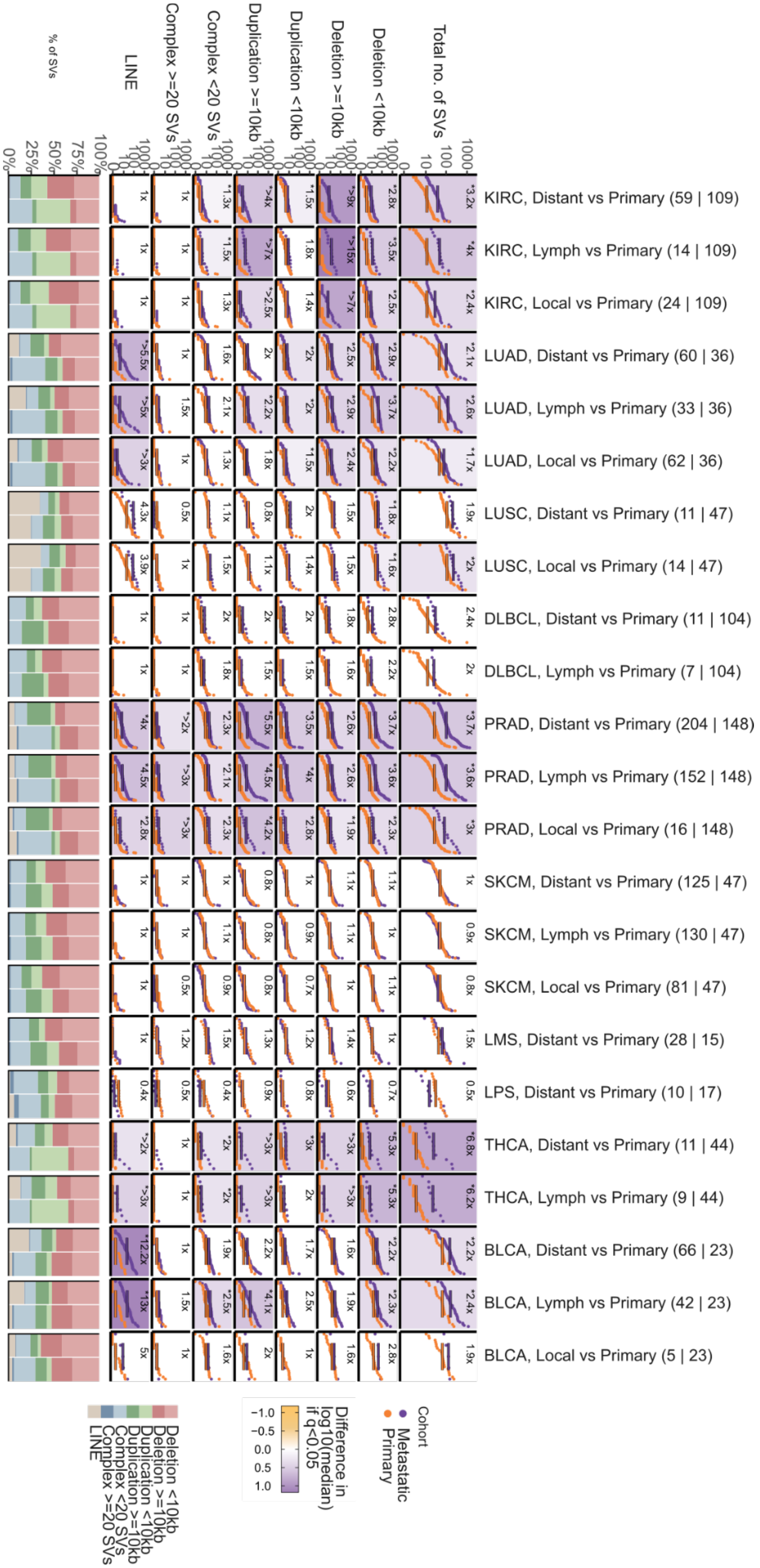
Total no. of SVs
Deletion <10kb
Deletion >=10kb
Duplication <10kb
Duplication >=10kb
Complex <20 SVs
Complex >=20 SVs
LINE
% of SVs
KIRC, Distant vs Primary (59 | 109)
KIRC, Lymph vs Primary (14 | 109)
KIRC, Local vs Primary (24 | 109)
LUAD, Distant vs Primary (60 | 36)
LUAD, Lymph vs Primary (33 | 36)
LUAD, Local vs Primary (62 | 36)
LUSC, Distant vs Primary (11 | 47)
LUSC, Local vs Primary (14 | 47)
DLBCL, Distant vs Primary (11 | 104)
DLBCL, Lymph vs Primary (7 | 104)
PRAD, Distant vs Primary (204 | 148)
PRAD, Lymph vs Primary (152 | 148)
PRAD, Local vs Primary (16 | 148)
SKCM, Distant vs Primary (125 | 47)
SKCM, Lymph vs Primary (130 | 47)
SKCM, Local vs Primary (81 | 47)
LMS, Distant vs Primary (28 | 15)
LPS, Distant vs Primary (10 | 17)
THCA, Distant vs Primary (11 | 44)
THCA, Lymph vs Primary (9 | 44)
BLCA, Distant vs Primary (66 | 23)
BLCA, Lymph vs Primary (42 | 23)
BLCA, Local vs Primary (5 | 23)
Cohort
Metastatic
Primary
Difference in log10(median) if q<0.05
Deletion <10kb
Deletion >=10kb
Duplication <10kb
Duplication >=10kb
Complex <20 SVs
Complex >=20 SVs
LINE

Supplementary Figure 32: S-plots, cumulative distribution function plot (samples ranked independently for each SV type) of tumor mutation burden for each cancer type for (from top to the bottom) the aggregated structural variant (SV) burden, small deletions (<10kb), large deletions (>=10kb), small duplications (<10kb), large duplications (>=10kb), complex events (<20 breakpoints), complex events (>=20 breakpoints) and LINEs insertions. For each cancer type, metastatic tumors are grouped according to biopsy location, from top to the bottom, distant metastasis, lymph node metastasis and local metastasis.Horizontal lines represent median values. Backgrounds are coloured according to the relative enrichment, defined as: $\log_{10}$(median SV type burden in metastatic tumors + 1) − $\log_{10}$(median SV type burden in primary tumors + 1). Coloured backgrounds and “*” are displayed when a two-sided Mann-Whitney test comparison renders a significant q-value < 0.05. Fold change labels are displayed with ‘>’ when the SV burden for primary tumors is 0 (see methods for more details). For each cancer type, bottom bar plots represent the relative fraction of each SV type in the metastatic (left) and primary (right) datasets.

Given the SV burden differences observed across breast cancer subtypes (see above) and considering the large patient representation for different breast cancer metastatic locations we also performed the analysis by breast cancer subtype.

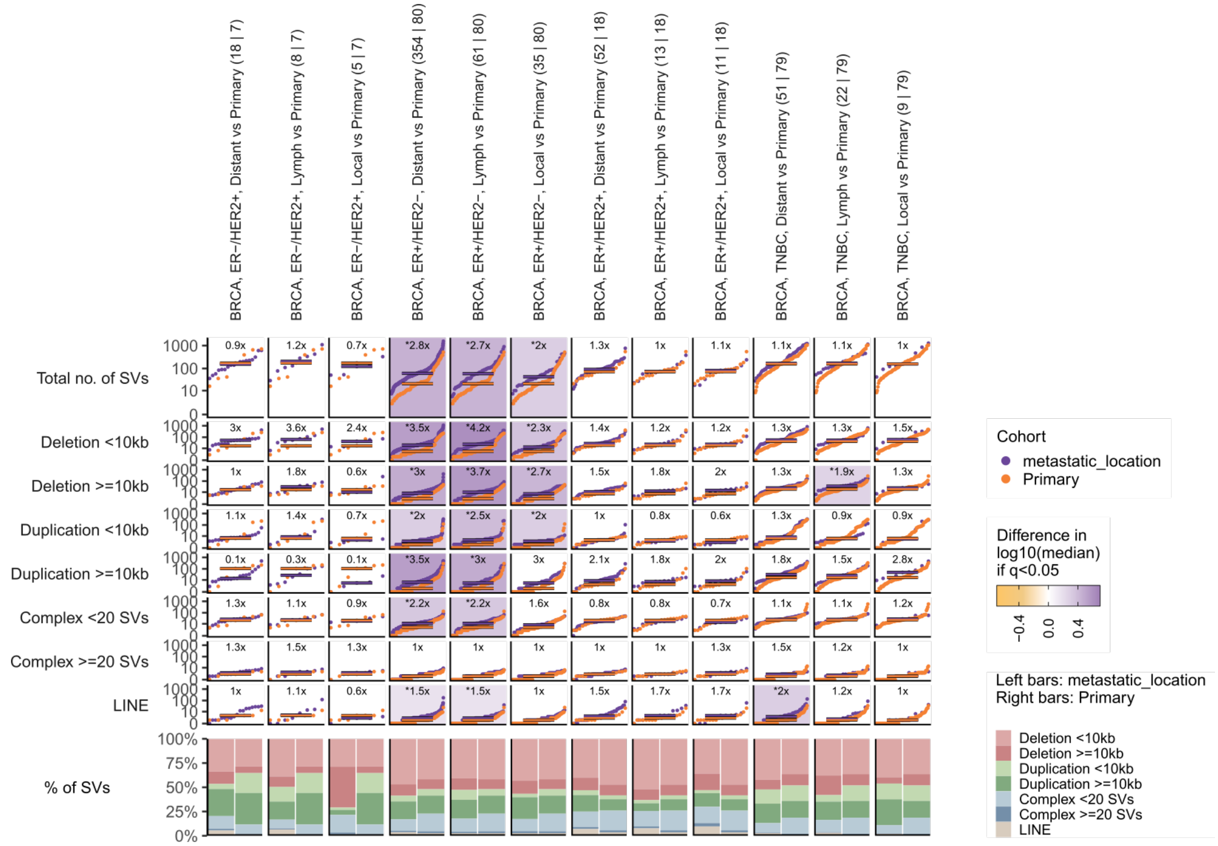

Supplementary Figure 33: S-plots, cumulative distribution function plot (samples ranked independently for each SV type) of tumor mutation burden for breast cancer subtypes for (from top to the bottom) the

aggregated structural variant (SV) burden, small deletions (<10kb), large deletions (>=10kb), small duplications (<10kb), large duplications (>=10kb), complex events (<20 breakpoints), complex events (>=20 breakpoints) and LINEs insertions. For each breast cancer subtype, metastatic tumors are grouped according to biopsy location, from left to the right, distant metastasis, lymph node metastasis and local metastasis.Horizontal lines represent median values. Backgrounds are coloured according to the relative enrichment, defined as: $\log_{10}$(median SV type burden in metastatic tumors + 1) − $\log_{10}$(median SV type burden in primary tumors + 1). Coloured backgrounds and “*” are displayed when a two-sided Mann-Whitney test comparison renders a significant q-value < 0.05. Fold change labels are displayed with ‘>’ when the SV burden for primary tumors is 0 (see methods for more details). For each cancer subtype, bottom bar plots represent the relative fraction of each SV type in the metastatic (left) and primary (right) datasets.

Consistent with the cancer type level results, we did not find any significant evidence of enrichment/depletion of SV burden across different metastatic locations of breast cancer subtypes.

Drivers

We next explored whether metastatic biopsy location may influence the driver landscape. We first evaluated whether metastatic location influences the average number of drivers per sample and how it compares to primary tumors. In general we did not observe a systemic bias towards higher/lower number of driver events for any specific metastatic location. The most remarkable difference (in agreement with previous observations, see above), is that lung adenocarcinoma (LUAD) lymph node metastasis had a significantly higher number of drivers per sample compared to LUAD primary tumors (+2.46 lymph node, +1.89 distant and +1.04 local compared to primary LUAD tumors).

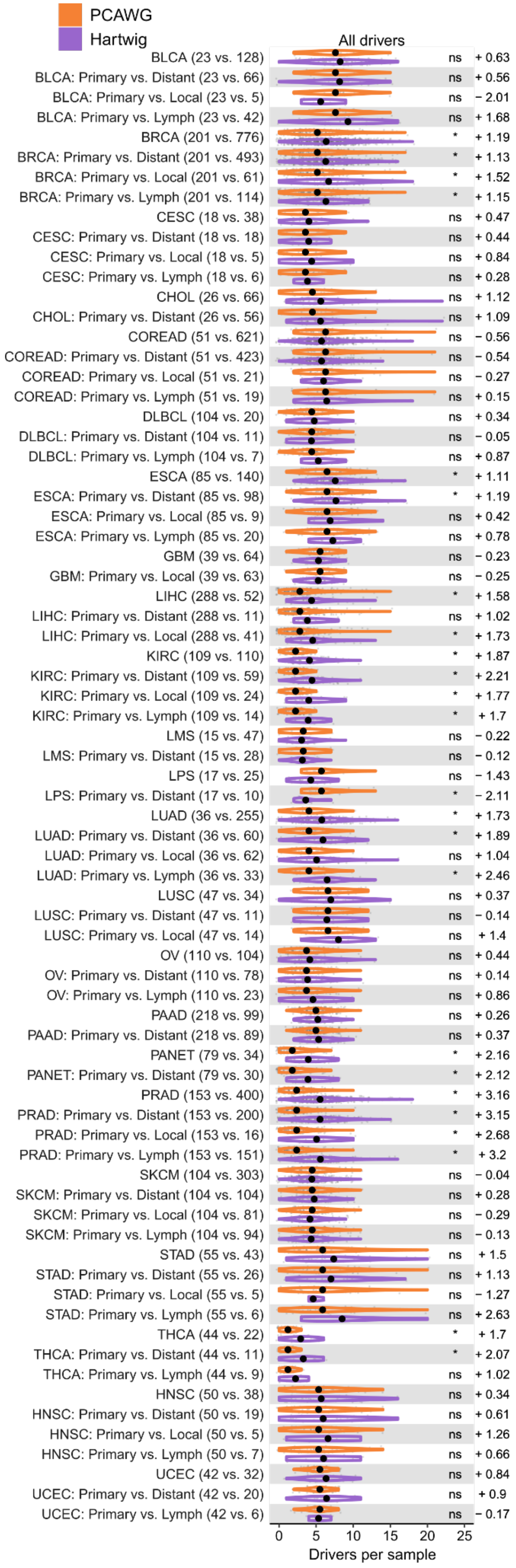
PCAWG
Hartwig
All drivers
BLCA (23 vs. 128) ns + 0.63
BLCA: Primary vs. Distant (23 vs. 66) ns + 0.56
BLCA: Primary vs. Local (23 vs. 5) ns − 2.01
BLCA: Primary vs. Lymph (23 vs. 42) ns + 1.68
BRCA (201 vs. 776) * + 1.19
BRCA: Primary vs. Distant (201 vs. 493) * + 1.13
BRCA: Primary vs. Local (201 vs. 61) * + 1.52
BRCA: Primary vs. Lymph (201 vs. 114) * + 1.15
CESC (18 vs. 38) ns + 0.47
CESC: Primary vs. Distant (18 vs. 18) ns + 0.44
CESC: Primary vs. Local (18 vs. 5) ns + 0.84
CESC: Primary vs. Lymph (18 vs. 6) ns + 0.28
CHOL (26 vs. 66) ns + 1.12
CHOL: Primary vs. Distant (26 vs. 56) ns + 1.09
COREAD (51 vs. 621) ns − 0.56
COREAD: Primary vs. Distant (51 vs. 423) ns − 0.54
COREAD: Primary vs. Local (51 vs. 21) ns − 0.27
COREAD: Primary vs. Lymph (51 vs. 19) ns + 0.15
DLBCL (104 vs. 20) ns + 0.34
DLBCL: Primary vs. Distant (104 vs. 11) ns − 0.05
DLBCL: Primary vs. Lymph (104 vs. 7) ns + 0.87
ESCA (85 vs. 140) * + 1.11
ESCA: Primary vs. Distant (85 vs. 98) * + 1.19
ESCA: Primary vs. Local (85 vs. 9) ns + 0.42
ESCA: Primary vs. Lymph (85 vs. 20) ns + 0.78
GBM (39 vs. 64) ns − 0.23
GBM: Primary vs. Local (39 vs. 63) ns − 0.25
LIHC (288 vs. 52) * + 1.58
LIHC: Primary vs. Distant (288 vs. 11) ns + 1.02
LIHC: Primary vs. Local (288 vs. 41) * + 1.73
KIRC (109 vs. 110) * + 1.87
KIRC: Primary vs. Distant (109 vs. 59) * + 2.21
KIRC: Primary vs. Local (109 vs. 24) * + 1.77
KIRC: Primary vs. Lymph (109 vs. 14) * + 1.7
LMS (15 vs. 47) ns − 0.22
LMS: Primary vs. Distant (15 vs. 28) ns − 0.12
LPS (17 vs. 25) ns − 1.43
LPS: Primary vs. Distant (17 vs. 10) * − 2.11
LUAD (36 vs. 255) * + 1.73
LUAD: Primary vs. Distant (36 vs. 60) * + 1.89
LUAD: Primary vs. Local (36 vs. 62) ns + 1.04
LUAD: Primary vs. Lymph (36 vs. 33) * + 2.46
LUSC (47 vs. 34) ns + 0.37
LUSC: Primary vs. Distant (47 vs. 11) ns − 0.14
LUSC: Primary vs. Local (47 vs. 14) ns + 1.4
OV (110 vs. 104) ns + 0.44
OV: Primary vs. Distant (110 vs. 78) ns + 0.14
OV: Primary vs. Lymph (110 vs. 23) ns + 0.86
PAAD (218 vs. 99) ns + 0.26
PAAD: Primary vs. Distant (218 vs. 89) ns + 0.37
PANET (79 vs. 34) * + 2.16
PANET: Primary vs. Distant (79 vs. 30) * + 2.12
PRAD (153 vs. 400) * + 3.16
PRAD: Primary vs. Distant (153 vs. 200) * + 3.15
PRAD: Primary vs. Local (153 vs. 16) * + 2.68
PRAD: Primary vs. Lymph (153 vs. 151) * + 3.2
SKCM (104 vs. 303) ns − 0.04
SKCM: Primary vs. Distant (104 vs. 104) ns + 0.28
SKCM: Primary vs. Local (104 vs. 81) ns − 0.29
SKCM: Primary vs. Lymph (104 vs. 94) ns − 0.13
STAD (55 vs. 43) ns + 1.5
STAD: Primary vs. Distant (55 vs. 26) ns + 1.13
STAD: Primary vs. Local (55 vs. 5) ns − 1.27
STAD: Primary vs. Lymph (55 vs. 6) ns + 2.63
THCA (44 vs. 22) * + 1.7
THCA: Primary vs. Distant (44 vs. 11) * + 2.07
THCA: Primary vs. Lymph (44 vs. 9) ns + 1.02
HNSC (50 vs. 38) ns + 0.34
HNSC: Primary vs. Distant (50 vs. 19) ns + 0.61
HNSC: Primary vs. Local (50 vs. 5) ns + 1.26
HNSC: Primary vs. Lymph (50 vs. 7) ns + 0.66
UCEC (42 vs. 32) ns + 0.84
UCEC: Primary vs. Distant (42 vs. 20) ns + 0.9
UCEC: Primary vs. Lymph (42 vs. 6) ns − 0.17
0 5 10 15 20 25
Drivers per sample

Supplementary Figure 34: Cancer type specific distribution of number of driver alterations, amplifications, deletions and mutations per patient in primary (top) and metastatic (bottom). For each cancer type, metastatic tumors are grouped according to biopsy location, from top to the bottom, grouped metastatic biopsies, distant metastasis, lymph node metastasis and local metastasis. Horizontal lines represent median values.Black dots represent the mean values. Labels display mean differences (metastatic - primary) in cancer types with a significant difference. Ns, non-significant p-value. “*”, two-sided Mann-Whitney adjusted p-value <0.01.

We next evaluated driver specific enrichment by metastatic location compared to primary tumors. Given the scope of this study and the aforementioned challenges associated with the metastatic biopsy annotations, we limited the analysis to drivers displaying significantly different mutation frequencies between primary and metastatic tumors. We refer to the study performed by Bastien Nguyen and colleagues [13] for a more comprehensive view of driver metastatic organotropism.

The table below includes driver genes showing a discrepant mutation frequency between primary and metastatic tumors and that also showed a highly heterogeneous frequency among the metastatic biopsy location groups.

Primary mutation freq. Metastatic mutation freq.

Cancer type	Gene	Cramer's V	P-value	P-value (FDR adjusted)
**Amplifications**				
Prostate carcinoma (1/153 vs. 133/400)	AR	0.34	< 0.01	< 0.01
Prostate carcinoma: Primary vs. Distant (1/153 vs. 63/200)	AR	0.40	< 0.01	< 0.01
Prostate carcinoma: Primary vs. Local (1/153 vs. 4/16)	AR	0.42	< 0.01	Not calc (min. freq < 5)
Prostate carcinoma: Primary vs. Lymph (1/153 vs. 55/151)	AR	0.46	< 0.01	< 0.01
Skin melanoma (13/104 vs. 9/303)	CCND1	-0.18	< 0.01	< 0.01
Skin melanoma: Primary vs. Distant (13/104 vs. 5/104)	CCND1	-0.14	n.s.	n.s.
Skin melanoma: Primary vs. Local (13/104 vs. 3/81)	CCND1	-0.16	< 0.05	n.s.
Skin melanoma: Primary vs. Lymph (13/104 vs. 1/94)	CCND1	-0.22	< 0.01	< 0.01
Liposarcoma (16/17 vs. 14/25)	CDK4	-0.41	< 0.05	< 0.05
Liposarcoma: Primary vs. Distant (16/17 vs. 3/10)	CDK4	-0.68	< 0.01	< 0.01
Skin melanoma (11/104 vs. 9/303)	FGF3	-0.15	< 0.01	< 0.05
Skin melanoma: Primary vs. Distant (11/104 vs. 5/104)	FGF3	-0.11	n.s.	n.s.
Skin melanoma: Primary vs. Local (11/104 vs. 3/81)	FGF3	-0.13	n.s.	n.s.
Skin melanoma: Primary vs. Lymph (11/104 vs. 1/94)	FGF3	-0.20	< 0.01	< 0.01
Liposarcoma (16/17 vs. 14/25)	MDM2	-0.41	< 0.05	< 0.05
Liposarcoma: Primary vs. Distant (16/17 vs. 3/10)	MDM2	-0.68	< 0.01	< 0.01
Prostate carcinoma (0/153 vs. 19/400)	NCOA2	0.12	< 0.01	< 0.05
Prostate carcinoma: Primary vs. Distant (0/153 vs. 12/200)	NCOA2	0.16	< 0.01	< 0.01
Prostate carcinoma: Primary vs. Local (0/153 vs. 0/16)	NCOA2	0.00	n.s.	Not calc (min. freq < 5)
Prostate carcinoma: Primary vs. Lymph (0/153 vs. 5/151)	NCOA2	0.13	< 0.05	< 0.05
Skin melanoma (11/104 vs. 5/303)	RSF1	-0.20	< 0.01	< 0.01
Skin melanoma: Primary vs. Distant (11/104 vs. 3/104)	RSF1	-0.15	< 0.05	n.s.
Skin melanoma: Primary vs. Local (11/104 vs. 1/81)	RSF1	-0.19	< 0.05	n.s.
Skin melanoma: Primary vs. Lymph (11/104 vs. 0/94)	RSF1	-0.23	< 0.01	< 0.01

Supplementary Figure 35: Cancer type specific frequency comparison of driver copy-number gains between primary and metastatic tumors. For each cancer type, metastatic tumors are grouped according to biopsy location, from top to the bottom, grouped metastatic biopsies, distant metastasis, lymph node metastasis and local metastasis. Rows highlighted with green background are discussed in the text.

Cancer type	Gene	Cramer's V	P-value	P-value (FDR adjusted)
**Deletions**				
Kidney renal clear cell carcinoma (3/109 vs. 33/110)	CDKN2A	0.37	< 0.01	< 0.01
Kidney renal clear cell carcinoma: Primary vs. Distant (3/109 vs. 22/59)	CDKN2A	0.46	< 0.01	< 0.01
Kidney renal clear cell carcinoma: Primary vs. Local (3/109 vs. 2/24)	CDKN2A	0.11	n.s.	Not calc (min. freq < 5)
Kidney renal clear cell carcinoma: Primary vs. Lymph (3/109 vs. 6/14)	CDKN2A	0.49	< 0.01	< 0.01
Ovarian serous adenocarcinoma (1/110 vs. 12/104)	CDKN2A	0.22	< 0.01	< 0.01
Ovarian serous adenocarcinoma: Primary vs. Distant (1/110 vs. 11/78)	CDKN2A	0.27	< 0.01	< 0.01
Ovarian serous adenocarcinoma: Primary vs. Lymph (1/110 vs. 1/23)	CDKN2A	0.11	n.s.	Not calc (min. freq < 5)
Pancreas neuroendocrine (3/79 vs. 8/34)	CDKN2A	0.31	< 0.01	< 0.01
Pancreas neuroendocrine: Primary vs. Distant (3/79 vs. 7/30)	CDKN2A	0.30	< 0.01	< 0.01
Breast carcinoma (13/201 vs. 13/776)	CSMD1	-0.12	< 0.01	< 0.05
Breast carcinoma: Primary vs. Distant (13/201 vs. 7/493)	CSMD1	-0.14	< 0.01	< 0.01
Breast carcinoma: Primary vs. Local (13/201 vs. 2/61)	CSMD1	-0.06	n.s.	n.s.
Breast carcinoma: Primary vs. Lymph (13/201 vs. 3/114)	CSMD1	-0.08	n.s.	n.s.
Prostate carcinoma (30/153 vs. 168/400)	PTEN	0.21	< 0.01	< 0.01
Prostate carcinoma: Primary vs. Distant (30/153 vs. 77/200)	PTEN	0.20	< 0.01	< 0.01
Prostate carcinoma: Primary vs. Local (30/153 vs. 6/16)	PTEN	0.13	n.s.	n.s.
Prostate carcinoma: Primary vs. Lymph (30/153 vs. 71/151)	PTEN	0.29	< 0.01	< 0.01
Kidney renal clear cell carcinoma (0/109 vs. 11/110)	PTPRD	0.23	< 0.01	< 0.01
Kidney renal clear cell carcinoma: Primary vs. Distant (0/109 vs. 7/59)	PTPRD	0.28	< 0.01	< 0.01
Kidney renal clear cell carcinoma: Primary vs. Local (0/109 vs. 3/24)	PTPRD	0.32	< 0.01	Not calc (min. freq < 5)
Kidney renal clear cell carcinoma: Primary vs. Lymph (0/109 vs. 0/14)	PTPRD	0.00	n.s.	Not calc (min. freq < 5)
Breast carcinoma (6/201 vs. 4/776)	RAD51B	-0.10	< 0.01	n.s.
Breast carcinoma: Primary vs. Distant (6/201 vs. 0/493)	RAD51B	-0.15	< 0.01	< 0.01
Breast carcinoma: Primary vs. Local (6/201 vs. 1/61)	RAD51B	-0.04	n.s.	n.s.
Breast carcinoma: Primary vs. Lymph (6/201 vs. 1/114)	RAD51B	-0.07	n.s.	n.s.
Prostate carcinoma (1/153 vs. 23/400)	RB1	0.11	< 0.01	n.s.
Prostate carcinoma: Primary vs. Distant (1/153 vs. 18/200)	RB1	0.18	< 0.01	< 0.01
Prostate carcinoma: Primary vs. Local (1/153 vs. 0/16)	RB1	0.02	n.s.	Not calc (min. freq < 5)
Prostate carcinoma: Primary vs. Lymph (1/153 vs. 4/151)	RB1	0.08	n.s.	Not calc (min. freq < 5)
Prostate carcinoma (3/153 vs. 61/400)	TP53	0.19	< 0.01	< 0.01
Prostate carcinoma: Primary vs. Distant (3/153 vs. 37/200)	TP53	0.26	< 0.01	< 0.01
Prostate carcinoma: Primary vs. Local (3/153 vs. 2/16)	TP53	0.18	n.s.	Not calc (min. freq < 5)
Prostate carcinoma: Primary vs. Lymph (3/153 vs. 17/151)	TP53	0.19	< 0.01	< 0.01

Supplementary Figure 36: Cancer type specific frequency comparison of driver biallelic deletions between primary and metastatic tumors. For each cancer type, metastatic tumors are grouped according to biopsy location, from top to the bottom, grouped metastatic biopsies, distant metastasis, lymph node metastasis and local metastasis. Rows highlighted with green background are discussed in the text.

Cancer type	Gene	Cramer's V	P-value	P-value (FDR adjusted)
**Fusions**				
Liposarcoma (0/17 vs. 8/25)	FUS_DDIT3	0.40	< 0.05	< 0.05
Liposarcoma: Primary vs. Distant (0/17 vs. 5/10)	FUS_DDIT3	0.62	< 0.01	< 0.01

Supplementary Figure 37: Cancer type specific frequency comparison of driver fusions between primary and metastatic tumors. For each cancer type, metastatic tumors are grouped according to biopsy location, from top to the bottom, grouped metastatic biopsies, distant metastasis.

Cancer type	Gene	Cramer's V	P-value	P-value (FDR adjusted)
**Mutations**				
Prostate carcinoma (2/153 vs. 29/400)	APC	0.12	< 0.01	n.s.
Prostate carcinoma: Primary vs. Distant (2/153 vs. 23/200)	APC	0.20	< 0.01	< 0.01
Prostate carcinoma: Primary vs. Local (2/153 vs. 0/16)	APC	0.04	n.s.	Not calc (min. freq < 5)
Prostate carcinoma: Primary vs. Lymph (2/153 vs. 6/151)	APC	0.08	n.s.	n.s.
Prostate carcinoma (1/153 vs. 73/400)	AR	0.23	< 0.01	< 0.01
Prostate carcinoma: Primary vs. Distant (1/153 vs. 39/200)	AR	0.29	< 0.01	< 0.01
Prostate carcinoma: Primary vs. Local (1/153 vs. 0/16)	AR	0.02	n.s.	Not calc (min. freq < 5)
Prostate carcinoma: Primary vs. Lymph (1/153 vs. 27/151)	AR	0.30	< 0.01	< 0.01
Pancreas neuroendocrine (0/79 vs. 5/34)	CREBBP	0.33	< 0.01	< 0.01
Pancreas neuroendocrine: Primary vs. Distant (0/79 vs. 5/30)	CREBBP	0.36	< 0.01	< 0.01
Breast carcinoma (1/201 vs. 127/776)	ESR1	0.19	< 0.01	< 0.01
Breast carcinoma: Primary vs. Distant (1/201 vs. 94/493)	ESR1	0.25	< 0.01	< 0.01
Breast carcinoma: Primary vs. Local (1/201 vs. 4/61)	ESR1	0.19	< 0.05	Not calc (min. freq < 5)
Breast carcinoma: Primary vs. Lymph (1/201 vs. 12/114)	ESR1	0.24	< 0.01	< 0.01
Ovarian serous adenocarcinoma (1/110 vs. 10/104)	KRAS	0.20	< 0.01	< 0.05
Ovarian serous adenocarcinoma: Primary vs. Distant (1/110 vs. 9/78)	KRAS	0.23	< 0.01	< 0.01
Ovarian serous adenocarcinoma: Primary vs. Lymph (1/110 vs. 1/23)	KRAS	0.11	n.s.	Not calc (min. freq < 5)
Colorectal carcinoma (19/51 vs. 92/621)	PIK3CA	-0.16	< 0.01	< 0.05
Colorectal carcinoma: Primary vs. Distant (19/51 vs. 59/423)	PIK3CA	-0.19	< 0.01	< 0.01
Colorectal carcinoma: Primary vs. Local (19/51 vs. 5/21)	PIK3CA	-0.13	n.s.	n.s.
Colorectal carcinoma: Primary vs. Lymph (19/51 vs. 2/19)	PIK3CA	-0.26	< 0.05	n.s.
Colorectal carcinoma (6/51 vs. 8/621)	POLE	-0.19	< 0.01	< 0.05
Colorectal carcinoma: Primary vs. Distant (6/51 vs. 3/423)	POLE	-0.25	< 0.01	< 0.01
Colorectal carcinoma: Primary vs. Local (6/51 vs. 1/21)	POLE	-0.11	n.s.	n.s.
Colorectal carcinoma: Primary vs. Lymph (6/51 vs. 0/19)	POLE	-0.19	n.s.	n.s.
Prostate carcinoma (1/153 vs. 28/400)	RB1	0.13	< 0.01	< 0.05
Prostate carcinoma: Primary vs. Distant (1/153 vs. 20/200)	RB1	0.20	< 0.01	< 0.01
Prostate carcinoma: Primary vs. Local (1/153 vs. 1/16)	RB1	0.15	n.s.	Not calc (min. freq < 5)
Prostate carcinoma: Primary vs. Lymph (1/153 vs. 5/151)	RB1	0.10	n.s.	n.s.
Thyroid carcinoma (0/44 vs. 5/22)	RET	0.40	< 0.01	< 0.01
Thyroid carcinoma: Primary vs. Distant (0/44 vs. 1/11)	RET	0.27	n.s.	Not calc (min. freq < 5)
Thyroid carcinoma: Primary vs. Lymph (0/44 vs. 3/9)	RET	0.54	< 0.01	Not calc (min. freq < 5)
Hepatocellular carcinoma (21/288 vs. 30/52)	TERT	0.51	< 0.01	< 0.01
Hepatocellular carcinoma: Primary vs. Distant (21/288 vs. 5/11)	TERT	0.25	< 0.01	< 0.05
Hepatocellular carcinoma: Primary vs. Local (21/288 vs. 25/41)	TERT	0.51	< 0.01	< 0.01
Kidney renal clear cell carcinoma (3/109 vs. 19/110)	TERT	0.24	< 0.01	< 0.01
Kidney renal clear cell carcinoma: Primary vs. Distant (3/109 vs. 11/59)	TERT	0.27	< 0.01	< 0.01
Kidney renal clear cell carcinoma: Primary vs. Local (3/109 vs. 4/24)	TERT	0.24	< 0.05	Not calc (min. freq < 5)
Kidney renal clear cell carcinoma: Primary vs. Lymph (3/109 vs. 4/14)	TERT	0.35	< 0.01	Not calc (min. freq < 5)
Liposarcoma (0/17 vs. 5/25)	TERT	0.30	n.s.	n.s.
Liposarcoma: Primary vs. Distant (0/17 vs. 5/10)	TERT	0.62	< 0.01	< 0.01
Skin melanoma (47/104 vs. 214/303)	TERT	0.23	< 0.01	< 0.01
Skin melanoma: Primary vs. Distant (47/104 vs. 68/104)	TERT	0.20	< 0.01	n.s.
Skin melanoma: Primary vs. Local (47/104 vs. 60/81)	TERT	0.29	< 0.01	< 0.01
Skin melanoma: Primary vs. Lymph (47/104 vs. 68/94)	TERT	0.27	< 0.01	< 0.01
Kidney renal clear cell carcinoma (4/109 vs. 21/110)	TP53	0.24	< 0.01	< 0.01
Kidney renal clear cell carcinoma: Primary vs. Distant (4/109 vs. 9/59)	TP53	0.21	< 0.05	n.s.
Kidney renal clear cell carcinoma: Primary vs. Local (4/109 vs. 5/24)	TP53	0.26	< 0.01	n.s.
Kidney renal clear cell carcinoma: Primary vs. Lymph (4/109 vs. 4/14)	TP53	0.32	< 0.01	Not calc (min. freq < 5)
Lung adenocarcinoma (13/36 vs. 165/255)	TP53	0.19	< 0.01	n.s.
Lung adenocarcinoma: Primary vs. Distant (13/36 vs. 38/60)	TP53	0.26	< 0.05	n.s.
Lung adenocarcinoma: Primary vs. Local (13/36 vs. 37/62)	TP53	0.23	< 0.05	n.s.
Lung adenocarcinoma: Primary vs. Lymph (13/36 vs. 28/33)	TP53	0.50	< 0.01	< 0.01
Ovarian serous adenocarcinoma (108/110 vs. 78/104)	TP53	-0.34	< 0.01	< 0.01
Ovarian serous adenocarcinoma: Primary vs. Distant (108/110 vs. 57/78)	TP53	-0.38	< 0.01	< 0.01
Ovarian serous adenocarcinoma: Primary vs. Lymph (108/110 vs. 18/23)	TP53	-0.34	< 0.01	< 0.05
Prostate carcinoma (11/153 vs. 161/400)	TP53	0.32	< 0.01	< 0.01
Prostate carcinoma: Primary vs. Distant (11/153 vs. 74/200)	TP53	0.35	< 0.01	< 0.01
Prostate carcinoma: Primary vs. Local (11/153 vs. 6/16)	TP53	0.30	< 0.01	< 0.01
Prostate carcinoma: Primary vs. Lymph (11/153 vs. 69/151)	TP53	0.44	< 0.01	< 0.01
Stomach carcinoma (26/55 vs. 36/43)	TP53	0.38	< 0.01	< 0.01
Stomach carcinoma: Primary vs. Distant (26/55 vs. 22/26)	TP53	0.35	< 0.01	< 0.01
Stomach carcinoma: Primary vs. Local (26/55 vs. 4/5)	TP53	0.18	n.s.	n.s.
Stomach carcinoma: Primary vs. Lymph (26/55 vs. 5/6)	TP53	0.21	n.s.	n.s.
Thyroid carcinoma (0/44 vs. 6/22)	TP53	0.45	< 0.01	< 0.01
Thyroid carcinoma: Primary vs. Distant (0/44 vs. 3/11)	TP53	0.48	< 0.01	Not calc (min. freq < 5)
Thyroid carcinoma: Primary vs. Lymph (0/44 vs. 3/9)	TP53	0.54	< 0.01	Not calc (min. freq < 5)
Pancreas neuroendocrine (2/79 vs. 11/34)	TSC2	0.43	< 0.01	< 0.01
Pancreas neuroendocrine: Primary vs. Distant (2/79 vs. 9/30)	TSC2	0.41	< 0.01	< 0.01

Supplementary Figure 38: Cancer type specific frequency comparison of driver mutated genes between primary and metastatic tumors. For each cancer type, metastatic tumors are grouped according to biopsy location, from top to the bottom, grouped met. biopsies, distant metastasis, lymph node metastasis and local metastasis. Rows highlighted with green background are discussed in the text.

Given the dimension of the data, we will focus the following discussion in the most prominent cases, defined as the cases with **i)** significant enrichment/depletion alteration frequency in metastatic compared to primary, **ii)** substantial different alteration frequency across metastatic location, **iii)** the sample representation in the metastatic locations from **ii)** should be sufficiently higher to draw a minimally meaningful conclusion (at least 10 samples). The discussed cases are highlighted with a green shade.

First, *NCOA2* amplification was significantly enriched in metastatic prostate cancer patients compared to primaries (0/153 amplifications in primary, 19/400 (5%) in metastasis). However, this increased frequency is exclusively associated with distant and LN metastatic biopsies (6% and 3%, respectively), but not with local prostate cancer metastatic tumors (0/16 amplifications). A similar pattern was observed for *CDKN2A* deletions in kidney renal clear cell carcinoma, *RB1* deletions in prostate cancer, *APC* mutations in prostate and *AR* activating mutations in prostate. Interestingly, in spite of the global metastatic enrichment of *PTPRD* deletions in kidney renal clear cell carcinoma (0/109 in primary and 11/110 in metastasis), *PTPRD* deletions were never observed in LN metastasis (0/14). Finally, as reported above (see genomic instability), *TP53* mutations were enriched in LUAD LN metastasis (28/33, 83%) compared to primary LUAD tumors (13/36, 36%). This enrichment was in turn higher than the enrichment for other metastatic locations (37/62, 59% for local mets and 38/60, 60% for distant mets).

Conclusion

This analysis, relying on unpaired biopsies from independent patients, aimed to elucidate the influence of metastatic location in the genomic landscape of metastatic tumors. In general terms, our data suggests that, except for the tumor clonality, the genomic landscape of metastatic tumors is conserved across different metastatic biopsy locations and that the reported differences with primary tumors were metastatic location-independent.

The most remarkable exception to this trend was lung adenocarcinoma (LUAD) LN metastasis, which showed an increase in genomic instability, TMB, SV burden and certain differences in their driver landscape compared to both primary tumors and to other metastatic locations. Importantly, in spite of the extensive changes in their genomic landscape, lymph LUAD metastasis had the LUAD characteristic tumor karyotype, supporting the notion that the tumor karyotype is fixed early in tumor evolution. Nonetheless, independent studies are required to rule out that this observation was exclusively linked to a singular clinical (e.g., treatment regime) and/or life-style (e.g., smoking status) characteristic of LUAD metastatic tumor patients with LN biopsies.

A major limitation of this analysis is the high level of metastatic-location aggregation used. A more refined metastatic location annotation could reveal certain specificities that are otherwise missed when grouping multiple heterogeneous mets into the same group (e.g., different target tissues grouped into the distant groups, grouped locoregional and distant LN biopsies, etc.). This is particularly relevant for the driver landscape analysis. Hence, we refer to the study performed by Bastien Nguyen and colleagues[13] for a more comprehensive view of patterns of driver organotropism.

Supplementary Note 3

Introduction	73
Ploidy correction	73
Clonality of mutations	74
Breast carcinoma subtypes	75
Differences across different metastatic biopsy location	76
SBS5/SBS40 mutational processes comparison	77
SBS1 mutation burden in metastatic prostate samples from the PCAWG cohort	77
Clonality and timing of SBS1 mutations	78
Clonality and timing of SBS5/SBS40 mutations	81
Independent datasets description	83
Independent validation in using paired biopsies	84
Breast cancer dataset (Lucy R Yates et al.)	84
Kidney renal clear cell carcinoma dataset (TRACERx renal dataset)	87
Independent validation using external primary and metastatic datasets	90
Breast carcinoma	90
Prostate carcinoma	91
Kidney renal clear cell carcinoma	94
Thyroid carcinoma	95
Colorectal and ovarian serous carcinomas	96
Conclusions	98
Appendix 1: Exome-only mutations in Hartwig dataset	98

Introduction

This Supplementary Note contains the detailed results and a thorough description of the methodology of the validation performed for our observation of an age-independent increase of SBS1 mutation rate in metastatic breast, prostate, kidney renal clear cell and thyroid tumors.

First, we will focus on internal controls that aim to rule out underlying factors such as the tumor genome ploidy, breast tumor subtype and metastatic location as potential confounding factors to our observations. Moreover, we will assess whether this observation holds for other cell division independent and age associated mutational processes such as SBS5/SBS40.

Following with data from our cohorts, we will also look at whether the relative timing and clonality of SBS1 mutations support an increased accumulation of late/subclonal SBS1 mutations in the aforementioned cancer types. Importantly, such a pattern should not be observed for cancer types lacking an increased SBS1 mutation rate (e.g., colorectal and ovarian serous carcinomas).

Then, to further validate our observations, we decided to leverage independent and publicly available WGS/WES primary and/or metastatic datasets of these cancer types. Ideally, we will prioritize datasets including paired primary and metastatic biopsies that properly match our cohorts clinical characteristics. This means that primary biopsies should have been performed at early tumor stages, ideally at the time of diagnosis. Moreover, as mentioned above, we will necessitate high quality WGS or WES sequencing data from fresh frozen samples (i.e., excluding FFPE samples) due to the importance of rigorous identification of somatic mutations, particularly in CpG contexts.

Finally, due to the scarcity of accessible primary-met paired datasets that fulfilled the aforementioned criteria, we will also perform independent validations relying on publicly available unpaired cohorts of primary and/or metastatic tumors.

Ploidy correction

One obvious confounding factor for the increase in SBS1 mutation burden could be attributed to an increased genome ploidy of the metastatic tumors as these tumors have higher DNA content to accumulate more mutations. Therefore, we normalized the SBS1 burden by the total genome ploidy by dividing the SBS1 mutation count by the estimated average tumor ploidy of each sample.

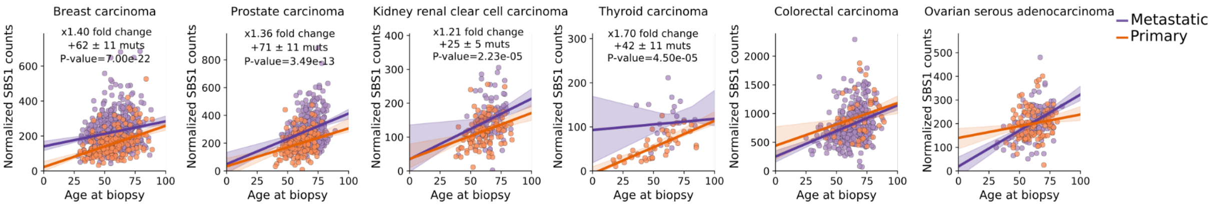

Supplementary Figure 39: Linear regression of the ploidy adjusted SBS1 mutation burden (y-axis) and patient’s age at biopsy (x-axis) in primary (orange) and metastatic tumors (purple) across the cancer types of interest (tumor types names at the top of each chart). The mean fold change, mean SBS1 increase per year and one-sided Mann-Whitney p-value are included in cancer types with an age-independent significantly different primary and metastatic distribution. The median trendline and 99% confidence intervals of the linear regression are represented as a solid line and the adjacent shaded area, respectively.

After genome ploidy normalization we still observed an age-independent and significant SBS1 increase in the five cancer types (see Suppl. Fig. 39 above and Extended Data Fig. 4c). This suggests that the increased tumor genome ploidy is not the main underlying factor behind the SBS1 mutation burden increase.

Clonality of mutations

The overall lower sequencing depth of the PCAWG primary dataset has a small influence in the subclonal mutation detection sensitivity (see Supp. Note 1 above for further details). To rule out that this effect may have an impact in the age-independent SBS1 burden differences, we repeated the age-SBS1 burden correlations comparison relying on SBS1 clonal mutations for both primary (PCAWG) and metastatic (Hartwig) datasets, as clonal mutations are, in principle, less affected by differences in sequencing depth (see Supp. Note 1).

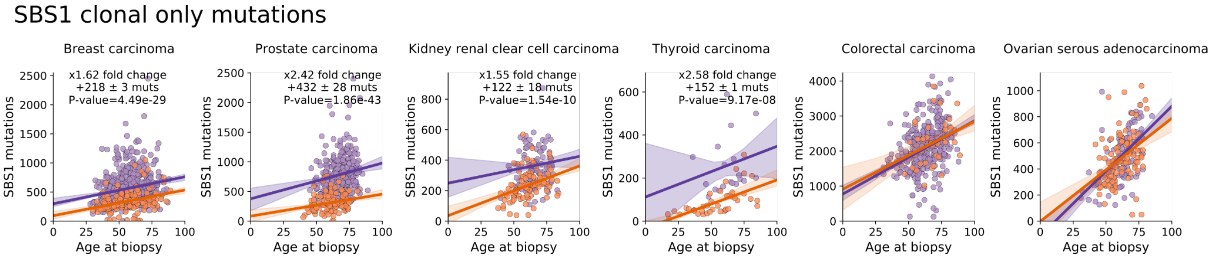

Supplementary Figure 40: Linear regression of the clonal-only SBS1 mutation burden (y-axis) and patient’s age at biopsy (x-axis) in primary (orange) and metastatic tumors (purple) across the cancer types of interest (tumor types names at the top of each chart). The mean fold change, mean SBS1 increase per year and one-sided Mann-Whitney p-value are included in cancer types with an age-independent significantly different primary and metastatic distribution. The median trendline and 99% confidence intervals of the linear regression are represented as a solid line and the adjacent shaded area, respectively.

As illustrated in Suppl. Fig. 40, The comparison revealed highly consistent patterns between the SBS1 clonal only and the SBS1 total burden, indicating that the differences in sequencing coverage are not a major confounding factor for the observed distributions.

Breast carcinoma subtypes

Another of the aspects that could potentially explain the observed differences may be attributed to an enrichment in a specific cancer subtype in the metastatic setting, which could be responsible

for the observed SBS1 mutation rate increase. This is particularly relevant for breast cancer, given the known clinical and genomic differences across subtypes and also the high sample representation in both primary and metastatic tumors. We thus annotated breast cancer subtypes (see Supp. Note 2) for primary and metastatic samples and performed a breast cancer subtype comparison.

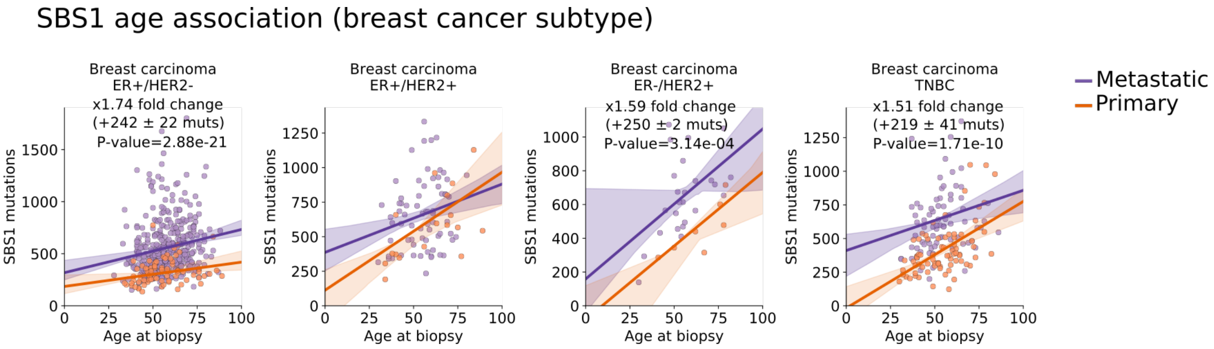

Supplementary Figure 41: Linear regression of the SBS1 mutation burden (y-axis) and patient’s age at biopsy (x-axis) in primary (orange) and metastatic tumors (purple) across the breast cancer subtypes. The mean fold change, mean SBS1 increase per year and one-sided Mann-Whitney p-value are included in breast cancer subtypes with an age-independent significantly different primary and metastatic distribution. The median trendline and 99% confidence intervals of the linear regression are represented as a solid line and the adjacent shaded area, respectively.

Our results revealed that three subtypes (i.e., ER+/HER2-, ER-/HER2+ and triple negative breast cancer) showed an age-independent SBS1 mutation burden increase (see Suppl. Fig. 41 above, now part of Extended Data Fig. 4b). The only exception was ER+/HER2+ breast cancer which did not show a clear enrichment in metastatic samples. Overall, this indicates that observed global enrichment is not driven by a unique cancer subtype but it also highlights some differences across subtypes. The most remarkable difference was that metastatic ER+/HER2+ tumors did not display a strong SBS1 mutation rate increase compared to their primary counterpairs, although given the low number of samples and their narrow age-distribution we can not formally exclude insufficient statistical power for this subtype.

Differences across different metastatic biopsy location

We also checked whether metastatic biopsy location may influence the increase in SBS1 mutation burden across the four cancer types of interest (i.e., breast, prostate, kidney and thyroid carcinomas) and the respective controls (i.e., colorectal and ovarian serous carcinomas). More specifically, we grouped the metastatic samples into three categories according to the metastatic biopsy type (i.e., local biopsy, lymph node biopsy and distant metastatic biopsy, see Supp. Table 1 and Supp. Note 2 for more information).

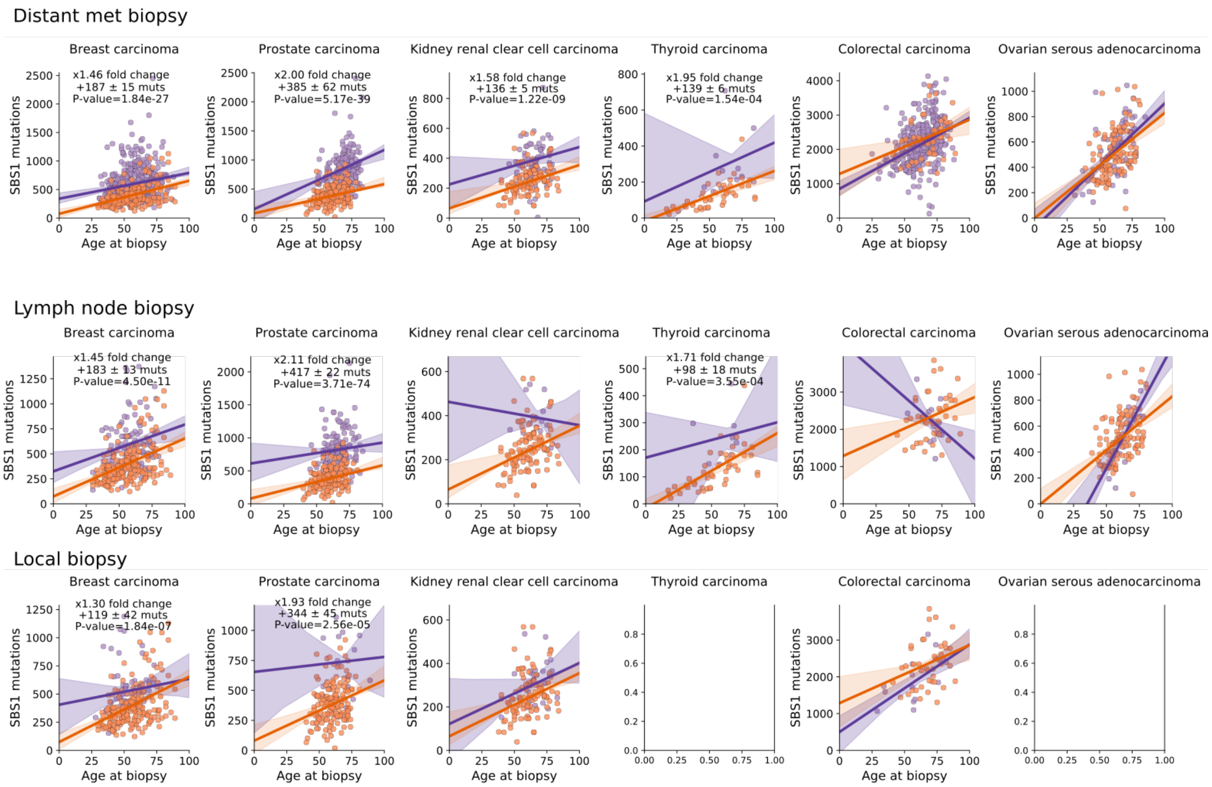

Supplementary Figure 42: Linear regression of the SBS1 mutation burden (y-axis) and patient’s age at biopsy (x-axis) in primary (orange) and metastatic tumors (purple) grouped according to their metastatic biopsy location across the cancer types of interests. Top, metastatic tumor biopsies from distant metastasis. Middle, metastatic lymph node biopsies. Bottom, metastatic tumor local biopsies. Empty charts are depicted for those cancer type and metastatic biopsy locations combinations with less than five metastatic tumor samples. The mean fold change, mean SBS1 increase per year and one-sided Mann-Whitney p-value are included in cancer types and biopsy locations with an age-independent significantly different primary and metastatic distribution. The median trendline and 99% confidence intervals of the linear regression are represented as a solid line and the adjacent shaded area, respectively.

Overall, the four cancer types showed a trend towards age-independent SBS1 mutation burden increase across the three types of metastatic location (Suppl. Fig. 42). Moreover, we did not observe any difference across primary-met age-corrected SBS1 burden in the control groups. Colorectal lymph and kidney renal clear cell carcinoma node mets show an unexpected pattern of SBS1-age association, that is most likely explained by the narrow age distribution on top of the low sample sizes.

Hence, our results suggest that the age-corrected increase in SBS1 burden is generally observed across multiple biopsy sites.

SBS5/SBS40 mutational processes comparison

We also evaluated whether this age-independent increase in mutation burden was observed for SBS5/SBS40 clock-like mutational process, whose mutation burden is correlated with patient’s age in normal and cancer cells in a cell-cycle independent manner [14–16].

Our data revealed that, after correcting by tumor genome ploidy, SBS5/SBS40 mutations did not show an age-independent mutation rate increase in metastatic tumors compared to primaries across the cancer types of interest (breast, prostate, kidney renal clear cell and thyroid carcinomas, see Suppl. Fig. 43 below). This is clearly opposed to the trend observed using SBS1 mutations, which supports the notion that different mutation mechanisms underlie both clock-like mutational processes.

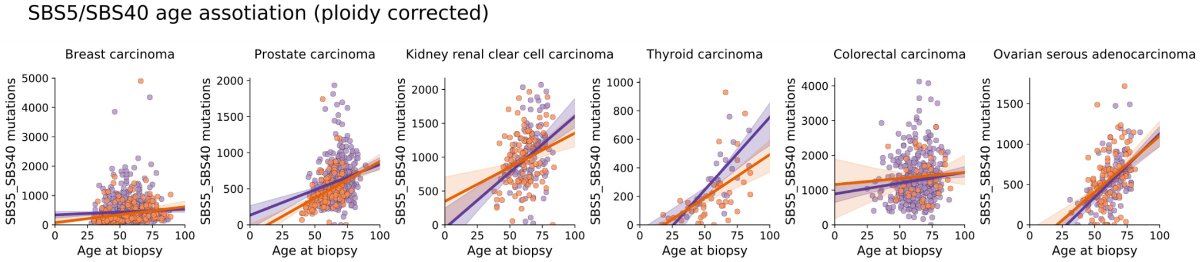

Supplementary Figure 43: Linear regression of the SBS5 and SBS40 combined mutation burden (y-axis) and patient’s age at biopsy (x-axis) in primary (orange) and metastatic tumors (purple) across the cancer types of interest. The median trendline and 99% confidence intervals of the linear regression are represented as a solid line and the adjacent shaded area, respectively.

SBS1 mutation burden in metastatic prostate samples from the PCAWG cohort

We realized that there were a few metastatic prostate biopsies in the PCAWG cohort (see Supp. Table 1). Given the fact that prostate cancer displayed a strong SBS1 burden enrichment in metastasis compared to primary, we questioned whether these tumor biopsies also displayed an age-independent SBS1 enrichment that would fit within our observations using metastatic prostate samples from the Hartwig cohort. Since these five prostate PCAWG samples have been sequenced following similar technical protocols (i.e., following the PCAWG guidelines), we believe this would also help to elucidate whether our observations are consistent after controlling by the sequencing platform and parameters.

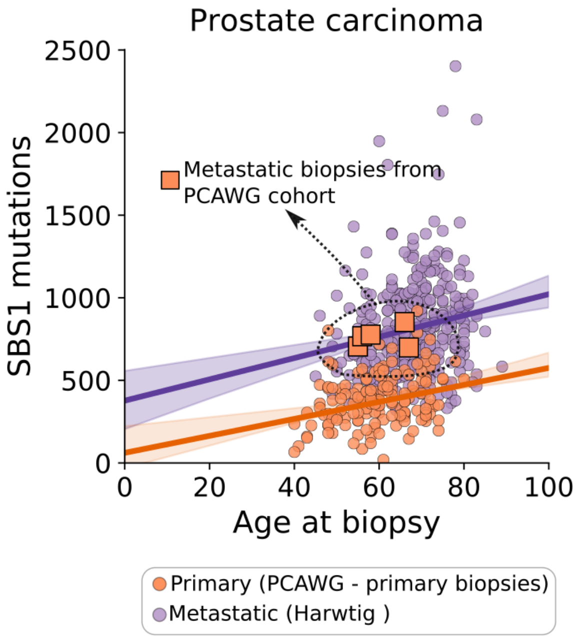

Supplementary Figure 44: Linear regression of the SBS1 mutation burden (y-axis) and patient’s age at biopsy (x-axis) in primary (orange) and metastatic (purple) prostate carcinoma tumors. Orange dots represent primary tumor samples from the PCAWG cohort, orange squares represent metastatic biopsies from the PCAWG cohort and purple dots metastatic tumor samples from Hartwig. The median trendline and 99% confidence intervals of the linear regression are represented as a solid line and the adjacent shaded area, respectively.

Interestingly, our analysis showed that all five metastatic prostate cancer biopsies included in the PCAWG cohort showed a high SBS1 burden that perfectly aligned with the expected number of SBS1 mutations given by the metastatic regression fitted with Hartwig metastatic samples (see Suppl. Fig. 44 above). This result suggests that the SBS1 mutation burden differences do not seem to be driven by differences in sequencing protocols and that are genuinely attributed to different mutation rates between primary and metastatic tumors.

Clonality and timing of SBS1 mutations

If the age-corrected increase in SBS1 mutation burden is caused by an accelerated SBS1 mutation rate in late tumorigenic stages, this should leave a traceable footprint of enrichment of late and subclonal SBS1 mutations in metastatic tumors compared to their primary counterparts. We thus compared the normalized fraction of SBS1 clonal mutations between primary and metastatic tumors across the cancer types of interest (see methods).

$$\text{SBS1 clonality ratio} = \frac{\text{Prop. SBS1 clonal mutations}}{\text{Prop. clonal mutations tumor}} \qquad \text{Prop. SBS1 clonal mutations} = \frac{\text{\#SBS1 clonal}}{\text{\#SBS1 clonal + subclonal}}$$

Our data showed that metastatic breast, prostate and thyroid metastatic tumors had an overall lower normalized fraction of clonal SBS1 mutations (Suppl. Fig. 45 below). This means SBS1 mutations were relatively less clonal in metastatic tumors than in primaries after correcting by sample-specific tumor clonality. Note that this trend was not observed for colorectal and ovarian serous carcinomas that lacked the age-independent metastatic enrichment of SBS1 mutations

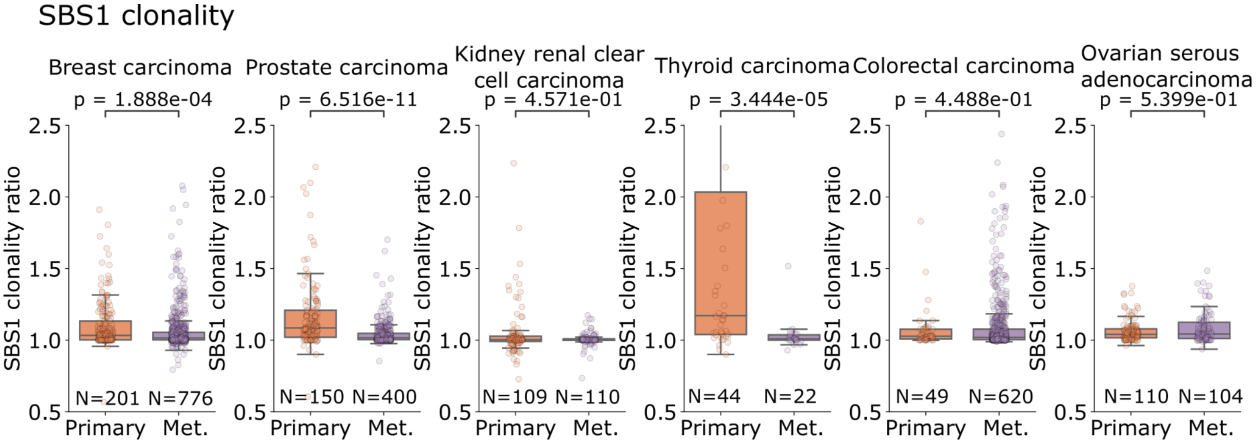

Supplementary Figure 45: Comparison of SBS1 clonality ratios between primary and metastatic tumors across the cancer type of interest. p, two-sided Mann-Whitney p-value. Box-plots: center line, median; box limits, first and third quartiles; whiskers, lowest/highest data points at first quartile minus/plus 1.5× IQR.N, number of samples.

Moreover, we reasoned that metastatic tumors should be enriched in clonal late SBS1 mutations compared to primary tumors after correcting by differences in global tumor mutation clonality and timing. We used the MutationTimeR(v0.1)[17] tool to estimate the relative timing (clonal early, clonal late or clonal unassigned) of SBS1 mutations across primary and metastatic tumors. Then, for each sample, the SBS1 clonal late ratio was defined as as the ratio between the proportion of clonal late SBS1 mutations divided by the total proportion of clonal late alterations in the sample (see below), where the total of clonal SBS1/total mutations was computed as the sum of clonal late, clonal early and clonal unassigned clonal from MutationTimerR.

$$\text{SBS1 clonal late ratio} = \frac{\text{Prop. SBS1 clonal late mutations}}{\text{Prop. clonal late mutations tumor}} \qquad \text{Prop. SBS1 clonal late mutations} = \frac{\text{\#SBS1 clonal late}}{\text{\#SBS1 clonal late + early}}$$

We observed a significant relative enrichment in clonal late SBS1 mutations in metastatic breast, prostate and kidney renal clear cell carcinomas compared to primaries (Suppl. Fig. 46). This enrichment was not observed for the control cancer types.

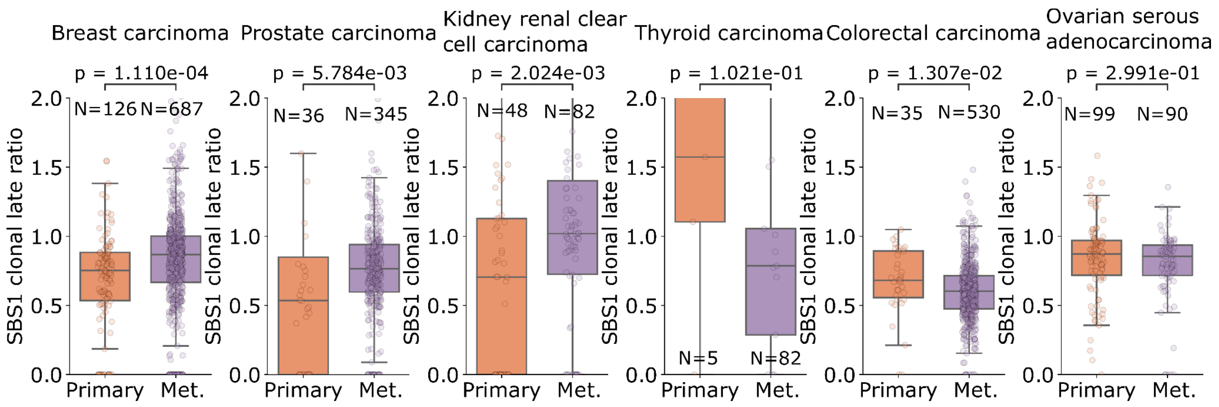

Supplementary Figure 46: Comparison of the global SBS1 clonal late ratios between primary and metastatic tumors across the cancer type of interest. p, Mann-Whitney two-sided p-value. Box-plots: center line, median; box limits, first and third quartiles; whiskers, lowest/highest data points at first quartile minus/plus 1.5× IQR. Boxplots are defined as in Suppl. Fig. 45. N, number of samples.

However, it is important to mention that assignment of clonal-early and clonal-late mutations could only be done in copy-number amplified regions. This means that this difference could also -at least partially- be driven by the limited number of amplified regions in the primary tumors (particularly in nearly fully diploid primary tumors such as thyroid and prostate carcinomas). To address this issue we also performed a comparison relying on SBS1 mutations in amplified regions lacking minor allele ploidy (*i.e.,* LOH regions in at least 2n region) or where minor allele ploidy is equal or larger than two (e.g., 2+0, 2+2, etc). Only samples with at least five clonal early/late annotated SBS1 mutations in amplified regions were considered. Moreover, cancer types where any of the cohorts (primary or metastatic) included less than five samples were not analyzed.

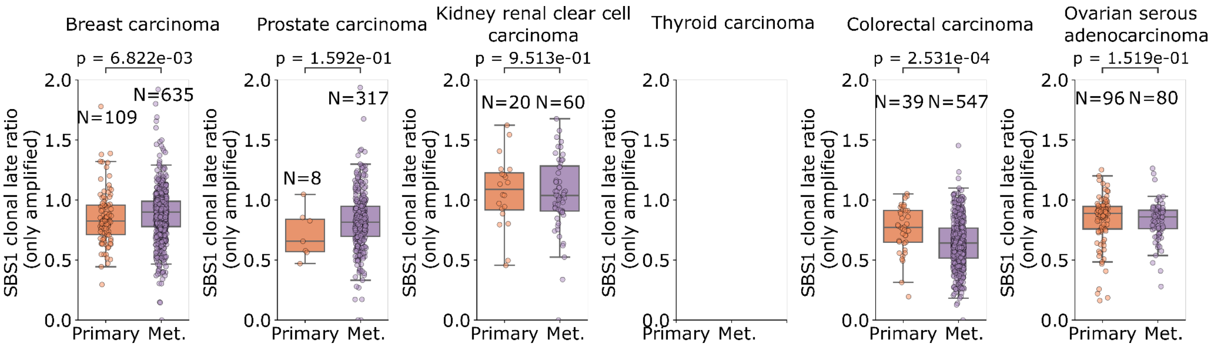

Supplementary Figure 47: Comparison of the SBS1 clonal late ratios for mutations in copy-number amplified regions between primary and metastatic tumors across the cancer type of interest. Empty charts represent those cancer types lacking sufficient data in either primary or metastatic cohorts. Boxplots are defined as in Suppl. Fig. 45. P, two-sided Mann-Whitney p-value. N, number of samples.

After this correction, breast carcinoma still showed a strong significant enrichment in clonal late SBS1 mutations in metastasis compared to primaries (Suppl. Fig. 47). However, the significance is no longer present for prostate and kidney renal clear cell carcinomas, although a modest median increase was still observed in prostate carcinoma patients. Thyroid carcinomas could not be analyzed owing to the limited number of primary samples with sufficient mutations in amplified regions. Finally, colorectal and ovarian serous carcinomas did not show an relative increase in SBS1 clonal late ratio.

In summary, our analysis revealed that the metastatic tumors displaying an age-independent SBS1 mutation burden increase showed a relative depletion in clonal mutation burden compared to primary tumors. Moreover, our data also suggests that these metastatic tumors tended to show higher rates of SBS1 clonal late mutations, although this could only be robustly assessed for breast carcinomas, given the limited number of SBS1 mutations with defined timing in primary tumors of prostate, kidney clear cell and thyroid carcinomas.

Clonality and timing of SBS5/SBS40 mutations

We next checked whether the increased SBS1 mutation rate in later tumorigenic stages was also noticeable for other clock-like mutational processes operating during the evolutionary story of the somatic tissues. Specifically, we evaluated whether the SBS5/SBS40 clonality and timing patterns were in agreement with our observation for SBS1 mutations.

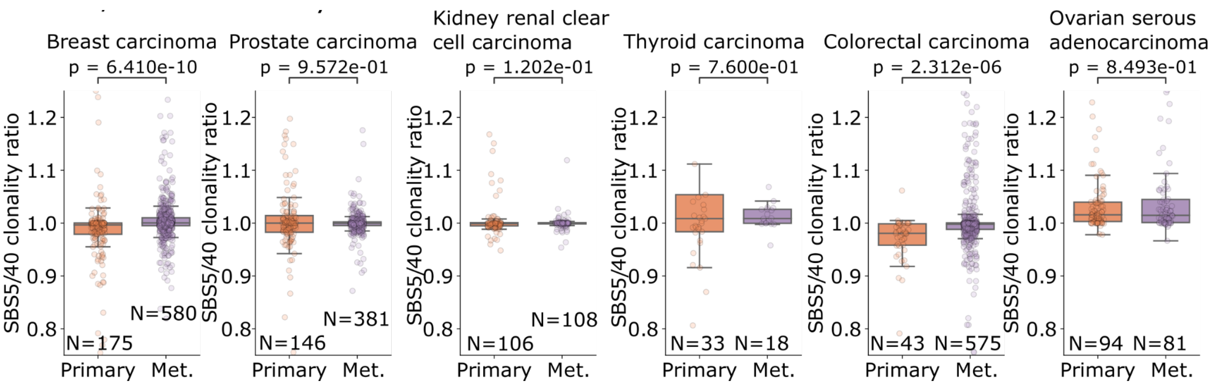

Supplementary Figure 48: Comparison of the global SBS5 and SBS40 combined clonality ratios between primary and metastatic tumors across the cancer type of interest. Boxplots are defined as in Suppl. Fig. 45. P, two-sided Mann-Whitney p-value. N, number of samples.

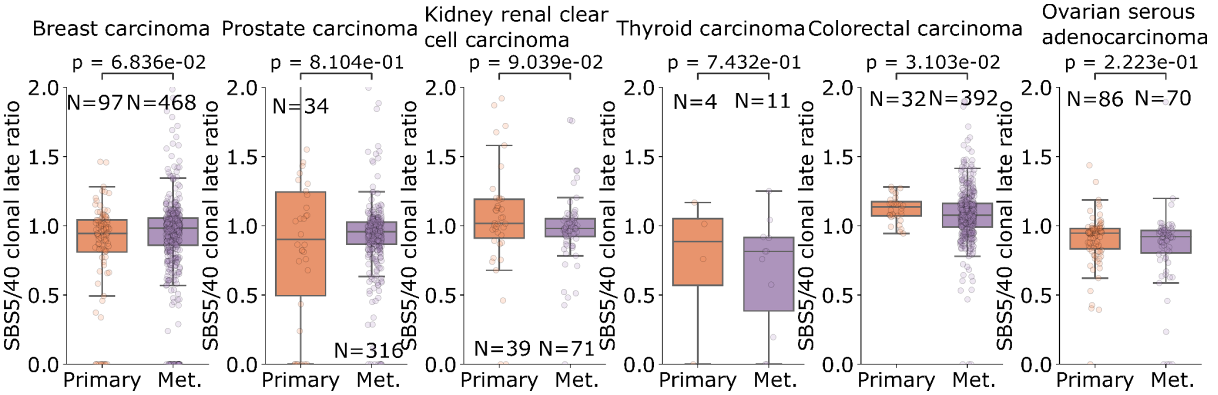

Supplementary Figure 49: Comparison of the global SBS5 and SBS40 combined clonal late ratios between primary and metastatic tumors across the cancer type of interest. Boxplots are defined as in Suppl. Fig. 45. P, two-sided Mann-Whitney p-value. N, number of samples.

Our comparison revealed that metastatic tumors did not show a relative increase in SBS5/SBS40 subclonal nor clonal late mutations compared to their primaries (Suppl. Fig. 48 and Suppl. Fig. 49). Therefore, the observed pattern for SBS1 mutations did not seem to be consistent for other clock-like mutational processes.

Altogether, this suggests that SBS1 and SBS5/SBS40 associated mutational processes have different underlying mutation dynamics and that such dynamics may be modified over the course of tumorigenesis.

Independent datasets description

We next aimed to leverage independent and publicly available datasets to further validate our observations. To do so, we searched in literature for datasets including paired primary and metastatic biopsies of cancer types of interest (i.e., breast, prostate, renal clear cell and thyroid) that fitted with the clinical characteristics of our primary and metastatic datasets. That is that primary tumors should have been biopsied at early stages, preferentially at the time of diagnosis and that metastatic tumors are late-stage tumors taken several months/years after primary diagnosis.

We initially prioritized cohorts undergoing WGS as it provides the best resolution to perform mutational signature analysis that requires more than hundreds of passenger mutations. However, due to the scarcity of available datasets we also extended the search to whole-exome sequencing data cohorts. We excluded clinical panel sequencing cohorts because they are not suitable to analyze the differences in SBS1 mutation burden due the limited number of SBS1 mutations mapping to the sequenced regions.

We still realized that, even after extending the search to WES, there was a limited number of primary-metastatic paired datasets with accessible somatic mutation calling data. Consequently, we decided to extend the search to unpaired primary and/or metastatic WGS/WES datasets that can be used as independent validations of our observations

The following table describes the independent datasets used in the validation:

Cohort	Cancer type/s	Tumor stage	Sequencing platform	Is paired	Reference (DOI)
Lucy R Yates et al.	Breast cancer	Primary and metastatic	WGS	Yes	10.1016/j.ccell.2017.07.005[18]
TRACERx Renal	kidney renal clear cell carcinoma	Primary and metastatic	WES	Yes	10.1038/ng.2891[19,20]
TCGA	Breast, Kidney, Prostate, Thyroid, Colorectal, Ovarian	Primary	WES	No	10.1038/ng.2764[21]
SU2C-PCF	Prostate	Metastatic	WES	No	10.1073/pnas.1902651116[22]

David Quigley et al.	Prostate cancer	Metastatic	WGS	No	10.1016/j.cell.2018.06.039[23]
Diana Miao et al.	kidney renal clear cell carcinoma	Metastatic	WES	No	10.1126/science.aan5951[24]

Independent validation in using paired biopsies

Breast cancer dataset (Lucy R Yates et al.)

We gathered from the study performed by Lucy R Yates et al. 2017[18] the somatic mutation calls and clinical data for 17 patients that underwent whole-genome sequencing of multiple tumor biopsies.
We then filtered out patients lacking the clinical characteristics associated with our primary and metastatic cohort. More in detail, we excluded local recurrence and synchronous metastatic tumor patients because they do not resemble the nature of our primary and metastatic cohort. Moreover, we filtered out the PD11458 patient because it has been pre-treated prior to primary biopsy. Finally, tumors from PD8948 seemed to be genomically unrelated, rendering them unsuitable for our study. In total, 5 patients with both primary and metastatic paired biopsies were used in our analysis (see table below).

**Patient**	**Primary biopsy**	**Metastatic biopsy**	**Included**
PD13596	PD13596a	PD13596c	Yes
PD9193	PD9193a	PD9193c	Yes
PD4243a	PD4243a	PD4243c	Yes

PD11460	PD11460d	PD11460a	Yes, selected PD11460a because it is the largest time to met. biopsy
PD9771	PD9771d	PD9771e	Yes
PD8948	-	-	No, genomically unrelated biopsies.
PD11458	-	-	No, patient pre-treated before primary biopsy
PD9194	-	-	No, local relapse
PD9195	-	-	No, local relapse.
PD11461	-	-	No, local relapse
PD14780	-	-	No, synchronous met.
PD4820	-	-	No, synchronous met.
PD5956	-	-	No, synchronous met.
PD6728	-	-	No, synchronous met.
PD11459	-	-	No, synchronous met.
PD4252	-	-	No, synchronous met.
PD4248	-	-	No, synchronous met.

First, the primary and metastatic samples were represented alongside the primary and metastatic regression lines fitted with our primary and metastatic breast cohorts, respectively (see below).

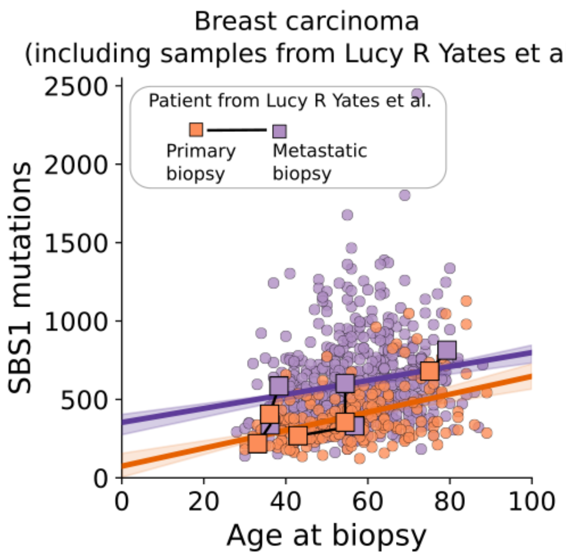

Supplementary Figure 50: Linear regression of the SBS1 mutation burden (y-axis) and patient’s age at biopsy (x-axis) in primary (orange) and metastatic (purple) breast carcinoma tumors from PCAWG and Hartwig, respectively. Joint orange and purple squares represent primary and metastatic paired biopsies from the lucy R Yates et al. breast carcinoma cohort. Orange dots represent primary tumor samples from the PCAWG cohort and purple dots metastatic tumor samples from Hartwig dataset. The median trendline and 99% confidence intervals of the linear regression are represented as a solid line and the adjacent shaded area, respectively.

Our data show that both primary and metastatic tumors fit within the expected SBS1 burden distributions according to their age at biopsy (i.e., non-significant difference distribution between the primary and metastatic biopsies with our primary and metastatic cohorts, respectively; primary Mann-Whitney p-value=0.37 and metastatic Mann-Whitney p-value=0.91, Suppl. Fig. 50).

Importantly, metastatic tumors showed a substantial SBS1 yearly mutation rate in metastasis compared to their primary counterparts (paired sample t-test P-value = 0.05, Suppl. Fig. 51). Furthermore, four out of five biopsies (except for PD13596) show a strong SBS1 yearly mutation rate increase in metastatic tumors.

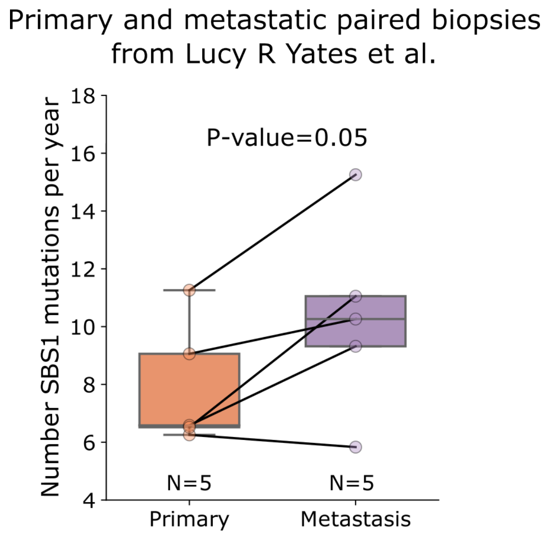

Supplementary Figure 51: Comparison of the age-corrected SBS1 burden between primary (orange) and metastatic (purple) biopsies from the Lucy R Yates et al. cohort. Each patient’s primary (orange dot) and metastatic (purple dot) paired biopsies are connected by a line. P-value, paired sample t-test p-value. Boxplots are defined as in Suppl. Fig. 50. N, number of samples.

It is important to remark that this comparison is likely an underestimation of the breast metastatic SBS1 mutation rate increase because the metastatic SBS1 burden of the 5 breast carcinomas samples used in this analysis, also includes all somatic mutations acquired during primary tumor evolution, which likely underwent lower mutation rates. In summary, when looking at longitudinal samples from individual breast cancer patients we observed an SBS1 mutation rate increase that supports our observations using unpaired biopsies.

Kidney renal clear cell carcinoma dataset (TRACERx renal dataset)

The cohort originally described by Marco Gerlinger and colleagues in their TRACERx renal early studies (see ref[20] and later ref.[19]), included two kidney renal clear cell carcinoma (KIRC) patients with paired primary and metastatic biopsies that underwent whole-exome sequencing (EV001 and EV002). Consequently, these two patients brought an opportunity to orthogonally examine whether our observations are also supported by paired primary-met biopsies. However, there are few aspects that we would like to comment on because they are of critical importance to understand the limitations and implications of the results of this analysis. First, the sample size is very limited (only two patients, although with multiple primary and metastatic biopsies) which significantly hampers the statistical interpretation of the data. Therefore, we would mostly focus on the observed trends rather than the significance of our observations. Second, the patients included in this study only partially match with the clinical characteristics of our original cohort (e.g., metastatic biopsies are taken shortly after primary biopsies (6-15 weeks), which are likely earlier than the overall clinical characteristics of the Hartwig metastatic renal clear cell carcinoma dataset that includes patient biopsies typically several months or years after primary cancer diagnosis. Third, patients's tumors have been sequenced using whole-exome sequencing with

sequencing standards from 2012, rendering the comparison with our current cohort very challenging. In any case, given the scarcity of primary-met paired biopsies we decided to further proceed with the analysis.

We decided to perform somatic calling ourselves due to the unavailability of all WES characterized somatic mutations (personal communication with the authors). Therefore, after being granted access of the renal dataset from the CRICK (ref 2022-0322), we downloaded the raw sequencing data of the germline and tumor samples from the two aforementioned patients (EGA access identifier EGAS00001000667, patients EV001 and EV002). In total 19 samples were successfully downloaded, including 11 for EV001 (M1, M2a, M2b, R1, R2, R3, R4, R5, R8 and R9) and 8 for EV002 (M, R1, R3, R4, R6, R7 and R9). Somatic calling was performed using SAGE, which is the somatic mutation caller from the Hartwig analytical pipeline. Parameters were adjusted for lower read quality (MIN_TUMOR_QUAL>=120) to align the non-synonymous mutation burden with the original publication. Clinical data and patient’s age were respectively obtained from Figure 1 and the Supplementary Appendix of the 10.1056/NEJMoa1113205 publication.
The metastatic sample M2b from EV001 was discarded due to low overall sequencing coverage (see Appendix from ref[20]). Finally, sample specific SBS1 counts were calculated as in the rest of analyses (see main methods).

Then, following the methodology described for the breast cancer paired dataset, we decided to compare whether the SBS1 mutation rate was different between primary and metastatic biopsies (Suppl. Fig. 52).

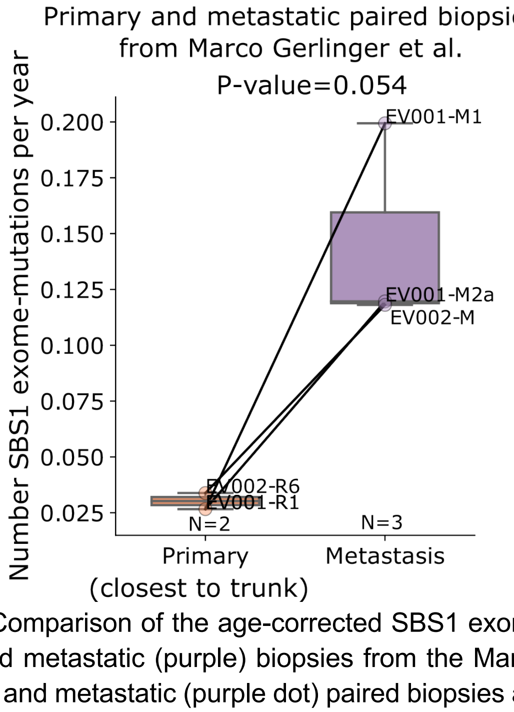

Supplementary Figure 52: Comparison of the age-corrected SBS1 exome overlapping mutation burden between primary (orange) and metastatic (purple) biopsies from the Marco Gerlinger et al. cohort. Each patient’s primary (orange dot) and metastatic (purple dot) paired biopsies are connected by a line. P-value, paired sample t-test p-value. Boxplots are defined as in Suppl. Fig. 50. N, number of samples.

Our data revealed an important increase in SBS1 mutation rate in metastatic samples compared to the primary samples that are closest to the most recent common ancestor (i.e., less primary-exclusive mutations, evolutionary distance extracted from Figure 3 of ref[19]).

This enrichment could be explained by an increased global mutation rate of metastatic tumors and/or by a relative increase of SBS1 mutation rate in later stages. To evaluate this, we compared the relative SBS1 mutation rate between early stage mutations (i.e., those shared by ⅔ of the primary samples of the same donor, likely generated before or at early stages of malignant transformation) and the metastatic stage (i.e., defined as those metastatic mutations not present in any primary sample and thus likely generated after metastatic colonization). Our data revealed that SBS1 mutation rate is relatively enriched in later tumorigenic stages (see Suppl. Fig. 53 below).

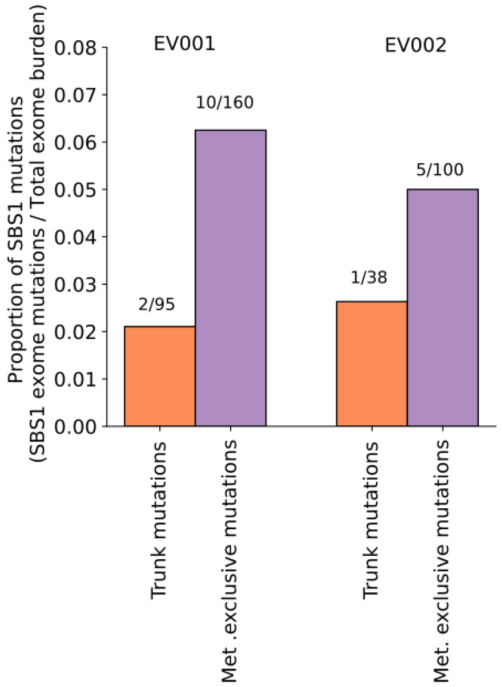

Supplementary Figure 53: Comparison of the proportion of SBS1 exon overlapping mutations shared across primary biopsies (orange bar) and metastatic-exclusive biopsies (purple bars). Left, EV001 patient; and right, EV002 patient from the Marco Gerlinger et al. cohort. Top label numbers depict the number of SBS1 mutations divided by the total of mutations.

Finally, it is also important to remark that that the original publication already noticed an increased SBS1 mutation rate in later tumorigenic stages (quoted from the results section of the Nature Genetics manuscript[19]):

“The proportion of C>T transitions at CpG sites increased significantly in branches (q = 0.007; Fig. 5b). This increase was observed in eight of the ten cases (Fig. 5c). The proportion of C>T

transitions increased in all XpCpG contexts, where X represents any nucleotide (Supplementary Fig. 10).”

See below Fig. 5b from the original publication:

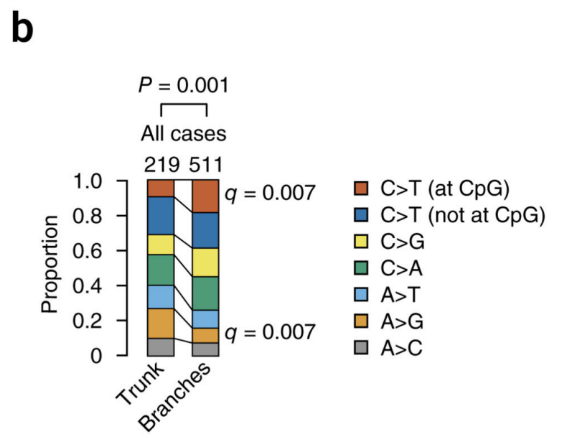

Fig. 5b from Marco Gerlinger et al. DOI: 10.1038/ng.2891

In summary, this data suggest that metastatic kidney renal clear cell carcinoma tumors have higher rate of SBS1 mutation accumulation in advanced tumorigenic stages and that this increase is not solely explained by an overall increase in mutation rate at the metastatic stage. This observation is in line with the age-corrected increase in SBS1 mutation burden originally observed in our kidney renal clear cell carcinoma cohorts using unpaired tumor samples.

Independent validation using external primary and metastatic datasets

Due to the limited availability of paired primary and metastatic datasets matching our cohort characteristics, we have decided to expand the validation by relying on independent unpaired primary and metastatic datasets. The rationale for this is that by proving that our observations are sustained with independent patients, we would help to rule out the possibility that the SBS1 metastatic increase is caused by some underlying reasons exclusive to our primary or metastatic cohorts as well as by the tumor analytical pipeline used.

Breast carcinoma

Given the popularity and the magnitude of the breast cancer TCGA cohort (including more than 900 tumor samples) we decided to compare the TCGA SBS1-age regression (as a representative of primary untreated breast cancer tumors) with the Hartwig SBS1-age regression (metastatic

cohort). Importantly, to render the two cohorts comparable we restricted the analysis to exome overlapping mutations (i.e., TCGA has been sequenced using WES). To do so, we created a whole-exome version of the Hartwig breast cancer cohort by selecting exome overlapping mutations according to the genomic coordinates provided by Agilent Human all exome v5 using hg19 as reference genome (see Appendix1 and the end of the document for further details).

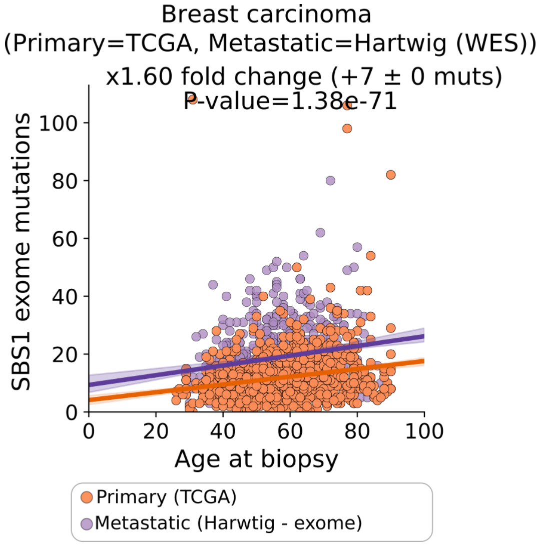

Supplementary Figure 54: Linear regression of the exome-overlapping SBS1 mutation burden (y-axis) and patient’s age at biopsy (x-axis) in primary TCGA (orange) and metastatic breast carcinoma tumors from Hartwig (purple). The mean fold change, mean SBS1 increase per year and one-sided Mann-Whitney p-value are displayed at the top of the chart. The median trendline and 99% confidence intervals of the linear regression are represented as a solid line and the adjacent shaded area, respectively.

Our results revealed that using the TCGA breast cancer dataset as the representative cohort the untreated primary tumors rendered highly consistent patterns of age-independent SBS1 enrichment (Suppl. Fig. 54 above, average fold change x1.60, p-value=1.38e-87 compared to average fold change x1.46 based on our original calculations; Extended Data Fig. 4a).

Prostate carcinoma

We used the WGS metastatic prostate cancer cohort published by David Quigley et al.[23] as a representative of metastatic prostate tumors. To do so, we first downloaded the somatic mutation calls from 101 metastatic prostate samples and clinical data from their repository (https://quigleylab.ucsf.edu/data). Then we assessed whether the metastatic prostate samples from this cohort matched with the expected distribution given the fitted regression line by our metastatic cohort (see below).

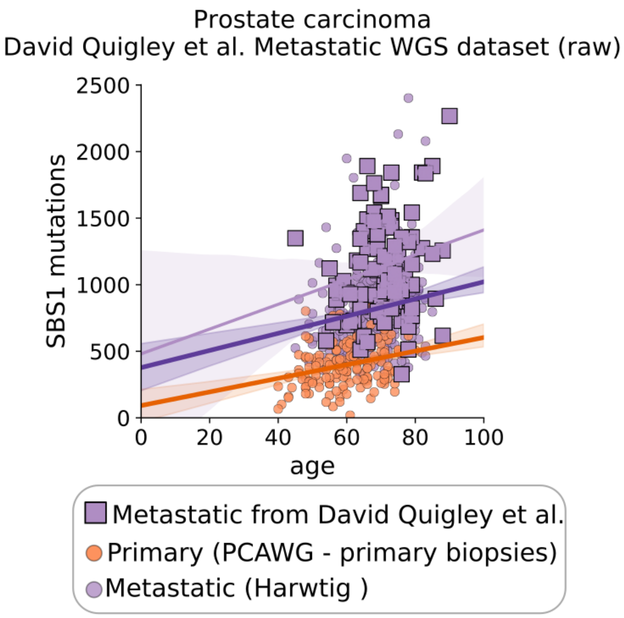

Supplementary Figure 55: Linear regressions of the raw SBS1 mutation burden (y-axis) and patient’s age at biopsy (x-axis) from primary prostate carcinoma tumors from PCAWG (orange dots and line), metastatic prostate carcinoma tumors from Hartwig (purple dots and thicker purple line) and metastatic prostate carcinoma tumors from David Quiegley et al. (purple squares and thinner purple line). The median trendline and 99% confidence intervals of the linear regression are represented as a solid line and the adjacent shaded area, respectively.

We observed a systematic bias towards an increased SBS1 mutation burden in the metastatic David Quigley et al. cohort compared to Hartwig (Suppl. Fig. 55, lighter purple regression line, Mann-Whitney p-value < 0.01). Therefore, to rule out the possibility of an increased SBS1 burden in metastatic tumors due to a different mutation calling sensitivity compared to our pipeline, we reprocessed samples from their study using the Hartwig mutational calling pipeline. After granted access to the raw data (phs001648/GRU) via dbGAP, we downloaded 10 randomly selected samples (p128, p135, p140, p167, p213, p265, p53, p55, p89, p90) and ran them through the Hartwig pipeline in the google cloud environment. It turned out that the mutation calls of the 10 reprocessed samples with the Hartwig pipeline showed substantially lower SBS1 mutation burden compared to the results from their original mutation calls (average fold change of x1.395 across the ten samples, fold change calculated as the ratio between the original SBS1 counts and the SBS1 counts provided by our processing pipeline). To account for this we decided to apply a normalization factor of 1.395 to the original SBS1 burden of all 101 metastatic prostate samples, which aims to mitigate the biases introduced by difference mutation calling sensitivities.

After applying the normalization factor to the SBS1 burden of metastatic samples from the David Quigley et al. cohort the two metastatic SBS1 vs. age regression lines were in near perfect agreement (see Suppl. Fig. 56 below).

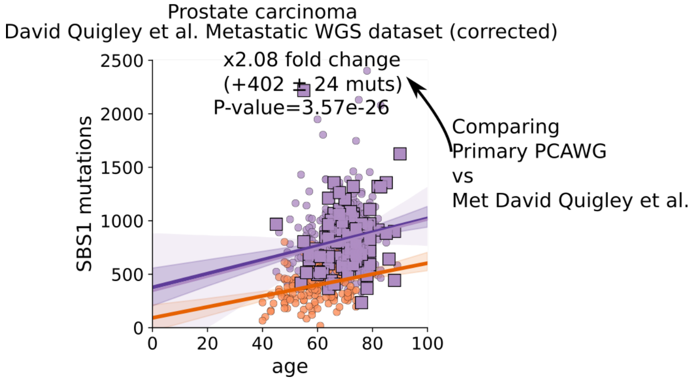

Supplementary Figure 56: Linear regressions of the technically adjusted SBS1 mutation burden (y-axis) and patient’s age at biopsy (x-axis) from primary prostate carcinoma tumors from PCAWG (orange dots and line), metastatic prostate carcinoma tumors from Hartwig (purple dots and thicker purple line) and metastatic prostate carcinoma tumors from David Quiegley et al. (purple squares and thinner purple line). The mean fold change, mean SBS1 increase per year and one-sided Mann-Whitney p-value are displayed at the top of the chart. The median trendline and 99% confidence intervals of the linear regression are represented as a solid line and the adjacent shaded area, respectively.

As expected, this also entailed a strong and age-independent enrichment in SBS1 mutation burden in metastatic tumors from this cohort compared to primary tumors from PCAWG (average fold change of x2.08 compared to average fold change of x2.00 using Hartwig as metastatic cohort; Extended Data Fig. 4a).

Next, following the procedure described for the breast cancer dataset, we relied on the TCGA prostate cancer primary dataset as an independent cohort of primary prostate untreated tumors. Similarly, we downloaded the mutation calls from the SU2C-PCF cohort [22] including 444 metastatic castrate resistant prostate cancer WES samples to perform a comparison with the exome overlapping mutation calls from TCGA (see Suppl. Fig. 57 below).

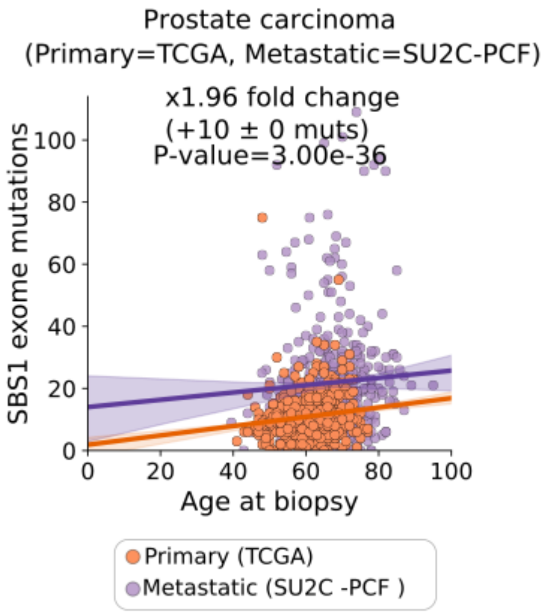

Supplementary Figure 57: Linear regression of the exome-overlapping SBS1 mutation burden (y-axis) and patient’s age at biopsy (x-axis) in primary TCGA (orange) and metastatic prostate carcinoma tumors from the SU2C - PCF study (purple). The mean fold change, mean SBS1 increase per year and one-sided Mann-Whitney p-value are displayed at the top of the chart. The median trendline and 99% confidence intervals of the linear regression are represented as a solid line and the adjacent shaded area, respectively.

Consistently, the comparison revealed an age-independent SBS1 mutation burden increase in the metastatic cohort (DFCI) compared to the primary (TCGA). The extent of the enrichment (average x1.96 fold change) was well aligned with the originally observed increase in our cohort (x2.00 fold change, Extended Data Fig. 4a).

Kidney renal clear cell carcinoma

Aside from the comparison using paired primary-metastatic biopsies from TRACERx renal (see above) we also performed a comparison of the SBS1-age regressions of two independent kidney clear carcinoma primary and metastatic cohorts. Specifically, we relied on the TCGA kidney renal clear cell carcinoma as representative of primary untreated tumors whereas we used the 35 metastatic samples published by Diana Miao and colleagues [24] as the metastatic cohort.

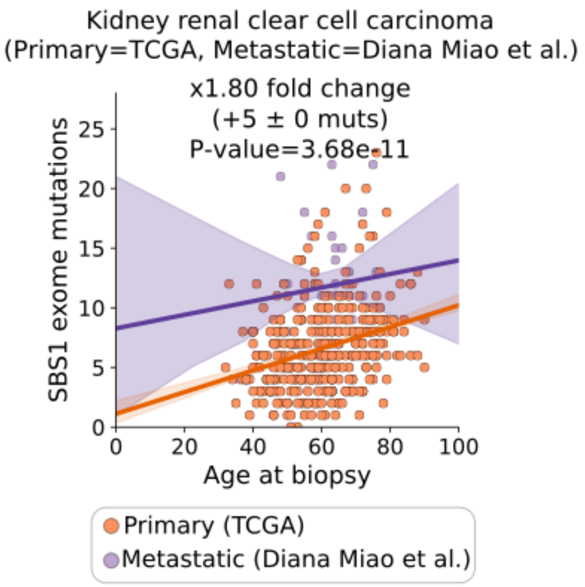

Supplementary Figure 58: Linear regression of the exome-overlapping SBS1 mutation burden (y-axis) and patient’s age at biopsy (x-axis) in primary TCGA (orange) and metastatic renal clear cell carcinoma cohort from the Diana Miaoi et al. study (purple). The mean fold change, mean SBS1 increase per year and one-sided Mann-Whitney p-value are displayed at the top of the chart. The median trendline and 99% confidence intervals of the linear regression are represented as a solid line and the adjacent shaded area, respectively.

The results from this comparison were also in agreement with the initial observation using PCAWG and Hartwig as primary and metastatic cohorts (Suppl. Fig. 58), respectively (average fold change x1.80 using these cohorts, x1.51 average fold change relying on our cohorts, both are statistically significant; see Extended Data Fig. 4a). Overall, our data shows that the increase in metastatic renal clear cell carcinomas is consistent across different primary and metastatic cohorts, which further supports the robustness of our main results.

Thyroid carcinoma

We also analyzed the SBS1-age distribution pattern differences between thyroid primary tumors TCGA compared to the Hartwig cohort (exome-overlapping mutations only).

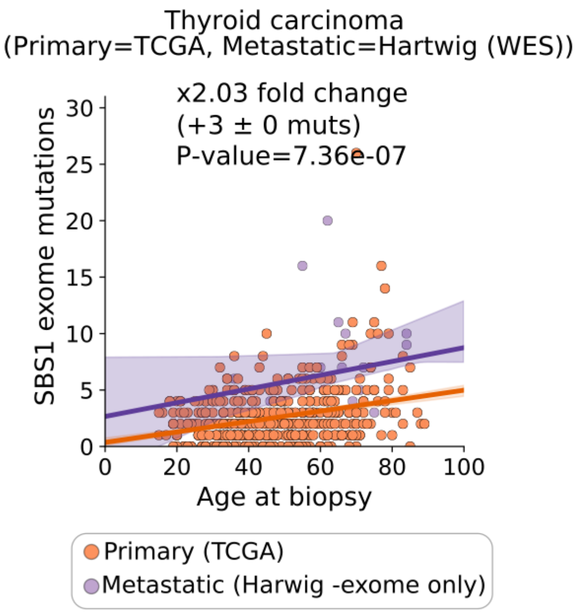

Supplementary Figure 59: Linear regression of the exome-overlapping SBS1 mutation burden (y-axis) and patient’s age at biopsy (x-axis) in primary TCGA (orange) and metastatic Hartwig (purple) thyroid carcinoma cohorts. The mean fold change, mean SBS1 increase per year and one-sided Mann-Whitney p-value are displayed at the top of the chart. The median trendline and 99% confidence intervals of the linear regression are represented as a solid line and the adjacent shaded area, respectively.

In agreement with other cancer types and with our original observations metastatic tumors from Hartwig also show a significant and age-independent increase in SBS1 mutation burden compared to primaries from TCGA (Suppl. Fig. 59, average x2.03 fold change compared to average x2.05 in our cohort; both statistically significant; Extended Data Fig. 4a).

Colorectal and ovarian serous carcinomas

We also tested whether the pattern of highly consistent SBS1 mutation rate patterns between primary and metastatic colorectal and ovarian tumors was maintained when relying on primary tumors from the TCGA cohort.

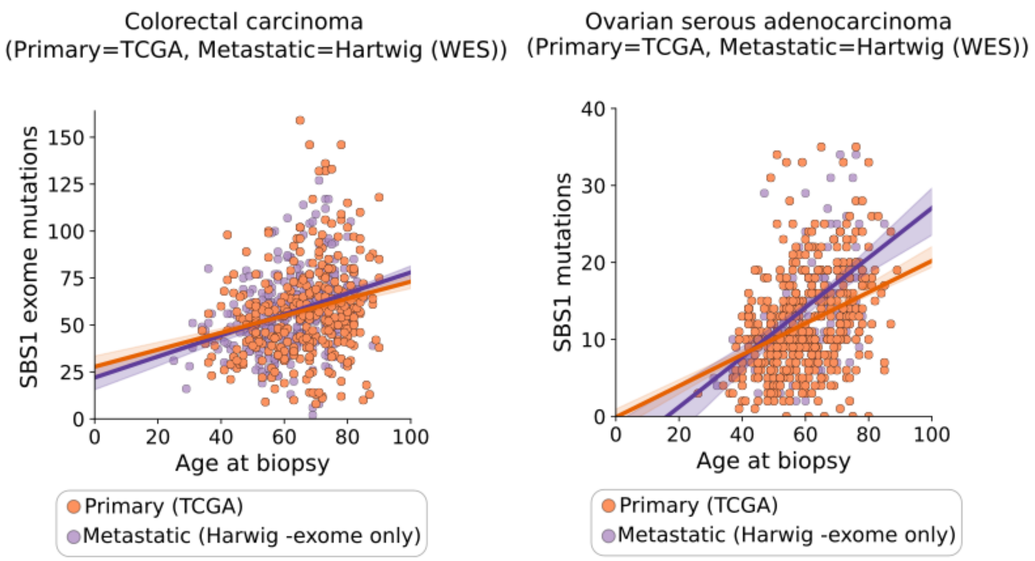

Supplementary Figure 60: Linear regression of the exome-overlapping SBS1 mutation burden (y-axis) and patient’s age at biopsy (x-axis) in primary TCGA (orange) and metastatic Hartwig (purple) cohorts of colorectal carcinoma (left) and ovarian serous adenocarcinoma patients. The median trendline and 99% confidence intervals of the linear regression are represented as a solid line and the adjacent shaded area, respectively.

As shown by Suppl. Fig. 60, both age-SBS1 distributions are in perfect agreement, which further supports our initial observation relying on the WGS PCAWG dataset as the primary cohort (Extended Data Fig. 4a).

Conclusions

All analyses performed in this supplementary note are in strong agreement with the observations explained in the main text and further support the rationale of a cancer type specific increase of the clock-like SBS1 mutation rate in metastatic tumors of breast, renal clear cell, prostate and thyroid carcinomas compared to their primary counterparts.

More in detail, this increase does not seem to be explained by higher genome ploidy, was not driven by a specific cancer subtype (at least in breast cancer), showed an enrichment towards recently acquired mutations (i.e., clonal late and subclonal) and was also observed in individual patients from breast and kidney renal clear cell carcinomas. Finally, relying on independent cohorts -with different mutation calling pipelines- provided highly consistent patterns.

To conclude, since this phenomenon was not observed for cell-division independent clock-like mutational processes (i.e., SBS5/SBS40), we believe that it can be tightly associated with an increased cell division rate of metastatic tumors, given its known association with SBS1 mutation rates [16]. Such an increase would be particularly relevant for slowly dividing primary tumors (e.g., prostate, thyroid, kidney renal clear cell and breast carcinomas) where an increased cell division rate would lead to higher pace of clock-like SBS1 mutation accumulation (see Extended Data Fig. 4e, g-h). Nevertheless, we can not entirely rule out that this increase is caused by other cancer type specific factors such as higher rates of 5-methylcytosine deamination, decreased fidelity to repair these mismatches or higher contribution from metastatic enriched mutational processes with overlapping mutational contexts.

Appendix 1: Exome-only mutations in Hartwig dataset

To get the exome-overlapping mutations of Hartwig we intersected the somatic mutation .VCF files with the .bed files representing the human exome coordinates (Agilent Human exome v.5) using the bedtools (v.2.27.1) intersect function. Intersected mutations were stored in separate files and used for comparisons against whole-exome datasets.

Supplementary Note 4

Overview 100
TEDs rationale and limitations 100
Prognostic and resistance TEDs 100
TEDs and primary targets of targeted therapies 100

Overview

This supplementary note expands the results and methods of the TEDs section from the main texts. Hence, we describe here some of the results and discussions that could not be fit into the main text given the length constraints.

TEDs rationale and limitations

The main rationale behind the TEDs analyses is to detect genes that are subjected to positive selection in a population of patients treated with a specific drug in a cancer type specific manner. Hence, to rule out cancer driver genes that were positively selected in the primary clonal expansion of the tumor, we controlled for their mutation frequency in the primary untreated group (i.e., PCAWG primary cohort in our dataset). As a result of our analyses we obtained a list of treatment-enriched drivers (TEDs) that were significantly enriched in a treated cohort of patients.

This analysis has several caveats that we next discuss.

Prognostic and resistance TEDs

First, some of the TEDs may be associated with an overall worse prognosis of the metastatic tumors, rather than representing a treatment-resistance specific signature. For instance, we detected that TP53 was recurrently found across several treatment-cancer type groups, which likely indicates that TP53 alterations may lead into relatively more aggressive tumors, but not necessarily represents a treatment-specific mechanism of resistance (although we can not rule out this possibility). In order to label those TEDs that likely represent a prognosis-factor, we have annotated all TEDs with the *treatment-exclusive* label, which indicate whether the TED is exclusively found in the treated group, which is more likely to represent a genuine mechanism of resistance. Non-exclusive TEDs were labeled as *treatment-enriched* in results and in Supp. Table 8.

TEDs and primary targets of targeted therapies

While the majority of TEDs are exclusively mutated in the treated cohort group, certain TEDs are *de-facto* original targets of the anti-cancer treatment. For instance, *EGFR* inhibitors are generally given to non-small cell lung cancer (NSCLC) patients bearing *EGFR* alterations. In our dataset, we detected three TEDs that are also the original targets of the cancer treatment: *EGFR* mutations in the Anti-EGFR therapy group in NSCLC, *BRAF* mutations in the Anti-*BRAF* therapy group in skin melanoma, and *ERBB2* mutations in the Anti-*ERBB2* therapy group of breast carcinoma patients. These cases are marked in Supp. Table 8 (*is_drug_target* column). Naturally, the

identification of these TEDs is expected given that the presence of the mutated target is a prerequisite for the treatment initiation. However, it is important to consider that secondary target mutations are one of the most common mechanisms of resistance to targeted therapies. For instance, *EGFR*^{T790M} and *EGFR*^{C797S} mutations are the main mechanisms of resistance to *EGFR* TKIs[25,26], whereas secondary *BRAF*^{L514V} mutations may confer resistance to *BRAF* inhibitors[27]. Thus, to further provide insights into these three cases we have performed an additional analyses at the variant level, aiming at identifying alterations that were exclusively found in the treated group (i.e., the primary untreated group eligible for treatment did not harbor any of these alterations).

This analysis revealed that *EGFR* and *ERBB2* harbored secondary mutations that were exclusively found in the treated group (see table below, now part of the Supp. Table 8) (e.g., *EGFR*^{T790M},*EGFR*^{C797S},*ERBB2*^{V777L}, etc.) whereas we did not observe *BRAF* secondary mutations in our cohort. Of note, none of the secondary mutations were significantly enriched in the metastatic cohort, likely owing to the low sample size.

**gene**	**mutation**	**drug**	**cancer_type_code**	**samples_treated_mutation_found**	**total_samples_treated_gene_mutated**	**samples_untreated_mutation_found**	**total_samples_untreated_gene_mutated**	**odds_ratio**	**G-test pvalue**	**is_exlusive_treatment**
ERBB2	L755S	Anti_HER2	BRCA	2	12	0	3	5.0	0.780304120501725	True
ERBB2	V777L	Anti_HER2	BRCA	2	12	0	3	5.0	0.780304120501725	True
EGFR	.	Anti_EGFR	LUAD	20	78	3	6	0.5128205128205128	0.6412365052849492	False
EGFR	A750P	Anti_EGFR	LUAD	2	78	0	6	1.5384615384615383	0.4185048015897672	True
EGFR	C797S	Anti_EGFR	LUAD	8	78	0	6	6.153846153846153	0.9738570382557281	True
EGFR	G719A	Anti_EGFR	LUAD	2	78	0	6	1.5384615384615383	0.4185048015897672	True
EGFR	G719S	Anti_EGFR	LUAD	2	78	0	6	1.5384615384	0.4185048015	True

								615383	897672	
EGFR	L858R	Anti_EGFR	LUAD	12	78	0	6	9.230769230769232	0.7372432683994703	True
EGFR	L861Q	Anti_EGFR	LUAD	2	78	1	6	0.15384615384615385	0.61924653056131	False
EGFR	T790M	Anti_EGFR	LUAD	18	78	0	6	13.846153846153847	0.5021076742591722	True
BRAF	.	BRAF_inhibitor	SKCM	2	13	15	56	0.5743589743589744	0.7357383238287619	False
BRAF	V600E	BRAF_inhibitor	SKCM	11	13	31	56	1.5285359801488834	0.5026222962196717	False

Overall, our analysis suggests that the global mutation frequency of *EGFR* and *ERBB2* in the treated cohorts can not solely be explained by the design of the analysis, and thus include mutations associated with treatment resistance.

References

1. Priestley, P. *et al.* Pan-cancer whole-genome analyses of metastatic solid tumours. *Nature* **575**, 210–216 (2019).
2. Consortium, T. I. P.-C. A. of W. G. & The ICGC/TCGA Pan-Cancer Analysis of Whole Genomes Consortium. Pan-cancer analysis of whole genomes. *Nature* vol. 578 82–93 Preprint at https://doi.org/10.1038/s41586-020-1969-6 (2020).
3. Li, H. & Durbin, R. Fast and accurate short read alignment with Burrows-Wheeler transform. *Bioinformatics* **25**, 1754–1760 (2009).
4. Roepman, P. *et al.* Clinical Validation of Whole Genome Sequencing for Cancer Diagnostics. *J. Mol. Diagn.* **23**, 816–833 (2021).
5. Cameron, D. L. *et al.* GRIDSS2: comprehensive characterisation of somatic structural variation using single breakend variants and structural variant phasing. *Genome Biol.* **22**, 1–25 (2021).
6. Shale, C. *et al.* Unscrambling cancer genomes via integrated analysis of structural variation and copy number. *Cell Genomics* **2**, 100112 (2022).
7. Hoadley, K. A. *et al.* Cell-of-Origin Patterns Dominate the Molecular Classification of 10,000 Tumors from 33 Types of Cancer. *Cell* **173**, 291–304.e6 (2018).
8. Tarasov, A., Vilella, A. J., Cuppen, E., Nijman, I. J. & Prins, P. Sambamba: fast processing of NGS alignment formats. *Bioinformatics* **31**, 2032–2034 (2015).
9. Taylor, A. M. *et al.* Genomic and Functional Approaches to Understanding Cancer Aneuploidy. *Cancer Cell* **33**, 676–689.e3 (2018).
10. Yachida, S. *et al.* Comprehensive Genomic Profiling of Neuroendocrine Carcinomas of the Gastrointestinal System. *Cancer Discov.* **12**, 692–711 (2022).
11. Mateo, J. *et al.* Genomics of lethal prostate cancer at diagnosis and castration resistance.

J. Clin. Invest. **130**, 1743–1751 (2020).

12. Reiter, J. G. *et al.* Lymph node metastases develop through a wider evolutionary bottleneck than distant metastases. *Nat. Genet.* **52**, 692–700 (2020).
13. Nguyen, B. *et al.* Genomic characterization of metastatic patterns from prospective clinical sequencing of 25,000 patients. *Cell* **185**, 563–575.e11 (2022).
14. Blokzijl, F. *et al.* Tissue-specific mutation accumulation in human adult stem cells during life. *Nature* **538**, 260–264 (2016).
15. Abascal, F. *et al.* Somatic mutation landscapes at single-molecule resolution. *Nature* **593**, 405–410 (2021).
16. Alexandrov, L. B. *et al.* Clock-like mutational processes in human somatic cells. *Nat. Genet.* **47**, 1402–1407 (2015).
17. Gerstung, M. *et al.* The evolutionary history of 2,658 cancers. *Nature* **578**, 122–128 (2020).
18. Yates, L. R. *et al.* Genomic Evolution of Breast Cancer Metastasis and Relapse. *Cancer Cell* **32**, 169–184.e7 (2017).
19. Gerlinger, M. *et al.* Genomic architecture and evolution of clear cell renal cell carcinomas defined by multiregion sequencing. *Nat. Genet.* **46**, 225–233 (2014).
20. Gerlinger, M. *et al.* Intratumor heterogeneity and branched evolution revealed by multiregion sequencing. *N. Engl. J. Med.* **366**, 883–892 (2012).
21. Cancer Genome Atlas Research Network *et al.* The Cancer Genome Atlas Pan-Cancer analysis project. *Nat. Genet.* **45**, 1113–1120 (2013).
22. Abida, W. *et al.* Genomic correlates of clinical outcome in advanced prostate cancer. *Proc. Natl. Acad. Sci. U. S. A.* **116**, 11428–11436 (2019).
23. Quigley, D. A. *et al.* Genomic Hallmarks and Structural Variation in Metastatic Prostate Cancer. *Cell* **174**, 758–769.e9 (2018).
24. Miao, D. *et al.* Genomic correlates of response to immune checkpoint therapies in clear cell renal cell carcinoma. *Science* **359**, 801–806 (2018).

25. Niederst, M. J. *et al.* The Allelic Context of the C797S Mutation Acquired upon Treatment with Third-Generation EGFR Inhibitors Impacts Sensitivity to Subsequent Treatment Strategies. *Clin. Cancer Res.* **21**, 3924–3933 (2015).
26. Yun, C.-H. *et al.* The T790M mutation in EGFR kinase causes drug resistance by increasing the affinity for ATP. *Proc. Natl. Acad. Sci. U. S. A.* **105**, 2070–2075 (2008).
27. Wang, J. *et al.* A Secondary Mutation in BRAF Confers Resistance to RAF Inhibition in a BRAFV600E-Mutant Brain Tumor. *Cancer Discov.* **8**, 1130–1141 (2018).
