# Supplementary material for: Pan-cancer whole-genome comparison of primary and metastatic solid tumours
Source: Nature. 2023 May 10;618(7964):333–41. doi: 10.1038/s41586-023-06054-z (PMC10247378; doi:10.1038/s41586-023-06054-z)
Supplement: Supplementary file 2 — Reporting Summary [file 41586_2023_6054_MOESM2_ESM.pdf]

nature portfolio

Corresponding author(s): Edwin Cuppen

Last updated by author(s): Mar 17, 2023

Reporting Summary

Nature Portfolio wishes to improve the reproducibility of the work that we publish. This form provides structure for consistency and transparency in reporting. For further information on Nature Portfolio policies, see our Editorial Policies and the Editorial Policy Checklist.

Statistics

For all statistical analyses, confirm that the following items are present in the figure legend, table legend, main text, or Methods section.

n/a	Confirmed	
☐	☒	The exact sample size (*n*) for each experimental group/condition, given as a discrete number and unit of measurement
☐	☒	A statement on whether measurements were taken from distinct samples or whether the same sample was measured repeatedly
☐	☒	The statistical test(s) used AND whether they are one- or two-sided<br>*Only common tests should be described solely by name; describe more complex techniques in the Methods section.*
☒	☐	A description of all covariates tested
☐	☒	A description of any assumptions or corrections, such as tests of normality and adjustment for multiple comparisons
☐	☒	A full description of the statistical parameters including central tendency (e.g. means) or other basic estimates (e.g. regression coefficient) AND variation (e.g. standard deviation) or associated estimates of uncertainty (e.g. confidence intervals)
☐	☒	For null hypothesis testing, the test statistic (e.g. *F*, *t*, *r*) with confidence intervals, effect sizes, degrees of freedom and *P* value noted<br>*Give P values as exact values whenever suitable.*
☒	☐	For Bayesian analysis, information on the choice of priors and Markov chain Monte Carlo settings
☐	☒	For hierarchical and complex designs, identification of the appropriate level for tests and full reporting of outcomes
☐	☒	Estimates of effect sizes (e.g. Cohen's *d*, Pearson's *r*), indicating how they were calculated

Our web collection on statistics for biologists contains articles on many of the points above.

Software and code

Policy information about availability of computer code

Data collection

We have matched tumor-normal whole genome sequencing data from cancer patients from two independent cohorts: the Hartwig Medical Foundation (Hartwig) and the Pan-Cancer Analysis of Whole Genomes (PCAWG) cohort.

The Hartwig cohort was provided under data transfer agreement (DR-247) by Hartwig Medical Foundation on 6 February 2020 with an update received on 4 Februari 2022, that was downloaded using via Hartwig download portal.

The PCAWG-US was approved by National Institutes of Health (NIH) for the dataset General Research Use in The Cancer Genome Atlas (TCGA) on 25 February 2021 under application number 100344-3 and downloaded via dbGAP download portal.
Raw sequencing access to the non-US PCAWG samples was granted via the Data Access Compliance Office (DACO) Application Number DACO-1050905 on 6 October 2017 and downloaded via https://console.cancercollaboratory.org on 4 December 2017.

Data analysis

The PCAWG samples were reanalyzed with the Hartwig somatic variant calling pipeline (https://github.com/hartwigmedical/pipeline5) which was hosted on the Google Cloud Platform using Platinum (https://github.com/hartwigmedical/platinum). This pipeline uses the following software packages:
SamToFastq PICARD (v2.1.0).
BWA (v0.7.17): read mapping
GATK (v3.8.0) Haplotype Caller: calling germline variants in the reference sample
SAGE (v2.2): somatic SMNVs and indels calling
GRIDSS (v2.9.3): simple and complex structural variant calling
AMBER (v3.3)
COBALT (v1.7)

PURPLE (v2.53): combines B-allele frequency (BAF) from AMBER (v3.3), read depth ratios from COBALT (v1.7), and structural variants from GRIDSS to estimate copy number profiles, variant allele frequency (VAF) and variant clonality. PURPLE also determines sample gender based on sex chromosome ploidy.
LINX (v1.16): interpretation of simple mutations and structural variants
mutSigExtractor (v1.23)
SigProfilerExtractor (v1.1.1)
SnpEff (v5.1)
dNdScv (v0.0.1)
ActiveDriverWGS (v1.1.2)
GISTIC2 (v2.0.23)
MutationTimeR (v0.1)

The source code to reproduce the analysis of the manuscript is available in this repository https://github.com/UMCUGenetics/primary-met-wgs-comparison: https://github.com/UMCUGenetics/PCAWG_Hartwig_comparison

For manuscripts utilizing custom algorithms or software that are central to the research but not yet described in published literature, software must be made available to editors and reviewers. We strongly encourage code deposition in a community repository (e.g. GitHub). See the Nature Portfolio guidelines for submitting code & software for further information.

Data

Policy information about availability of data

All manuscripts must include a data availability statement. This statement should provide the following information, where applicable:

- Accession codes, unique identifiers, or web links for publicly available datasets
- A description of any restrictions on data availability
- For clinical datasets or third party data, please ensure that the statement adheres to our policy

Metastatic WGS data and metadata from the Hartwig Medical Foundation are freely available for academic use through standardized procedures. Request forms can be found at https://www.hartwigmedicalfoundation.nl/en/data/data-acces-request/

Somatic variant calls, gene driver lists, copy number profiles and other core data of the PCAWG cohort generated by the Hartwig analytical pipeline are available for download at https://dcc.icgc.org/releases/PCAWG/Hartwig. Researchers will need to apply to the ICGC data access compliance office (https://daco.icgc-argo.org) for the ICGC portion of the dataset. Similarly, users with authorized access can download the TCGA portion of the PCAWG dataset at https://icgc.bionimbus.org/files/5310a3ac-0344-458a-88ce-d55445540120. Additional information on accessing the data, including raw read files, can be found at https://docs.icgc.org/pcawg/data/.

GRCh37 reference genome used in this study: https://console.cloud.google.com/storage/browser/hmf-public/HMFtools-Resources/ref_genome/37

PCAWG clinical data: https://dcc.icgc.org/releases/PCAWG/clinical_and_histology
Consensus PCAWG mutation calls: https://dcc.icgc.org/releases/PCAWG/consensus_snv_indel
Consensus PCAWG mutation calls: https://dcc.icgc.org/releases/PCAWG/consensus_sv
Consensus PCAWG driver dataset: https://dcc.icgc.org/releases/PCAWG/driver_mutations

Independent validation datasets:
Primary and metastatic breast WGS data: DOI: 10.1016/j.ccell.2017.07.005 The link for the dataset is: http://dx.doi.org/10.17632/g7kpzkhz8c.1
Primary and metastatic kidney renal clear cell carcinoma WES data: 10.1038/ng.2891 Raw sequencing data was downloaded via: https://ega-archive.org/datasets/EGAD00001000734
Breast, Kidney, Prostate, Thyroid, Colorectal, Ovarian primary WES TCGA data:10.1038/ng.2764 downloaded via https://gdc.cancer.gov/about-data/publications/pancanatlas
Prostate metastatic WES data: 10.1073/pnas.1902651116 The link for the dataset is: https://github.com/cBioPortal/datahub/tree/master/public/prad_su2c_2019
Prostate metastatic WGS data: 10.1016/j.cell.2018.06.039 and downloaded via dbgap under phs001648.v2.p1
kidney renal clear cell carcinoma metastatic WES data: 10.1126/science.aan5951 and downloaded via https://www.cbioportal.org/

COSMIC reference signatures: https://cancer.sanger.ac.uk/signatures/
SIGNAL reference signatures: https://signal.mutationalsignatures.com/

OncoKB: https://www.oncokb.org/
CIViC: https://civicdb.org/
CGI: https://www.cancergenomeinterpreter.org/home

Human research participants

Policy information about studies involving human research participants and Sex and Gender in Research.

Reporting on sex and gender	Consistent gender proportions were observed across all cancer types except for thyroid adenocarcinomas, which had higher male representation in the metastatic cohort (metastatic: 72% male, 28% female; primary: 25% male, 75% female).
Population characteristics	The Hartwig cohort includes late-stage adult (>18 years old) cancer patients recruited across Dutch hospitals. Patients had frequently recived pre-biopsy treatment. The PCAWG cohort primarily include adult and early-stage cancer patients that in most cases have not recived any treatment prior to tumor biopsy. We refer to the Hartwig (doi: 10.1038/s41586-019-1689-y)

	and PCAWG (doi: 10.1038/s41586-020-1969-6.) flagship papers for further description of patient's population, recruitment and ethics oversight.
Recruitment	Patient recruitment was originally performed by the clinical institutions and hospitals. This study did not play any role in patient recruitment.
Ethics oversight	NA

Note that full information on the approval of the study protocol must also be provided in the manuscript.

Field-specific reporting

Please select the one below that is the best fit for your research. If you are not sure, read the appropriate sections before making your selection.

☒ Life sciences ☐ Behavioural & social sciences ☐ Ecological, evolutionary & environmental sciences

For a reference copy of the document with all sections, see nature.com/documents/nr-reporting-summary-flat.pdf

Life sciences study design

All studies must disclose on these points even when the disclosure is negative.

Sample size	We requested the data for all possible samples from the Hartwig and PCAWG cohorts. The Hartwig cohort included 4902 metastatic tumor samples from 4572 patients. The PCAWG cohort consisted of 2835 tumor samples from unique patients. After several filtering criteria we used 5,365 samples in the current study (1,914 from the PCAWG and 3,451 from the Hartwig cohort) to compare primary to metastatic tumors.
Data exclusions	A selection of samples for all analyses was made based on several criteria. To exclude duplicate samples from the same patient for the Hartwig cohort, we selected the tumor sample with the most recent biopsy date, and if this information did not exist we selected the sample with the highest tumor purity. However, some patients had biopsies from different primary tumor locations (likely independent or secondary tumors). In these cases, we kept at least one sample from each primary tumor location, and when there were multiple samples from the same primary tumor location, we applied the aforementioned biopsy date and tumor purity filtering criteria. For the PCAWG cohort, we processed one tumor sample per donor and tumor sample IDs are included in Supp. Table 1 of the manuscript. As with Hartwig QC filter criteria, samples with a tumor purity lower than 20% were removed as somatic variant calling was less reliable for these samples. PCAWG samples that were gray- or blacklisted by the PCAWG consortium were also removed (see https://dcc.icgc.org/releases/PCAWG/donors_and_biospecimens). For both cohorts, we only kept samples with >=50 SNVs/indels (likely no tumor cells present in the sample), and removed an additional set of samples for several reasons including failed variant calling, insufficient informed consent for use of the WGS data, unnatural SV landscape, and one duplicate PCAWG patient (DO217844) that was also included in the Hartwig cohort. After strict QC filtering, the PCAWG whitelisted cohort includes 2,376 samples and this dataset will be made available for the cancer research community via the PCAWG resource page. The metadata for every sample including those selected for analyses is detailed in supplementary table 1. Lastly, for this study, we only selected samples from cancer types with at least 15 samples that resulted in a final dataset consisting of 3,835 Hartwig samples and 1,916 PCAWG samples.
Replication	The source data and the source code used in this study are publicly available for academic purposes to ensure the reproducibility of the analysis conducted in this study
Randomization	Patients from both datasets (Hartwig and PCAWG) were independently recruited by clinical institutions and hospitals. Patients from the Hartwig Medical Foundation cohort represent late-stage cancer patients while PCAWG patients are primarily early-stage untreated cancer patients. This study did not play any role in patient's recruitment and randomization into experimental groups.
Blinding	This study did not play any role in patient's recruitment .

Reporting for specific materials, systems and methods

We require information from authors about some types of materials, experimental systems and methods used in many studies. Here, indicate whether each material, system or method listed is relevant to your study. If you are not sure if a list item applies to your research, read the appropriate section before selecting a response.

Materials & experimental systems

n/a	Involved in the study	
☒	☐	Antibodies
☒	☐	Eukaryotic cell lines
☒	☐	Palaeontology and archaeology
☒	☐	Animals and other organisms
☒	☐	Clinical data
☒	☐	Dual use research of concern

Methods

n/a	Involved in the study	
☒	☐	ChIP-seq
☒	☐	Flow cytometry
☒	☐	MRI-based neuroimaging
